# The Role of the Right Language Network and the Multiple‐Demand Network in Verbal Semantics: Insights From an Activation Likelihood Estimation Meta‐Analysis of 561 Functional Neuroimaging Studies

**DOI:** 10.1002/hbm.70415

**Published:** 2025-12-20

**Authors:** Eszter Demirkan, Francesca M. Branzi

**Affiliations:** ^1^ Department of Psychological Sciences Institute of Population Health, University of Liverpool Liverpool UK

**Keywords:** ALE meta‐analysis, fMRI, right hemisphere, semantic cognition, semantic control, theory of mind

## Abstract

Language processing has been traditionally associated with a network of fronto‐parietal and temporal regions in the left hemisphere. Nevertheless, the ‘right language network’ (frontal, temporal and parietal regions homologous to the left language network) and the ‘multiple‐demand network’ (MDN) are often involved in verbal semantic processing as well; however their role remains poorly understood. This is in part due to the inconsistent engagement of these latter two networks across linguistic tasks. To explore the factors driving the recruitment of the right language network and MDN during verbal semantic processing, we conducted a large‐scale Activation Likelihood Estimation meta‐analysis of neuroimaging studies. We examined whether the right language network is influenced by verbal stimulus type (sentences/narratives versus single words/word pairs) and whether this may be due to differences in semantic control demands and/or the presence of social content in the stimuli. Additionally, we investigated whether MDN recruitment depends on external task demands rather than semantic control demands. Our main findings revealed greater engagement of the right language network during the semantic processing of sentence/narrative stimuli, with distinct regions reflecting different functions: increased semantic control demands recruit the right inferior frontal gyrus. Instead, social content processing during a semantic task engages the right anterior temporal lobe, as well as the right posterior middle temporal gyrus. Finally, semantic processing engages the MDN, but only when external task (rather than semantic) demands increase.

## Graphical abstract

In this large‐scale meta‐analysis, we have shown that different right hemispheric areas support different semantic functions. While the right IFG seems to support semantic control, and engage when semantic demands increase, the right ATL and pMTG seem to support social processing during language.

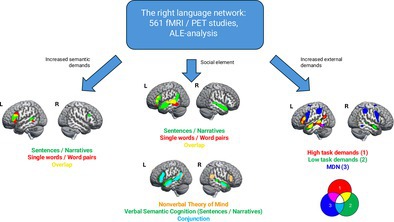

## 1 Introduction

Language processing has been traditionally associated with a network of fronto‐parietal and temporal regions in the left hemisphere (*henceforth* ‘left language network’) (Fedorenko et al. 2024; Friederici and Gierhan 2013; Price 2012). Yet, there is increasing evidence that right homologue brain regions (*henceforth* ‘right language network’) support language and verbal semantic processing as well (Lindell 2006). Not only do healthy individuals recruit right frontal, parietal and temporal brain regions during verbal semantic tasks (e.g., Branzi, Humphreys, et al. 2020; Hodgson et al. 2021); the right language network plays a crucial role in language recovery after left‐hemisphere brain damage (e.g., Crinion and Price 2005; Nardo et al. 2017). These findings align with evidence that short‐term disruption of left‐hemisphere processing by neurostimulation prompts the recruitment of the right language network to support semantic processing (Binney and Lambon Ralph 2015; Hartwigsen et al. 2013; Jung and Lambon Ralph 2016).

Although the available literature suggests that the right hemisphere possesses significant language processing capabilities, its function in language is not well understood (*see* Fedorenko et al. 2024). One main reason is that the right language network is not as consistently involved in language tasks as the left language network. For instance, the right hemisphere homologues of left hemisphere language areas seem to be rarely recruited in tasks that require single‐word or word‐pair semantic processing (Badre et al. 2005; Graessner et al. 2021; Whitney et al. 2009). Conversely, other types of tasks, such as sentence and/or narrative processing tasks, which require integration of multiple meanings, tend to recruit more extensively the right language network (Branzi, Humphreys, et al. 2020; Silbert et al. 2014; Xu et al. 2005).

These findings seem to indicate that the right language network supports meaning processing particularly during sentence or narrative processing tasks. However, the evidence remains inconclusive. The studies reviewed above differ not only in the type of semantic stimuli, but also in terms of sample sizes, task demands and analyses employed. These idiosyncrasies make it difficult to draw definitive conclusions. Therefore, the first aim of this study is to establish whether the right language network is preferentially engaged during verbal semantic processing of sentences/narratives, relative to single words/word pairs (Aim 1).

As an initial step to address this question, we adopted a meta‐analytic approach instead of conducting a single study. This method is particularly suitable for addressing this question as it allows us to establish which results are replicable and generalisable across multiple studies, while also overcoming the limitations of individual studies, which are often underpowered (Button et al. 2013) and susceptible to the effects of unique design and analytic choices.

The differential engagement of the right language network, if observed, may arise from distinct underlying processes. One possibility is that sentence/narrative tasks impose greater semantic control demands than single‐word/word‐pair tasks. The need to buffer information over time and integrate meaning across multiple timescales during sentence or narrative processing likely increases semantic control demands, leading to the recruitment of the right language network (see Branzi, Humphreys, et al. 2020; Branzi, Martin, et al. 2020; Branzi and Lambon Ralph 2023). This hypothesis is in line with evidence showing that increasing semantic control demands results in increased neural activation in the right anterior temporal lobe (ATL), right posterior middle temporal gyrus (pMTG), right inferior frontal gyrus (IFG) and right dorsal angular gyrus (AG) (Branzi, Humphreys, et al. 2020; Hodgson et al. 2023; Humphreys and Lambon Ralph 2017; Jung et al. 2021; Quillen et al. 2021; Rice et al. 2018). In healthy young individuals, the right language network is typically not recruited during language tasks, except when semantic control demands increase (Jung et al. 2021; Rice et al. 2018). However, the evidence is mixed, with studies reporting different or contrasting results (Quillen et al. 2021; Rice et al. 2018). Thus, whether the right language network supports verbal semantic processing, particularly when semantic control demands increase (e.g., Rice et al. 2018; Stefaniak et al. 2020), remains an open question. The second aim of this study is to establish whether during language tasks the engagement of the right language network varies with semantic control demands (Aim 2).

Beyond differences in semantic control demands, sentences or narratives typically convey richer social content than single‐word or word‐pair stimuli. In fact, they often depict characters with intentions, emotions or cognitive states that likely prompt the reader or listener to engage in mentalising (e.g., Branzi, Humphreys, et al. 2020; Branzi, Martin, et al. 2020; Silbert et al. 2014). They may also involve the processing of Social Cues—socially relevant stimuli that, although not necessarily requiring mentalising or explicit understanding of others' mental states, still convey information characteristic of interpersonal interactions (e.g., prosody). By contrast, single‐word or word‐pair stimuli generally lack such socially relevant information (e.g., Visser and Lambon Ralph 2011; Visser et al. 2012), unless they are explicitly designed to probe social semantics (Jung et al. 2021; Rice et al. 2018).

Thus, another non‐mutually exclusive possibility is that the right language network, or at least parts of it, does not support core semantic functions (e.g., meaning processing and retrieval) but rather, social cognition processes, which would be assimilated during the language tasks, but only when they require those specific functions.

In accord with the hypothesis that differential recruitment of the right language network could be due to social information conveyed by the stimuli, various studies have shown that social inferences about other people's mental states (or Theory of Mind—‘ToM’ processing) as compared to non‐social inferences (e.g., mechanical inference) engage brain areas within the right language network. These areas include the right ventral AG/temporoparietal junction (TPJ) and the right ATL (Saxe et al. 2004; Saxe and Wexler 2005). Along the same lines, the perception of Social Cues also engages parts of the right language network, including the right IFG, the right ventral AG/TPJ and the right pMTG (Hodgson et al. 2023; Saxe et al. 2004; Turker et al. 2023).

Furthermore, a recent large Activation Likelihood Estimation (ALE) meta‐analysis of functional neuroimaging studies has revealed some neural overlap between the ALE maps for ToM and semantic cognition in the above‐mentioned areas (Balgova et al. 2024). However, it is unclear if this is because semantic processes are integral to performing ToM tasks (Balgova et al. 2024) or rather because some stimuli (i.e., sentences/narratives) contained in the semantic cognition meta‐analysis indeed engaged some ToM and social cognition processing. Therefore, the third aim of this study is to clarify whether the right language network is specifically recruited for semantic tasks with social stimuli (e.g., sentences or narratives featuring human characters), and whether this recruitment engages the same regions as ToM and the processing of Social Cues (Aim 3).

In addition to the ‘semantic control network’ (Jefferies 2013; Lambon Ralph et al. 2017)—including brain regions such as the IFG, pMTG and dorsal AG, which are particularly engaged when semantic control demands increase (e.g., Jackson 2021; Hodgson et al. 2021)—other right‐hemisphere regions also appear to be recruited when semantic processing becomes more challenging. These brain regions, comprising bilateral frontal, parietal, medial prefrontal and posterolateral inferior temporal areas, border with the semantic areas mentioned above and refer to the ‘multiple‐demand network’ or ‘MDN’ (e.g., Duncan 2010; Fedorenko et al. 2013). The MDN is considered a functionally distinct network from the semantic control network. This is because, differently from the semantic control network, the MDN gets activated during a diverse range of executively demanding language and non‐language tasks, with stronger responses during more difficult conditions or tasks (Duncan 2010; Fedorenko et al. 2013).

In contrast with the proposed function of the semantic control network, many researchers have attributed the role of the MDN to domain‐general control rather than semantic‐specific processes (see Fedorenko et al. 2024). Accordingly, various functional magnetic resonance imaging (fMRI) studies suggest that the MDN does not support core language functions, such as perceiving word forms or accessing word meanings (e.g., Blank and Fedorenko 2017; Branzi and Lambon Ralph 2023).

Nevertheless, two important aspects need to be considered. First, these conclusions have been mainly drawn from studies that have employed low demand tasks, that is, passive tasks such as reading and listening to sentences. Thus, it is necessary to determine whether these results generalise also to tasks that require a linguistic decision to be made (e.g., whether a stimulus is a word or pseudoword, concrete or abstract, etc.). Secondly, some studies do not support the view of strong functional differences between the semantic control network and MDN (Stefaniak et al. 2020). Rather, the MDN may support language‐specific operations including the retrieval of goal‐specific semantic information (Wang et al. 2021), syntactic processing (Gordon et al. 2002) and sentence meaning processing (Mitchell et al. 2016). Therefore, an additional goal of this study (Aim 4) is to address the role of the MDN (Duncan 2010; Fedorenko et al. 2013) in language tasks and the extent to which its recruitment depends on task or semantic control demands.

Addressing this and the other questions in the study is important for clarifying the contribution of right‐hemisphere control regions to verbal semantic processing beyond the core language network. Critically, to date it remains unknown whether right‐hemisphere homologues of left‐hemisphere language regions perform similar, though weaker, language computations as their dominant left counterparts (Stefaniak et al. 2020), or whether they instead support distinct cognitive functions, such as social cognition. Understanding the contribution of the right hemisphere to verbal semantic processing is important not only to delineate the functional organisation of the language network in healthy individuals, but also to understand language recovery following brain damage in neurological patients. Accordingly, the present study addresses these targeted questions (Aims 1 to 4) through the largest ALE meta‐analysis of functional neuroimaging semantic studies in healthy individuals to date (*n* = 561).

Based on previous evidence (e.g., Branzi, Humphreys, et al. 2020; Silbert et al. 2014; Xu et al. 2005), we expect semantic tasks using sentences/narratives to recruit the right language network more extensively than those involving single words/word pairs. This is because we hypothesise that the right language network supports the same lexical–semantic functions as the left hemisphere, but is recruited specifically when semantic demands increase (Stefaniak et al. 2020). If true, then upregulation of activity within the right language network should only be observed when semantic control demands increase, across both stimulus types (single words/word pairs and sentences/narratives). However, based on previous studies (e.g., Branzi, Humphreys, et al. 2020; Silbert et al. 2014; Xu et al. 2005), semantic tasks using sentences/narratives should recruit the right language network more extensively than those involving single words/word pairs. This is because meaning processing is computed over time and across multiple words, therefore resulting in increased semantic control demands overall. The brain areas that we expect to be modulated by semantic control demands include the right pMTG, the right IFG and the right dorsal AG/intraparietal sulcus (IPS) (Lambon Ralph et al. 2017). Instead, we do not expect semantic control demands to extensively modulate activity within the MDN (Duncan 2010; Fedorenko et al. 2013). To test these hypotheses, we directly examined whether semantic control demands modulated the recruitment of the right language network in both sentences/narratives and single‐word/word‐pair tasks, separately. In fact, directly manipulating semantic control demands within each stimulus domain (sentences/narratives versus single words/word pairs) allows us to disentangle the effects of semantic control from those related to the complexity of the stimulus type. For instance, additional demands such as buffering for extended meaning integration or processing sentence‐level structure would occur in both easy and hard sentence/narrative conditions and would therefore be subtracted out. Therefore, this approach enables the identification of the neural effects of semantic control independent of stimulus complexity. To verify if the brain areas involved in semantic control overlapped with the MDN we examined the spatial overlap between the clusters identified in the above analyses and the MDN mask derived from Fedorenko et al. (2013).

Another hypothesis is that some brain regions within the right language network reflect social cognition during language tasks. Particularly, the right ATL and the right ventral AG/TPJ are often implicated in social cognition and ToM (Diveica et al. 2021; Olson et al. 2007; Saxe et al. 2004; Zahn et al. 2007). Accordingly, neural activity in these brain regions is expected to occur only when the language task involves processing stimuli with social content, that is, information about people and *'*social words that convey information about our relationships with people and can inform our understanding of their actions’ (*see* Pexman et al. 2023). Therefore, to establish whether the brain regions activated during language tasks reflect social cognition, we examined the neural overlap between a contrast reflecting verbal semantic cognition in sentence/narrative tasks with social elements that may prompt mentalising, and a contrast reflecting ToM measured during nonverbal tasks. We reasoned that any neural overlap between these two contrasts would reflect social processing and specifically ToM, rather than similarities in stimulus type (e.g., verbal input).

We also examined the neural overlap between a contrast reflecting semantic cognition measured during sentence or narrative tasks with social elements, and a contrast reflecting the processing of Social Cues. This analysis was conducted to determine whether the right hemisphere activity observed in both verbal semantic and nonverbal ToM tasks reflected ToM processing, or instead the passive detection of socially relevant information without mentalisation (i.e., Social Cues). In the present study, we focussed on Social Cues such as prosody, biological motion and processing of faces and body parts. In fact, previous studies have shown that perception of these stimuli engages right hemispheric brain regions similar to those recruited during ToM processing (Hodgson et al. 2023). In detail, these brain regions include the right IFG which responds to changes in prosody (Turker et al. 2023), the right pMTG and lateral occipitotemporal cortex which respond to visual detection of biological bodies and body parts (Peelen and Downing 2007) and finally, the right superior temporal sulcus/TPJ which responds to detection of faces (Hoffman and Haxby 2000; O'Toole et al. 2002), bodies, mouth and eye movements and finally, prosody (Calder and Young 2005; Haxby et al. 2000; Hodgson et al. 2023; Hoffman and Haxby 2000; Phillips et al. 1997; Puce et al. 1998; Taylor et al. 2007; Turker et al. 2023). Critically, for both formal conjunction analyses we expected neural overlap in the right ventral AG/TPJ, the right ATL, and the right pMTG, that is, brain regions previously associated with ToM and social cognition (Diveica et al. 2021; Hodgson et al. 2023; Saxe and Wexler 2005), during the processing of sentences and/or narratives with social content, but not when processing verbal stimuli without social content.

Finally, we hypothesised that other brain regions might be recruited during language tasks. These brain regions may overlap with the MDN (Duncan 2010). Even though in our analyses we only included semantic contrasts using active baselines or control tasks (see Methods), not all the active baselines/control tasks are necessarily designed to control for task demands. Thus, it is still possible that the recruitment of these brain regions is due to increased demands imposed by the task at hand, rather than control imposed at the lexical and semantic levels. For instance, tasks that involve deciding between different response options may require inhibitory control processes that are not required during more passive tasks such as reading or listening, with no explicit instruction to make a semantic decision. If true, then activity in brain areas overlapping with the MDN should be observed in tasks that involve increased executive control demands (e.g., tasks requiring a semantic decision), irrespective of the type of stimuli (sentences/narratives or single words/word pairs), but not during more passive tasks such as simple reading or listening.

## 2 Materials and Methods

**FIGURE 1:**
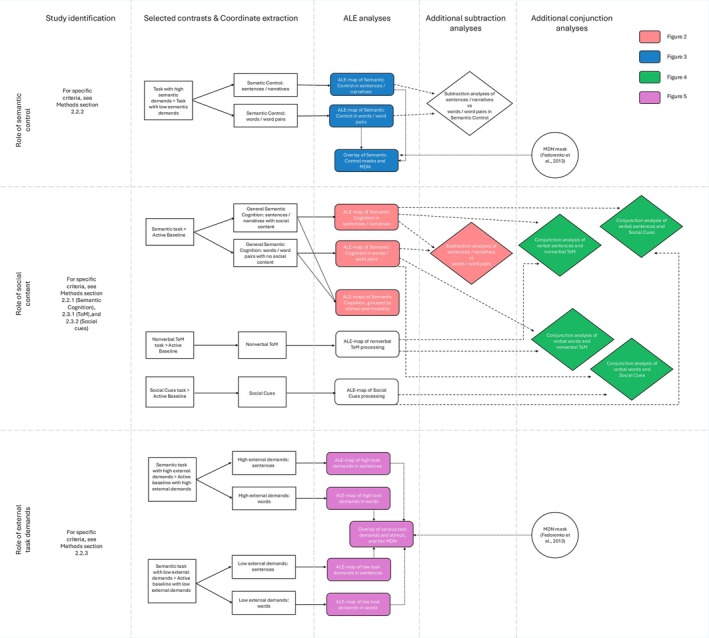
The flowchart summarises the procedures for studies identification, coordinates extraction, and key statistical analyses relative to our research questions; the analysis steps are mapped to corresponding Figures via different colour codes.

There were several steps to address the questions of the present study (see also flowchart in Figures 1 and S1). First, two ALE meta‐analyses were conducted to identify brain regions associated with general verbal semantic cognition: one for tasks involving multi‐item processing (e.g., sentences and narratives) and another one for tasks involving single‐word or word‐pair stimuli. Then, we computed two ALE meta‐analyses to examine which brain regions were sensitive to increased semantic control demands, and whether these regions differed depending on the type of stimuli, that is, sentences/narratives and single‐word/word‐pair stimuli. Then, to verify if the brain areas involved in semantic control overlapped with the MDN, we examined the spatial overlap between the clusters identified in the above analyses with the MDN (Fedorenko et al. 2013).

In a second step, two ALE meta‐analyses were conducted to reveal the brain regions reflecting social cognition processing, including nonverbal ToM and the processing of Social Cues (e.g., prosody and biological motion). Then, through formal conjunction analysis, we examined whether the brain regions sensitive to multi‐item semantic processing (sentences/narratives) (see above) were also sensitive to social cognition processing measured in nonverbal ToM and Social Cues processing.

In a third step, we examined whether the neural network supporting verbal semantic cognition in sentences/narratives and single words/word pairs was modulated by external task demands. To do so, we conducted separate ALE meta‐analyses and identified the neural networks underlying verbal semantic cognition across tasks with low and high control demands. Finally, we examined the extent to which these regions overlapped with the MDN (Duncan 2010; Fedorenko et al. 2013).

### 2.1 Studies Selection

All ALE meta‐analyses were based on experimental studies published in peer‐reviewed journals in English, describing task‐based activation coordinates reported in Montreal Neurological Institute (MNI) or Talairach coordinate systems, as a result of univariate analyses from fMRI or Positron Emission Tomography (PET) studies, and studying only young (< 40 years old) healthy adults.

As in previous studies (Diveica et al. 2021; Hodgson et al. 2023; Jackson 2021), coordinates from different contrasts within the same study and participant group were combined when entered into the meta‐analysis (Müller, Cieslik, et al. 2018), except when the contrasts differed in a crucial aspect examined in the study. For instance, contrasts differing in dimensions such as ‘stimulus modality’ (auditory/visual) or ‘stimulus type’ (single words or word pairs/sentences or narratives) were entered separately. Other contrasts, instead, were merged because they tapped into processes not relevant to our study. For example, contrasts differing in ‘word regularity’ (e.g., reading regular words versus pseudowords and reading irregular words versus pseudowords) were pooled together—as long as they were performed on the same group of participants—as differences between regular and irregular words were not relevant for the aims of the present study. Additionally, the pooling of data is recommended because the results of these separate contrasts would not be independent, given they originate from the same sample of subjects, and therefore might negatively affect the validity of the meta‐analysis (Müller, Cieslik, et al. 2018).

For all analyses, coordinates were obtained from previous meta‐analyses (see details below). Additionally, the Web of Science (www.webofscience.com) was used to identify new studies for each meta‐analysis, employing the same search terms as those used in the original meta‐analyses (see details below). In line with previous meta‐analyses (Diveica et al. 2021; Hodgson et al. 2023; Jackson 2021; Turker et al. 2023), we only included coordinates derived from studies using whole‐brain analyses and discarded coordinates derived from studies focussing on region‐of‐interest analyses or small‐volume correction analyses since these types of analyses violate a key assumption of coordinate‐based meta‐analyses (Eickhoff et al. 2012; Müller, Cieslik, et al. 2018).

While the coordinates for each ALE meta‐analysis and the search for new studies were based on results and selection criteria of existing meta‐analysis studies, we adjusted the list of studies (and coordinates) to make the different meta‐analyses optimally comparable to each other (see Tables S1–S4). In detail, for the general verbal semantic cognition, verbal semantic control, and social cognition ALE meta‐analyses, we only included studies in which the experimental condition was contrasted against an active baseline. Instead, we did not include coordinates taken from studies where the experimental condition of interest was contrasted against the rest/passive baseline which required lower cognitive demands than the experimental condition. This approach is beneficial not only for controlling methodological differences across various types of stimuli (sentences/narratives versus single words/word pairs) or cognitive domains (semantic versus social), but also for ensuring that the observed neural networks reflect the processes of interest rather than task‐related differences between experimental conditions and baseline. Many brain regions associated with semantic cognition and social cognition overlap with the Default Mode Network (Andrews‐Hanna et al. 2010; Buckner et al. 2008; Raichle et al. 2001). The Default Mode Network is a resting‐state network that typically shows reduced activity during demanding tasks, but also increased activity during tasks requiring internally oriented cognition (Konu et al. 2020; Smallwood et al. 2021; Vatansever et al. 2017). Therefore, a baseline that does not match the experimental condition in terms of difficulty and the degree of internally versus externally oriented processing can introduce confounding for the interpretation of meta‐analytical results. The total number and the type of studies for each meta‐analysis are listed in Table 1.

**TABLE 1:** Number of studies, coordinates and subjects included in each ALE meta‐analysis.

General verbal semantic cognition			
Stimuli, perceptual modalities and task types	Experimental studies	Coordinates	Subjects
Sentences/narratives	98	1684	3479
Low task demands	56	973	2345
Auditory	27	476	866
Choice	2	23	36
Comprehension	23	390	784
Production	2	63	46
Visual	29	497	1479
Choice	2	59	44
Comprehension	26	437	1416
Production	1	1	19
High task demands	42	711	1134
Auditory	19	263	378
Choice	18	246	360
Comprehension	—	—	—
Production	1	17	18
Visual	22	437	746
Choice	21	407	727
Comprehension	—	—	—
Production	2	30	44
Visual and auditory	1	11	10
Choice	1	11	10
Comprehension	—	—	—
Production	—	—	—

Single words/word pairs	120	2035	3272
Low task demands	30	533	776
Auditory	8	134	229
Choice	4	61	126
Comprehension	3	69	87
Production	1	4	16
Visual	21	387	518
Choice	4	28	96
Comprehension	14	315	362
Production	3	44	60
Visual and auditory	1	12	29
Choice	1	12	29
Comprehension	—	—	—
Production	—	—	—
High task demands	90	1502	2496
Auditory	19	268	527
Choice	17	254	503
Comprehension	—	—	—
Production	2	14	24
Visual	68	1181	1903
Choice	60	1074	1612
Comprehension	—	—	—
Production	8	107	291
Visual and auditory	3	53	66
Choice	3	53	66
Comprehension	—	—	—
Production	—	—	—
Total general verbal semantic cognition	218	3719	6754

Verbal semantic control			
Sentences/narratives	51	488	1596
Auditory	18	110	443
Choice	8	53	215
Comprehension	10	57	228
Production	—	—	—
Visual	32	335	1113
Choice	17	150	516
Comprehension	13	159	570
Production	2	27	27
Visual and auditory	1	43	40
Choice	—	—	—
Comprehension	—	—	—
Production	1	43	40

Single words/word pairs	44	642	1467
Auditory	3	89	109
Choice	2	87	98
Comprehension	—	—	—
Production	1	2	11
Visual	41	553	1358
Choice	25	283	802
Comprehension	0	0	0
Production	16	270	556
Total verbal semantic control	95	1130	3063

Nonverbal Theory of Mind (ToM)			
Visual	98	2078	3163
Choice	89	1938	2970
False belief	7	126	122
Intention	4	64	86
Mental state	66	1491	2508
Predict behaviour	12	260	254
Comprehension	9	140	193
Mental state	9	140	193
Total nonverbal ToM	98	2078	3163

Social Cues			
Visual	111	841	2204
Face versus non‐face perception	47	247	1072
Biological versus non‐biological motion perception	64	594	1098
Auditory	39	740	760
Prosody processing	39	740	760
Total Social Cues	150	1581	2964

### 2.2 Verbal Semantic Cognition

The experiments assessing verbal semantic cognition have employed many different tasks and designs. These often vary substantially in terms of cognitive load, computations and modalities involved. For instance, in some tasks, both the experimental task and control task might require participants to passively comprehend a word or sentence, while in other tasks, participants might be required to generate several words according to instructions. These differences may affect the recruitment of language areas as well as the MDN (Diachek et al. 2020; Hu et al. 2023) and result in an artificial divergence in the neural networks supporting semantic cognition during sentence/narrative processing versus single‐word/word‐pair processing. Therefore, to ensure that studies included in each semantic ALE meta‐analysis did not differ substantially in terms of cognitive load, computations and modalities involved, that is, aspects that may affect the results, we screened and classified semantic studies according to stimulus modality (visual, auditory or audio‐visual) and task type. Task types were as follows: performing a choice (requiring a decision between two or more options, usually accompanied by a button press), passive comprehension (reading or listening) and cue‐based production (both external: semantic fluency tasks and internal: for example, ‘think about an item belonging to a semantic category’). We also categorised studies according to task demands, in line with Stefaniak et al. (2021) (see Table 1 and Section 2.2.3 for details) to address the role of the MDN (Duncan 2010; Fedorenko et al. 2013) in verbal semantic processing and particularly the extent to which its recruitment depends on external task demands.

#### 2.2.1 General Verbal Semantic Cognition in Words Versus Sentences

These ALE meta‐analyses were based on a selection of studies reported in Jackson (2021) measuring the neural basis of verbal semantic cognition through the comparison of neural activity during verbal semantic tasks against non‐semantic (or less semantic) tasks (active baseline). Thus, none of the tasks included in the verbal semantic cognition ALE meta‐analyses explicitly required social cognition and/or ToM processing. For example, studies containing tasks where participants were asked about a character's emotions, intentions, perspective or mental state (e.g., ‘He suggested she should stay longer. Was he sad to see her leave?’), contrasted against physical inference baseline tasks (‘The cabin was built in the woods in 1900. In 1950, all trees surrounding it were cut down. On a photograph taken in 1930, do trees surround the cabin?’) were excluded. Similarly, studies in which participants were presented with adjectives, and asked whether they described themselves or someone else, or studies asking participants to determine whether the name of a presented individual was referring to a famous person or not, were excluded. Therefore, 156 studies met the criteria, yielding 2367 coordinates.

Examples of contrasts included in the verbal semantic ALE meta‐analyses refer to comparisons between processing words versus pseudowords, generating words that matched the probe based on semantic association versus rhyme (phonology) and listening to meaningful sentences versus scrambled/non‐meaningful sentences. We also updated the list of studies using the same search terms as those used by Jackson (2021), that is, [‘semantic’, ‘comprehension’ or ‘conceptual knowledge’], combined with [‘PET or fMRI’], excluding [‘patient’, ‘priming’, ‘disorder’, ‘dementia’, ‘ageing’, ‘bilingual’, ‘meta‐analysis’ or ‘multivariate’]. We included studies published between 19/06/2019 and 05/01/2025. Therefore, 58 additional studies with 1345 coordinates were added to the studies sourced from Jackson (2021).

To identify the semantic neural network during sentence versus word processing, we separated experiments assessing semantics according to whether the semantic task included sentences/narratives or single words/word pairs. To determine whether meaning processing activates neural regions similar to those involved in nonverbal ToM and Social Cue processing, specifically when stimuli contain social elements, we examined which stimuli were imbued with social content and which were not. Stimuli with a social element were defined as words or sentences describing people's relationships, people's mental states, and words or sentences that could inform our understanding of people's actions and intentions (Pexman et al. 2023)—but only when they referred to specific people or characters within the stimuli.

To ensure we adhered to this definition of socialness, stimulus lists were consulted. For studies where the stimuli were not publicly available, we contacted the authors directly and only included those for which we could confirm that the stimuli met the criteria outlined above. Additionally, to determine whether stimuli were characterised by social content, we applied the following annotation rule: Studies that employed stimuli describing a character's actions, intentions, mental states or interpersonal relationships were categorised as having social content. Examples include sentences describing (1) actions of characters (e.g., ‘The boy counted the ducks’), (2) emotional states (e.g., ‘He was annoyed that persisting problems waste a lot of time’), (3) interpersonal interactions, such as dialogs (e.g., ‘May I take a picture here?’—‘Yes, if you do not use the flash’) and (4) tasks including all of the above (e.g., processing fables or the entirety of The Little Prince audiobook).

Given the nature of this annotation rule, all studies classified as ‘social’ employed sentences or narratives as stimuli—with the exception of 10 studies that, while using such formats, did not involve human characters or social inferences and were therefore excluded from our analyses (see below). Interestingly, aside from five studies (which were then removed from the data analysis; see below), there were no studies using single words or word pairs that described interpersonal relationships (e.g., ‘mother–child’), emotional states (e.g., ‘mother‐anxious’) or social intentions (e.g., cooperation, deceit or intention attribution). Instead, the vast majority of studies employing single words or word pairs were categorised as ‘non‐social’ as they typically required semantic relatedness judgements on concrete words lacking any reference to characters, social traits or human interactions.

Importantly, among the single‐word/word‐pair studies there were some ‘borderline’ cases, that is, studies with potential social connotations. For instance, in some studies participants were asked to rate the pleasantness of words (for example, ‘holiday’). However, these studies were categorised as ‘non‐social’, as they did not involve human characters and/or require the processing of interpersonal relationships or social intentions. Other studies included tasks requiring participants to generate alternative uses for objects. These too, were categorised as ‘non‐social’, as these tasks did not involve reasoning about social actions or intentions, but rather focused on semantic processing and the retrieval of object‐specific manipulation knowledge. Additionally, studies that involved judging whether a word was concrete or abstract were also categorised as ‘non‐social’, despite some abstract words, such as ‘dignity’ or ‘fear’, possibly having a social connotation. This is because these tasks did not include descriptions of human characters or relationships which might lead participants to infer interpersonal relationships or mental states.

In summary, in the sentence/narrative ALE meta‐analyses we only included stimuli with social content, that is, sentences/narratives that involved at least one human character and information that could lead to mentalisation. Instead, in the single‐word/word‐pair ALE meta‐analyses we only included stimuli without social content.

Importantly, as anticipated above, we excluded from our data analyses studies using single‐word/word‐pair tasks that contained social elements, as well as studies using sentence/narrative tasks without social content (e.g., describing natural/physical phenomena with no characters involved). This decision was based on the small number of such studies (*n* = 5 and *n* = 10, respectively), which was insufficient to allow for meaningful comparisons between stimuli with and without social content within each stimulus type.

In total, we analysed 98 studies reporting 1684 coordinates involving sentences/narrative tasks with a social element, and 120 studies reporting 2035 coordinates involving single‐word/word‐pair tasks with no social element. We also determined the type of task and modality for each task. The full list of studies is reported in Table S1, with a brief description of each study's tasks, stimuli and contrast types (see column ‘Task summary & Contrast’).

Finally, to ensure that any observed differences between semantic processing of sentences/narratives and single words/word pairs were not influenced by the type of task (choice, comprehension, production), we conducted the same analyses as above (verbal semantic cognition in sentences/narratives and in single words/word pairs) on a subset of studies, focusing only on tasks that required participants to make a choice. This control analysis accounted for differences in task types (and the likely cognitive demands) that varied substantially between sentence/narrative tasks and single‐word/word‐pair tasks. Furthermore, to minimise potential confounding effects related to the stimulus modality, we created this subset so that within both stimulus types (sentences/narratives and single words/word pairs) there were almost equal numbers of studies (and coordinates) involving auditory versus visual modalities. In detail, the sentence/narrative subset of choice tasks comprised 20 auditory studies with 268 coordinates and 19 visual studies with 253 coordinates. Instead, the single‐word/word‐pair subset of choice tasks comprised 21 auditory studies with 325 coordinates and 20 visual studies with 336 coordinates.

#### 2.2.2 Verbal Semantic Control (Hard Versus Easy Semantic Task/Condition) in Words Versus Sentences

These ALE meta‐analyses included studies that manipulated the amount of verbal semantic control required during the task and directly contrasted a harder verbal semantic condition against an easier verbal semantic condition. Coordinates were partly derived from Jackson (2021), including only studies with verbal stimuli (80 studies, 874 coordinates). We then searched the literature for new studies published between 19/06/2019 and 01/05/2025. The inclusion and exclusion criteria were based on Jackson (2021), as were the search words: [‘semantic’, ‘comprehension’ or ‘conceptual knowledge’], combined with [‘PET’ or ‘fMRI’], combined with [‘selection’, ‘retrieval’, ‘inhibition’, ‘control’, ‘elaboration’, ‘fluency’, ‘ambiguity’, ‘metaphor’ or ‘idiom’], excluding [‘patient’, ‘priming’, ‘disorder’, ‘dementia’, ‘ageing’, ‘bilingual’, ‘meta‐analysis’ or ‘multivariate’]. In short, we included studies that manipulated the difficulty of semantic tasks and, therefore, the cognitive load on semantic control by either (1) focusing on a meaning that is less frequently used, for example, requiring a weaker association or subordinate homonyms; (2) increasing the number of competitors or distractors when an answer is required, or decreasing the semantic distance between target and distractors; (3) requiring processing a semantic violation or subordinate homonyms; (4) increasing the unpredictability or surprisal factor of semantic elements by not giving enough context; or (5) requiring flexible switching between different meanings or instructions (Jackson 2021). This search yielded 17 more studies, reporting 265 coordinates. To determine whether some brain areas could be differentially modulated by increased semantic control demands depending on the type of stimuli, we split this dataset similarly as above, according to whether the stimuli corresponded to sentences/narratives or single words/word pairs (see Table S2; a brief description of each study's tasks, stimuli and contrast types can be found in the ‘Task summary & Contrast’ column).

#### 2.2.3 General Verbal Semantic Cognition and Task Demands (High Versus Low Demands)

As mentioned above, within the general verbal semantic cognition ALE meta‐analysis, we separated studies according to task difficulty. In short, all types of tasks (choice, comprehension and production) were categorised as ‘high task demands’ if they required the participant to perform a semantic decision or production of more than one word. Examples of such tasks included differentiating between words and pseudowords, making semantic relatedness judgements and producing words or thinking about them according to criteria based on meaning or grammar. A task was categorised as ‘low task demands’ if there was no linguistic decision to perform, or it required a very simple identity match or a low‐level perceptual decision. Passive comprehension tasks fell into this category, as did low‐level/perceptual choice and matching tasks in which the experimental task was not semantic in nature (e.g., determining whether two items were identical or not), as well as production tasks with single item naming or repetition. In total, we examined 86 studies yielding 1506 coordinates that were categorised as ‘low task demands’, and 132 studies yielding 2213 coordinates that fell into the ‘high task demands’ category. The full list of studies is reported in Table S1. The classification of low versus high (external) task demands for each study is provided in the column titled ‘External Task Demands’. For brief descriptions of the tasks and stimuli used in each study, refer to the column ‘Task summary & Contrast’.

### 2.3 Social Cognition

We examined verbal semantic tasks separately according to stimuli with versus without social elements (sentences/narratives versus single words/word pairs). However, the comparison between sentence/narrative tasks versus single‐word/word‐pair tasks may have resulted in brain activations reflecting non‐social factors, such as syntax processing or an increased cognitive load on working memory and attention. Therefore, to establish which brain regions were active during semantic processing of sentences/narratives and reflected the processing of social elements (e.g., identifying a character within the narrative and performing mentalisations based on the characters' actions and intentions, etc.), we examined the similarities between brain areas active during narrative/sentence semantic tasks and different types of social cognition tasks including nonverbal mentalisation and the processing of Social Cues. To do so, we computed two separate ALE meta‐analyses, one reflecting nonverbal ToM and the other reflecting Social Cue processing. The list of studies contributing to social cognition contrasts is reported in Tables S3 and S4.

#### 2.3.1 Nonverbal ToM

The ALE meta‐analysis for nonverbal ToM was partly sourced from Diveica et al. (2021), discarding studies with verbal stimuli. All the categories defined in their ALE meta‐analysis were included in the present study (i.e., ToM, Trait inference, Empathy and Moral reasoning). The tasks required participants to perform inferences about a character's intents, traits or emotional state via nonverbal stimuli. For example, some tasks presented participants with a cartoon story and required them to select the final image based on the intentions of the cartoon characters. Other tasks involved observing facial expressions to determine the emotion being displayed. These two tasks were categorised as ‘intention’ and ‘mental state’, respectively (see Table 1). Additionally, some studies presented the false belief paradigm, in which the perspective or knowledge of the participant differs from the perspective of a character. The participant is then required to disassociate from their own perspective, adopt the character's point of view (as in the classic Sally‐Anne task; Baron‐Cohen et al. 1985), and infer the character's mental state. Finally, some experiments presented non‐human entities, such as cartoon shapes, which were moving either randomly or as if with a human goal (helpful, protective, etc.). In these tasks, participants were required to determine the type of behaviour (‘predict behaviour’) (see Table 1).

As with the verbal semantic cognition ALE meta‐analyses, we discarded studies that used a passive baseline. Therefore, coordinates reported in the current study were sourced from experiments in which the tasks were contrasted with an active baseline that matched the general cognitive demands of the experimental condition/task, but required no mentalisation (e.g., making physical inferences or perception of non‐human images). This resulted in 76 studies with 1398 coordinates. We extended this dataset with new studies, using the same search terms and criteria as described in Diveica et al. (2021). Thus, the Web of Science was searched with the key words [‘PET’ or ‘fMRI’], combined with [‘theory of mind’, ‘ToM’, ‘mentalising’, ‘mentalizing’, ‘social judgement’, ‘social evaluation’, ‘social attribution’, ‘trait inference’, ‘impression formation’, ‘empath*’, ‘morality’, ‘moral’, ‘moral decision making’, ‘moral emotion’, ‘harm’ or ‘guilt’]. We included studies published between 01/03/2020 and 05/01/2025. This resulted in 22 additional studies with 680 coordinates in addition to those sourced from Diveica et al. (2021) (76 studies with 1398 coordinates), resulting in a total of 98 studies with 2078 coordinates. The full list of studies is reported in Table S3.

#### 2.3.2 Social Cues

This ALE meta‐analysis included other types of social cognition tasks that required processing stimuli with social valence but were not explicitly ToM tasks. Therefore, in this ALE meta‐analysis, we included studies of biological motion, face perception and changes in prosody. These tasks presented participants with cues (e.g., prosody) they would observe in a social interaction, without directly requiring them to perform ToM or a semantic task. The studies were partly sourced from Hodgson et al. (2023), particularly the category labelled ‘biological motion’. To avoid examining the effects of face/emotion processing and mentalising along with biological motion processing, we only included studies in which the actors' faces were not visible (40 studies, 151 coordinates). Since Hodgson et al. (2023) only examined studies published before 2010, we searched the literature using the same criteria (see Grosbras et al. 2012), and we updated the studies by including publications from 19/05/2010 until 05/01/2025. Search terms on the Web of Science included [‘fMRI’ or ‘PET’], as well as [‘biological motion’, ‘action observation’ and ‘human movement’]. As mentioned above, we excluded studies where stimuli included faces that were visible. This yielded 20 studies with 383 coordinates.

We then assessed ‘face perception’ and included 54 studies (158 coordinates) from the ALE meta‐analysis of Hodgson et al. (2023). Studies examining the perception of emotions (*n* = 13) were excluded from this ALE meta‐analysis. In fact, processing emotional faces and making inferences about them were classified as ToM. Thus, these studies were included in the ALE meta‐analysis of nonverbal ToM instead (see above). We then updated the dataset following the same search terms and criteria as in Hodgson et al. (2023) (see also Müller, Höhner, and Eickhoff 2018). Thus, the search terms included: [‘fMRI’, ‘PET’, ‘face’, ‘facial’, ‘emotion’, ‘expression’, ‘neutral’, ‘localizer’]. We selected studies published between 01/01/2016 and 05/01/2025 which contrasted images or videos of faces with a non‐face or scrambled counterpart. Instead, we discarded studies that contrasted assessing emotional versus neutral faces. We did so to avoid including activation coordinates during emotional processing, as tasks containing emotional processing would be already included in our ToM contrast. This new search yielded six new studies with 135 coordinates.

Finally, we included studies assessing auditory prosody processing. Our dataset was based on the prosody ALE meta‐analysis recently published by Turker et al. (2023). These were tasks including judging whether sentences had the same melody/intonation, whether two auditorily presented words differed in their pitch or passive listening of auditory material in the form of meaningless sound envelopes. In total, 38 studies (677 coordinates) were selected from this ALE meta‐analysis. We then performed a search with the terms [‘functional magnetic resonance imaging’, ‘positron emission tomography’ and ‘prosody’], inlcuding studies published between 01/04/2021 and 05/01/2025. This new search resulted in two additional studies with 63 coordinates. The full list of studies is reported in Table S4.

### 2.4 Data Analysis

All coordinate‐based ALE meta‐analyses were performed via the revised ALE algorithm (Eickhoff et al. 2009, 2012; Turkeltaub et al. 2012), using GingerALE version 3.0.2 (http://brainmap.org/ale). All analyses were performed in the MNI152 space. Before running the analyses, all coordinates reported in Talairach space were converted to MNI using the Lancaster transform (‘icbm2tal’) via GingerALE (Laird et al. 2010; Lancaster et al. 2007). GingerALE treats each activation focus as a spatial probability distribution, centred at the given coordinate. It then creates a map in which each voxel of the brain is given a value that signifies the probability of one of these distributions falling within the voxel, resulting in the ALE value (Turkeltaub et al. 2002). Permutation processes ensure differentiating between noise and true convergence of foci, and the process accounts for the spatial uncertainty caused by the between‐subjects and inter‐laboratory nature of meta‐analytic approaches (Eickhoff et al. 2009). Importantly, following the recommendation by Müller, Cieslik, et al. (2018), we only computed ALE meta‐analyses in which the number of included experiments exceeded the recommended 20.

For each ALE meta‐analysis, we obtained an ALE‐map which was thresholded using an *a priori* cluster‐forming threshold of *p* < 0.001 (uncorrected), followed by a conservative cluster‐level family‐wise error (FWE) correction with a *p*‐value of 0.001, with 10,000 permutations. As explained in Eickhoff et al. (2012), this method creates a random set of foci with the same number of experiments and subjects as the provided dataset, repeated 10,000 times and assesses for each found cluster how likely it is to obtain a cluster of the same size from these randomly relocated foci.

We then performed subtraction and conjunction analyses to reveal differences and similarities between the obtained ALE maps. Contrast images were generated by subtracting one ALE map from another ALE map. The conjunction images were generated using the voxel‐wise minimum value (Nichols et al. 2005) of the included ALE maps to highlight shared activation. For both analyses, we used a priori cluster‐forming threshold of *p* < 0.001 (uncorrected), with a minimum cluster volume of 200 mm^3^, which was estimated using a permutation approach with 10,000 repetitions, as in Diveica et al. (2021). This algorithm pools both datasets and randomly divides the experiments in line with the original numbers in each group. ALE maps are then assigned for these two newly formed sets of coordinates and the difference of ALE values between the two sets is calculated for each voxel in the brain. With 10,000 permutations, a null distribution is created, against which the differences in ALE values between the original groups of observed data are compared (Eickhoff et al. 2011). Since there is still no established method to correct ALE difference maps for multiple comparisons, we set a conservative significance threshold of *p* < 0.001, as conventional (Eickhoff et al. 2011). Thus, we reported only clusters that survived the threshold of *p* < 0.001 and the cluster‐size threshold of 200 mm^3^.

All coordinates are reported in the MNI space. For identifying clusters, the software package Statistical Parametric Mapping (SPM12) was used, with the software Matlab 2023b. Within SPM12, we utilised the Anatomy Toolbox version 3 (Eickhoff et al. 2005, 2006, 2007). For each local maxima, we reported the most likely cytoarchitectonic area, via the Cytoarchitecture/Julich‐Brain Atlas feature (Zilles et al. 2002; Zilles and Amunts 2010), for increased accuracy. The cytoarchitectonic information for each one of the foci was assigned based on the Maximum Probability Map, along with the quantified probability for the most likely histological area found at this location. To identify the labels for coordinates reported in the Results, we used the Harvard‐Oxford microanatomical atlas, also via the Anatomy Toolbox (SPM).

#### 2.4.1 General Verbal Semantic Cognition—Words Versus Sentences

First, we performed individual ALE meta‐analyses with single‐word/word‐pair studies and sentence/narrative studies versus their respective non‐semantic (or less semantic) baselines. This allowed us to identify the brain regions involved in the semantic processing of these different stimulus types. To examine the brain regions that were significantly different between these two stimulus types, we performed a subtraction analysis. To provide a more comprehensive overview of the general verbal semantic network, we also conducted another ALE meta‐analysis including both sentences/narratives and single words/word pairs, which is reported in Figure S2A.

#### 2.4.2 Verbal Semantic Control—Words Versus Sentences

To reveal the brain regions involved in verbal semantic control processes and how they might differ based on stimulus type, we performed individual ALE meta‐analyses measuring semantic control with single words/word pairs on one side, and sentences/narratives on the other. Additionally, to identify the brain regions specific to one type of stimulus, we performed a subtraction analysis between ALE meta‐analyses measuring semantic control during sentence/narrative tasks and during single‐word/word‐pair tasks. To provide a more comprehensive overview of the verbal semantic control network, we also conducted another ALE meta‐analysis including both sentences/narratives and single words/word pairs, which is reported in Figure S3.

#### 2.4.3 Social Cognition and Its Neural Overlap With General Verbal Semantic Cognition

To determine whether the right language network activated during verbal semantic tasks involving sentences and narratives (as opposed to single words or word pairs) reflects the processing of social content, we first conducted two ALE meta‐analyses: one to identify the brain regions active during nonverbal ToM and another to pinpoint the brain regions involved in processing verbal and nonverbal Social Cues, which would accompany social interactions, but do not require direct mentalisation. Then, we performed one conjunction analysis to identify the brain areas reliably implicated in both verbal semantic processing of sentences/narratives (containing social elements) and nonverbal ToM processing. With this, we aimed to identify clusters active during social inference, independent of modality, both when instruction to mentalise were present (nonverbal ToM tasks) and when they were absent (verbal tasks with sentences/narratives with social elements). We also performed a subtraction analysis to determine whether any brain regions were significantly more activated during nonverbal ToM processing than during sentence/narrative processing, and vice versa. The results of this analysis are reported in Figure S4.

Furthermore, we also explored the overlap between verbal semantic cognition and the processing of nonverbal and verbal Social Cues. Thus, we conducted an additional conjunction analysis between semantic processing in sentence/narrative tasks and Social Cue processing measured in verbal and nonverbal tasks. Finally, to ensure the overlap observed with the sentence/narrative tasks was due to the social content of the stimuli rather than verbal semantic processing (which is likely to be engaged, at least to some extent, in both semantic and social cognition ALE meta‐analyses), we performed the same conjunction analyses between social cognition tasks and verbal semantic tasks, but we focused only on single words/word pairs (without social content).

#### 2.4.4 General Verbal Semantic Cognition and Task Demands (High Versus Low Demands)

To isolate areas responsive to high versus low task demands during verbal semantic processing, we sorted the coordinates of the general verbal semantic cognition ALE meta‐analysis according to task demands. Then we performed separate ALE meta‐analyses for low and high demand tasks. To pinpoint whether the MDN is particularly activated because of task demands (e.g., tasks requiring a semantic decision), we examined the overlap between the individual ALE meta‐analyses (high and low demand tasks) and the MDN mask identified by Fedorenko et al. (2013).

## 3 Results

The full list of activation clusters and local maxima foci is reported in Tables [Link], [Link].

### 3.1 General Verbal Semantic Cognition (Aim 1)

#### 3.1.1 Words Versus Sentences—Separate ALE Meta‐Analyses

**FIGURE 2:**
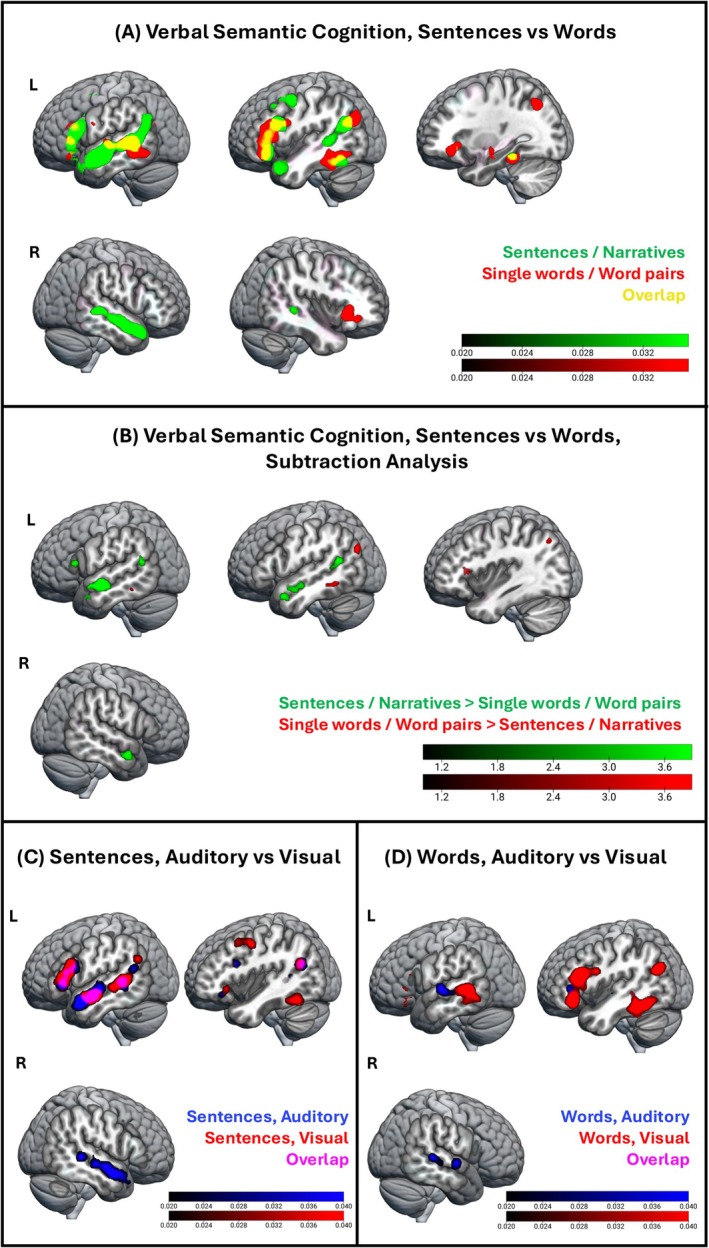
The neural network of general verbal semantic cognition split by stimuli type and modality. (A) Brain regions reliably activated during verbal semantic cognition (semantic versus non‐semantic or less semantic tasks) split by stimulus type (red: single words/word pairs; green: sentences/narratives), overlaid. Colour bar: ALE‐values; cluster forming threshold: *p* < 0.001 (uncorrected); cluster extent correction: family‐wise error (FWE) *p* < 0.001. (B) Brain regions differentially engaged depending on stimulus type (red: single words/word pairs versus sentences/narratives; green: sentences/narratives versus single words/word pairs), calculated via subtraction analysis. Colour bar: Z‐scores, cluster forming threshold: *p* < 0.001 (uncorrected), minimum cluster volume: 200 mm3. (C) Brain regions reliably activated during verbal semantic cognition (semantic versus non‐semantic or less semantic tasks) for sentences/narratives split by the perceptual modality of the task, overlaid. Colour bar: ALE‐values; cluster forming threshold: *p* < 0.001 (uncorrected); cluster extent correction: FWE *p* < 0.001. (D) Brain regions reliably activated during verbal semantic cognition (semantic versus non‐semantic or less semantic tasks) for single words/word pairs split by the perceptual modality of the task, overlaid. Colour bar: ALE‐values; cluster forming threshold: *p* < 0.001 (uncorrected); cluster extent correction: FWE *p* < 0.001.

The first aim of our study was to establish whether the right language network was differently recruited depending on the type of stimuli (single words/word pairs with no social content versus sentences/narratives with social content). After completing an ALE meta‐analysis with all tasks assessing verbal semantic cognition (semantic > non‐semantic or less semantic contrast) (see Figure S2A), we examined if this neural network for verbal semantic cognition fractionated depending on the stimuli type (Figure 2A,B). We determined this via two separate ALE meta‐analyses, one with tasks involving semantic stimuli with sentences/narratives > non‐semantic (or less semantic) task and one with single words/word pairs > non‐semantic (or less semantic) task.

On the one hand, results revealed that semantic processing of sentences/narratives recruited bilaterally the middle and superior ATL, as well as the mid‐ and posterior portions of the temporal lobe, including pMTG. This activity in the right anterior and middle temporal lobes (as well as in the most anterior portions of the left ATL) was observed only in response to sentences/narratives.

Semantic processing of single‐word/word‐pair stimuli activated the right insula. In the left hemisphere, some activation in the left pMTG, as well as a more inferior portion of the left posterior temporal gyrus (pITG) was observed during single‐word/word‐pair tasks. The left dorsal and ventral IFG (Pars Triangularis and Pars Orbitalis, respectively) showed activation during semantic processing of both multi‐item and single‐word/word‐pair stimuli. Finally, the left AG fractionated depending on the type of stimuli, with sentences/narratives recruiting ventral AG/TPJ and single‐word/word‐pair stimuli activating more dorsal and posterior portions of the left AG. Activation coordinates for all contrasts involving verbal semantic cognition are reported in Table S5.

#### 3.1.2 Words Versus Sentences—Subtraction Analysis Between ALE Meta‐Analyses

To examine stimulus‐type specificity in the neural effects—namely, whether certain brain regions are not only preferentially but also uniquely involved in the semantic processing of specific types of stimuli—we conducted a direct comparison between the two separate meta‐analyses described above. This analysis is also informative because some brain areas showed overlap between the semantic processing of multi‐item and single‐word/word‐pair tasks. Therefore, by performing a subtraction analysis we could determine whether these overlapping brain regions were recruited to different extents depending on the stimuli (*see* Figure 2B). We found that the right ATL (together with the left ATL) was more strongly recruited during semantic tasks with sentences/narratives as compared to tasks with single words/word pairs. The left TPJ, as well as the left IFG Pars Triangularis also showed a significantly greater activation for semantic processing during sentences/narratives versus single words/word pairs. Semantic processing of single words/word pairs did not recruit regions of the right language network to a larger extent than the processing of sentences/narratives. In the left hemisphere, the pITG and the lateral occipital cortex seemed to be recruited more strongly during single‐word/word‐pair tasks.

#### 3.1.3 Words Versus Sentences Split by Perceptual Modality

An inspection of the semantic cognition dataset revealed an unequal distribution of perceptual modality between sentences/narratives and single‐word/word‐pair tasks (see Table 1). While sentence/narrative tasks included a similar number of auditory and visual tasks, resulting in comparable coordinate counts, single‐word/word‐pair tasks were predominantly in the visual modality, with more than three times as many studies (and coordinates) from visual tasks compared to auditory ones. Therefore, to ensure the observed differences between sentences/narratives versus single words/word pairs were not due to the differences in perceptual modality, we examined the recruitment of brain regions based on perceptual modality. We performed four individual ALE meta‐analyses, dividing the verbal semantic tasks based on both stimulus type and perceptual modality (see Figure 2C,D).

The results revealed that semantic processing of visual stimuli did not activate large clusters in the right hemisphere across both sentence and word task types. Semantic processing of auditory stimuli, instead, activated the right posterior temporal lobe in both sentences/narratives and single‐word/word‐pair tasks. This result is not fully consistent with the results reported above (i.e., single words/word pairs versus non‐semantic or less semantic tasks), which did not show any activation in the right hemisphere (see Figure 2). This discrepancy might be due to the imbalance between auditory and visual tasks in single‐word/word‐pair experiments. As these tasks were predominantly presented in the visual modality, any activation linked to the auditory properties of the stimuli may have been too subtle to be detected in the ALE meta‐analysis. However, the right ATL was consistently activated only for tasks with sentences/narratives as stimuli, indicating that neural activity in this brain area is primarily modulated by the presence of sentence/narrative stimuli.

The differences between sentences/narratives versus single words/word pairs reported above remained when we controlled for the type of task (Figure S2B). In detail, we analysed a subset of the sentence/narrative and single‐word/word‐pair datasets, focusing only on studies where participants were required to make a semantic choice (as opposed to passive comprehension or answer generation). Even when the analyses were limited to choice tasks only, the results were consistent with those obtained when all tasks were included in the analysis—in detail, the right (and the left) ATL showed activation for sentences/narratives but not for single words/word pairs. This suggests that the type of task did not influence the differential activations observed between sentences/narratives versus single‐word/word‐pairs processing.

### 3.2 Semantic Control (Aim 2)

#### 3.2.1 Words Versus Sentences—Separate ALE Meta‐Analyses

**FIGURE 3:**
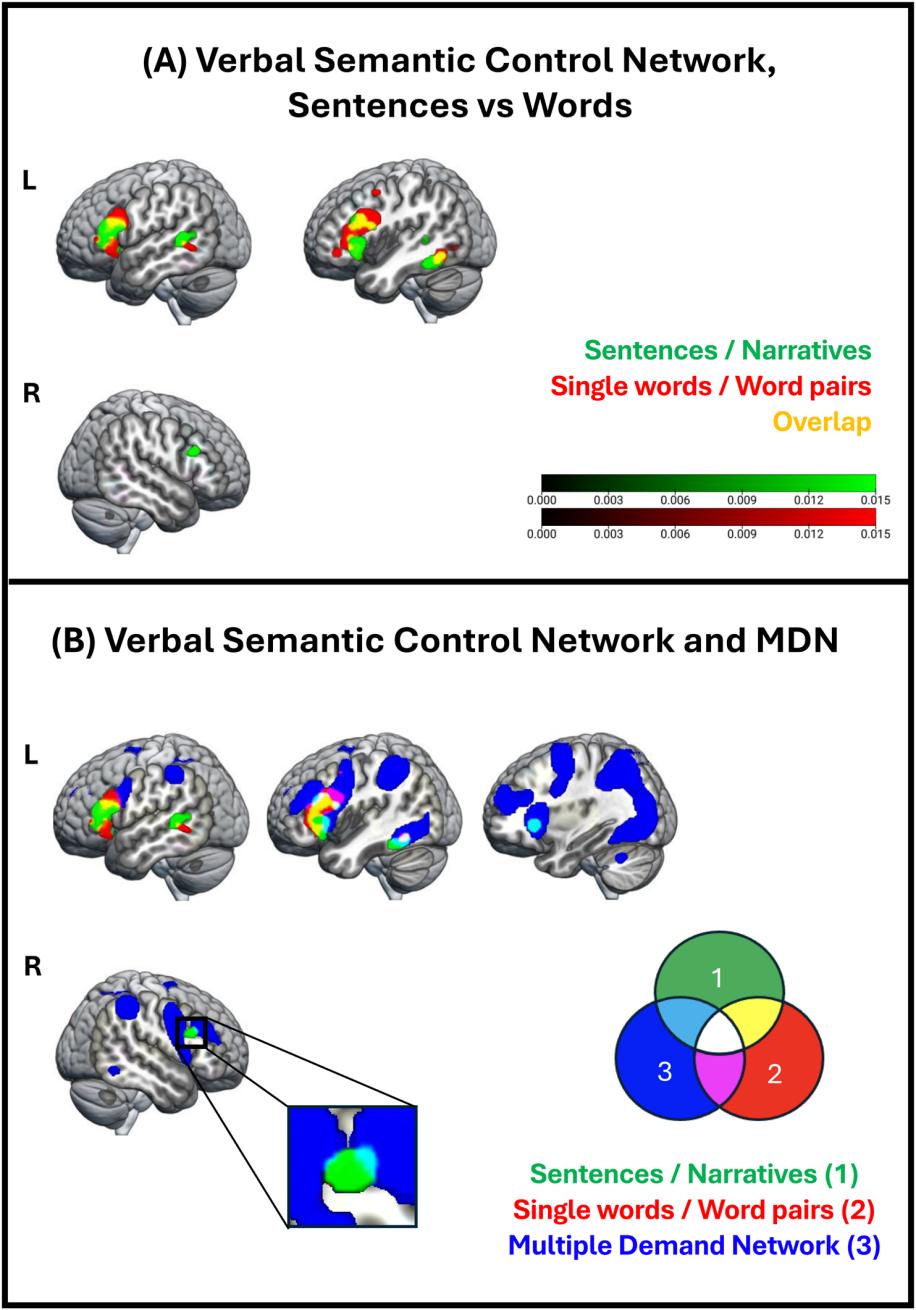
Verbal semantic control. (A) Brain regions reliably activated during hard > easy semantic tasks or conditions, split by stimulus type (red: single words/word pairs, green: sentences/narratives) overlaid. Colour bar: ALE‐values; cluster forming threshold: *p* < 0.001 (uncorrected); cluster extent correction: family‐wise error (FWE) *p* < 0.001. (B) The verbal semantic control network, split by stimulus type (red: single words/word pairs, green: sentences/narratives), and the multiple‐demand network (MDN) identified by Fedorenko et al. (2013) (blue), overlaid. Colour codes are explained in the Venn diagram.

To examine whether the right language network supports the same semantic functions as the left hemisphere but is recruited only when lexical demands increase (Aim 2), we conducted two ALE meta‐analyses, one including only semantic tasks with single‐word/word‐pair stimuli and another including only semantic tasks with sentence/narrative stimuli. The ALE meta‐analyses included activation coordinates derived from hard > easy semantic task or condition contrasts (see Figure 3A). For completeness, in Supporting Information we report the results of an ALE meta‐analysis including both data sets (see Figure S3). Activation coordinates for verbal semantic control are reported in Table S6.

In line with our hypotheses, tasks or conditions with higher semantic control, as compared to those with lower semantic control, engaged the right IFG Pars Triangularis and Opercularis. This effect was only observed during tasks requiring higher semantic combinatorial demands, that is, tasks using sentences/narratives as stimuli. Instead, increased semantic control demands in tasks using single words/word pairs did not activate the right hemisphere (Figure 3A). The right IFG is part of the right language network and was not observed in the general verbal semantic cognition results (see Figure 2A/Figure S2A).

For all types of stimuli, increased semantic control demands engaged left frontal regions, particularly the IFG Pars Triangularis and Opercularis. The posterior temporal lobe, including left pITG and pMTG, seemed more engaged during hard versus easy semantic processing, across both stimulus types. However, left posterior superior temporal gyrus (STG) was modulated by semantic control demands only during sentence/narrative processing.

#### 3.2.2 Words Versus Sentences—Subtraction Analysis Between ALE Meta‐Analyses

To ensure the stimulus specificity of the effect observed in the right IFG for semantic control in sentences/narratives as compared to single words/word pairs (Aim 2), we conducted a further direct subtraction analysis. In fact, GingerALE does not currently support a formal disjunction analysis (i.e., statistically identifying regions activated in one domain but not the other). Therefore, we used subtraction analysis to statistically compare activation likelihoods between stimuli conditions. This approach identifies regions that show significantly greater convergence in one stimulus domain and offers more robust inference than the outcome of separate ALE meta‐analyses. While this does not constitute a disjunction in the strictest sense, it provides a more rigorous test of domain specificity within the limits of current ALE methodology.

Interestingly, although separate ALE meta‐analyses revealed that the right IFG was significantly activated during semantic processing of sentences/narratives, but not during single words/word pairs, the subtraction analysis did not yield statistically significant differences in this region, even when examined with a more liberal threshold (see Table S6).

#### 3.2.3 Semantic Control—Overlap With MDN (Aim 4)

In line with our hypotheses, the brain regions modulated by semantic control in both the right and the left hemispheres showed a minimal overlap with the MDN. We found some overlap in the left insula, the left and right IFG Pars Triangularis and in the left IFG Pars Opercularis (Figure 3B).

### 3.3 Social Cognition and Its Neural Overlap With General Verbal Semantic Cognition (Aim 3)

#### 3.3.1 Nonverbal ToM and Social Cues Processing: Separate ALE Meta‐Analyses and Conjunction Analysis

**FIGURE 4:**
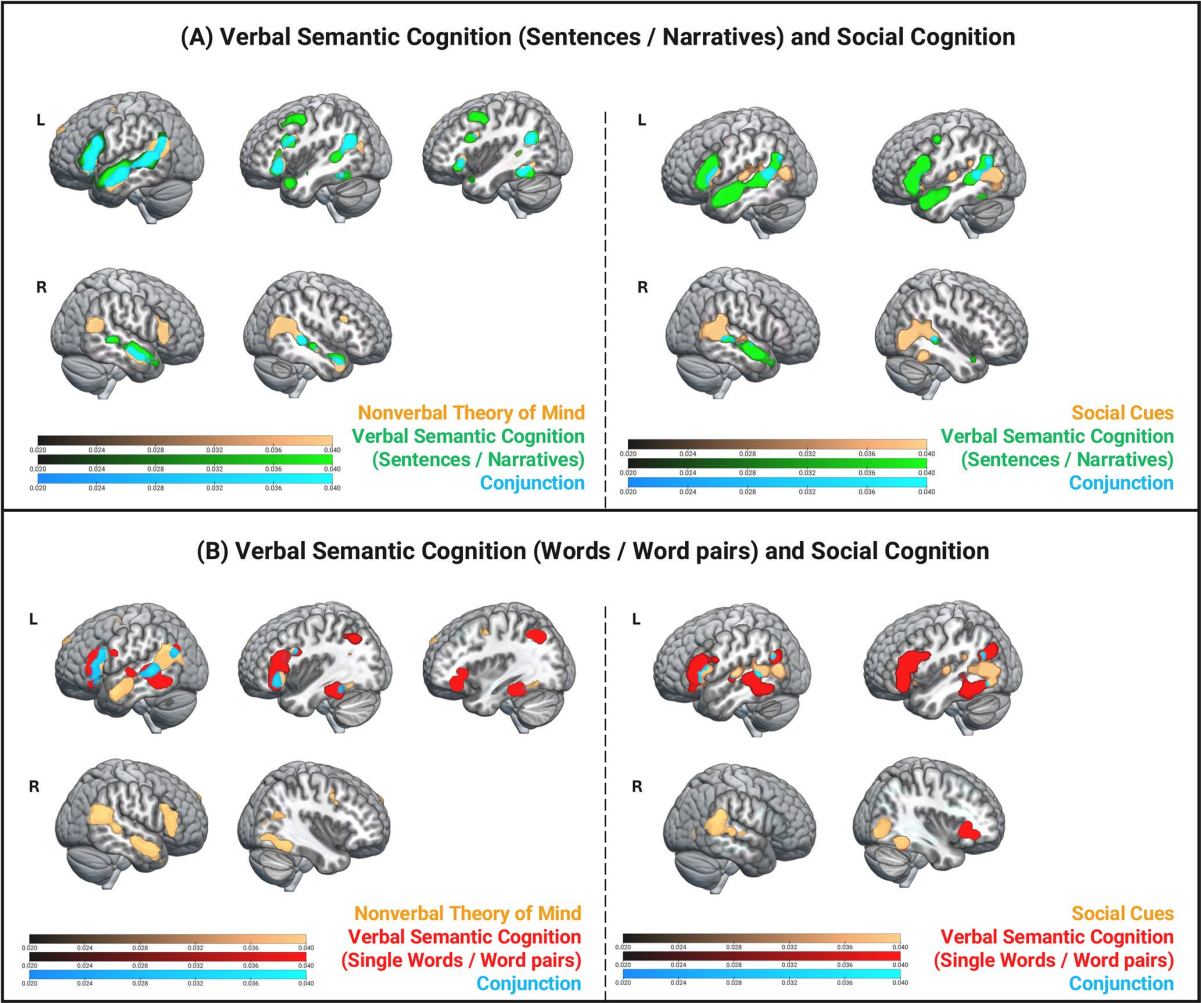
Neural overlap between social cognition and general verbal semantic cognition. (A) Brain regions activated during social cognition (orange; left panel—nonverbal Theory of Mind, right panel—Social Cues, against respective active baselines), general verbal semantic cognition (semantic versus non‐semantic or less semantic tasks) with sentences/narratives (green), and their neural overlap calculated via conjunction analysis (cyan), overlaid; Colour bar: ALE‐values; cluster forming threshold: *p* < 0.001 (uncorrected); cluster extent correction: family‐wise error (FWE) *p* < 0.001. Conjunction: ALE‐values, *p* < 0.001 (uncorrected), minimum cluster volume: 200 mm^3^. (B) Brain regions activated during social cognition (orange; left panel—nonverbal Theory of Mind, right panel—Social Cues, against respective active baselines), general verbal semantic cognition (semantic versus non‐semantic or less semantic tasks) with single words/word pairs (red), and their neural overlap calculated via conjunction analysis (cyan), overlaid; Colour bar: ALE‐values; cluster forming threshold: *p* < 0.001 (uncorrected); cluster extent correction: FWE *p* < 0.001. Conjunction: ALE‐values, *p* < 0.001 (uncorrected), minimum cluster volume: 200 mm^3^.

One ALE meta‐analysis revealed that nonverbal ToM processing engaged the ATL, the middle temporal gyrus (MTG), the pMTG and the ventral AG/TPJ in both hemispheres. Additionally, bilateral IFG, including Pars Triangularis and Orbitalis, as well as more posterior and dorsal portions of the frontal lobe, were also sensitive to nonverbal mentalisation (Figure 4, left side). The second ALE meta‐analysis revealed that processing Social Cues recruited a similar bilateral network as in nonverbal ToM, including pMTG and ventral AG/TPJ, except for the ATL.

Finally, for completeness, we performed a conjunction analysis between nonverbal ToM and Social Cues processing. The results are reported in Figure S4A. This conjunction analysis aimed to reveal the brain areas that are active during the perception of social content in the stimuli, independently from explicit task instructions to perform mentalisation on the stimuli. The right pMTG showed joint activation, alongside its left counterpart. Another brain region that was similarly engaged in both social cognition tasks was the left IFG Pars Opercularis. Activation coordinates for all ALE meta‐analyses involving social cognition are reported in Table S7.

#### 3.3.2 Social Cognition and Verbal Semantics With Social Content

The first conjunction analysis was conducted to examine whether the right language network engaged during meaning processing in sentence/narrative stimuli would be similarly recruited also during nonverbal ToM processing (Figure 4A, left). The results revealed several brain regions commonly recruited independent of the modality (verbal or nonverbal) and the type of tasks (semantic task with sentences/narratives and mentalisation task). In the right hemisphere, the ATL, MTG and pMTG were similarly engaged during the processing of meaning in sentences and narratives and nonverbal mentalisation. In the left hemisphere, a wider portion of the language network showed joint activation, including the IFG (Pars Orbitalis, Pars Triangularis and Pars Opercularis), the MTG, the ventral AG/TPJ and the pITG. The subtraction analysis revealed that the right ventral AG/TPJ and the right IFG were significantly more activated in nonverbal ToM processing compared to semantic processing of sentences/narratives (Figure S4B). On the other hand, semantic processing of sentences/narratives recruited the right anterior STG to a larger extent than nonverbal ToM processing.

The second conjunction analysis was conducted to examine whether the right language network during meaning processing in sentences/narrative stimuli would be similarly recruited also during the perception of Social Cues. That is, during the processing of social stimuli without explicit instructions to infer character intentions or engage in mentalisation (Figure 4A, right). Within the right language network, only the pMTG showed joint activation. Instead, in the left hemisphere, processing of both Social Cues and meaning in sentences/narratives activated the IFG (Pars Opercularis and Pars Triangularis) and the pMTG.

#### 3.3.3 Social Cognition and Verbal Semantics With Non‐Social Content

To ensure that the observed neural overlap between verbal semantic cognition and social cognition—particularly in the right ATL—was driven by similarities in social cognitive processes rather than semantic processing, we conducted a conjunction analysis between nonverbal ToM processing and verbal semantic cognition using only single‐word/word‐pair stimuli (Figure 4B, left). Because the latter should engage semantic processing but not social cognition, any neural overlap would likely suggest that activation of the right language network, and especially the right ATL, is not specific to social cognition. As expected, the results showed no joint neural activation in the right hemisphere, suggesting that the right ATL may have a key role in mentalisation.

A broader portion of the left hemisphere showed joint activation, including the IFG (Pars Opercularis and Pars Triangularis) and the pMTG—key brain regions of the semantic network (Lambon Ralph et al. 2017). Additionally, the left ventral AG/TPJ, which has been associated with both semantic processing (Binder et al. 2009) and mentalisation (Molenberghs et al. 2016; Schurz et al. 2014; Diveica et al. 2021), showed joint activation.

We then performed a conjunction analysis to reveal the neural overlap between the brain regions involved in the processing of Social Cues and those involved in semantic tasks with single words or word pairs (Figure 4B, right). The results revealed no neural overlap in the right hemisphere. In the left hemisphere, the neural overlap was observed in ventral AG/TPJ and in other brain regions of the semantic network (Lambon Ralph et al. 2017), including the IFG (Pars Triangularis and Pars Opercularis), the pMTG and the pITG.

### 3.4 General Verbal Semantic Cognition—High Versus Low Task Demands: Separate ALE Meta‐Analyses, and Subtraction Analyses (Aim 4)

**FIGURE 5:**
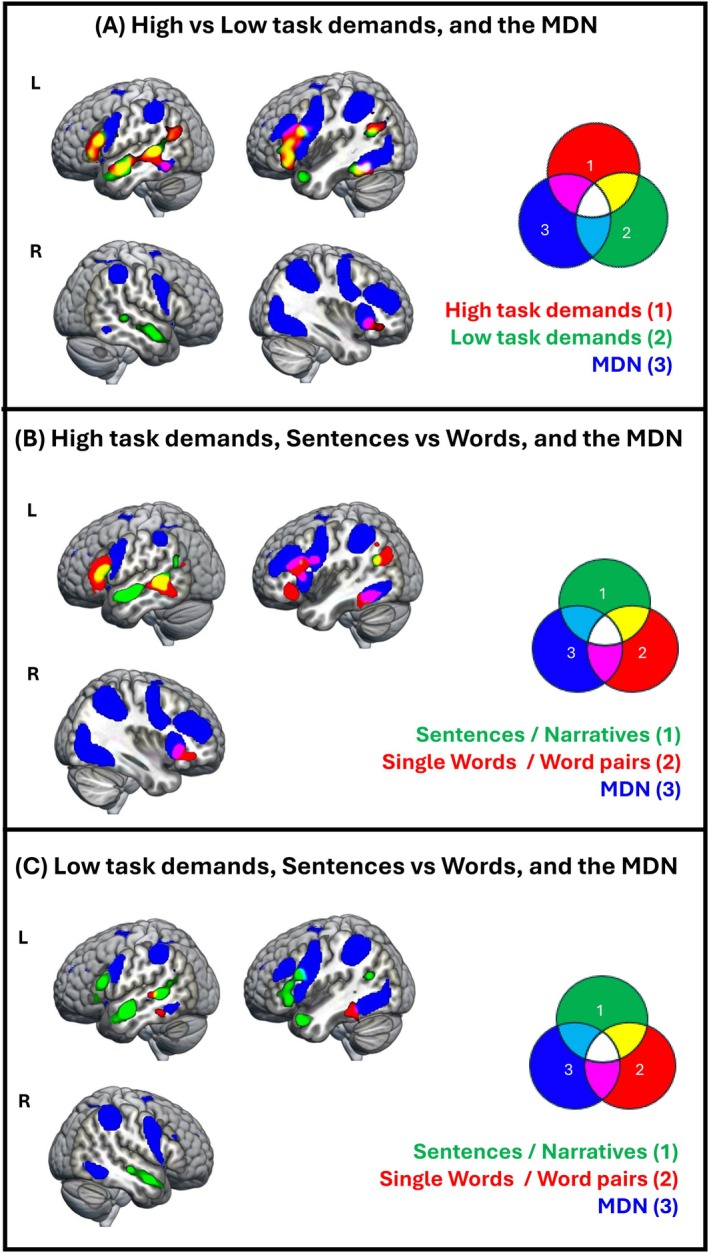
Brain areas activated during high versus low task demands in verbal semantic tasks, overlaid with the multiple‐demand network (MDN). (A) Brain regions active during semantic tasks with high task demands (red) and low task demands (green), (cluster forming threshold: *p* < 0.001 (uncorrected); cluster extent correction: family‐wise error (FWE) *p* < 0.001), and the MDN identified by Fedorenko et al. (2013), overlaid. (B) Brain regions active during semantic tasks with high task demands, split by stimuli type (sentences/narratives—green, single words/word pairs—red), (cluster forming threshold: *p* < 0.001 (uncorrected); cluster extent correction: FWE *p* < 0.001), and the MDN, overlaid. (C) Brain regions active during semantic tasks with low task demands, split by stimuli type (sentences/narratives—green, single words/word pairs—red), (cluster forming threshold: *p* < 0.001 (uncorrected); cluster extent correction: FWE *p* < 0.001), and the MDN, overlaid. Colour codes are explained in the Venn diagrams.

After examining the effect of increased semantic control, we also examined the effect of overall task difficulty (Stefaniak et al. 2021) on the neural network of general verbal semantic cognition. The results from the four ALE meta‐analyses revealed a minimal overlap between the language network and the MDN (Fedorenko et al. 2013) (Figure 5A). Activity in brain areas overlapping with the MDN was observed only when the semantic tasks loaded on external cognitive control demands (e.g., tasks requiring a semantic decision). However, this effect was not independent of the type of stimuli. High task demands modulated activity in brain areas overlapping with the MDN particularly when the semantic task involved single words/word pairs. This modulation was observed in the insula in the right hemisphere, and in the pITG and IFG (Pars Opercularis and Triangularis) in the left hemisphere. The peak coordinates for all ALE meta‐analyses on high and low task demands are reported in Table S8.

## 4 Discussion

In this ALE meta‐analysis, we investigated, for the first time, whether the right language network is differentially recruited by the semantic processing of single words/word pairs versus sentences/narratives (Aim 1). We then addressed whether this differential engagement reflects increased semantic control demands (Aim 2) and/or distinct processing demands associated with social cognition and ToM (Aim 3). In doing so, we also examined whether brain regions active during verbal semantic processing show overlap with the MDN or reflect domain‐specific responses to semantic and mentalising tasks. Finally, we also established whether the recruitment of MDN during language depends on external or semantic control demands (Aim 4). The key findings on the functional specialisation of the right language network and MDN for verbal semantic cognition are discussed below, along with additional results observed outside the territories of these networks.

### 4.1 Verbal Semantic Processing Engages the Right Language Network Differentially for Sentences/Narratives Versus Single Words/Word Pairs (Aim 1)

We demonstrated that the right language network is recruited differentially during semantic processing depending on whether the stimuli consist of single words/word pairs or sentences/narratives. In detail, the right ATL, right MTG and right pMTG were engaged during semantic processing of sentences/narratives. The same regions did not respond to single words/word pairs. The detailed interpretation of this result—namely, whether increased engagement of the right language network during sentence and narrative processing reflects heightened semantic control demands, involvement in social cognition, or both—will be discussed in depth in the following sections. However, it is worth noting that this finding aligns with previous studies showing that, as compared to the processing of single words and short phrases, narrative processing often produces a more bilateral pattern of activations within the language network (Branzi, Humphreys, et al. 2020; Branzi and Lambon Ralph 2023; Huth et al. 2016; Lerner et al. 2011; Xu et al. 2005).

The differential recruitment of the right temporal lobe between sentences/narratives versus single words or word pairs did not depend on differences in the type of baselines. In fact, since the rest baseline may reduce the likelihood of observing ATL semantic‐related activation, studies that employed rest as a baseline for semantic contrasts were excluded from both datasets.

Furthermore, we also demonstrated that the differential recruitment of the right temporal lobe between sentences/narratives versus single words/word pairs did not depend on differences in the type of tasks associated with the two types of stimuli. When we computed the same analysis but on a subset of data, focusing only on studies where participants were required to make a semantic choice, the results were consistent with those obtained when all tasks were included in the analyses.

Finally, the differential recruitment of the right temporal lobe, including the ATL, in sentences/narratives versus single words/word pairs, is unlikely to reflect differences in semantic control demands between the two types of stimuli. Our results show that semantic control demands do not modulate neural activity in the right ATL, regardless of stimuli type (sentences/narratives or single words/word pairs). While this result may seem to oppose previous findings revealing associations between right ATL activation and increased semantic control demands (Jung et al. 2021; Rice et al. 2018), it is worth noting that those studies have employed (1) stimuli such as abstract words with emotional, thought‐related, moral and social interaction valences (Jung et al. 2021); (2) tasks testing social semantic knowledge (e.g., matching job occupations to famous people) (Rice et al. 2018); and finally, (3) tasks that required processing information about human characters (Branzi, Humphreys, et al. 2020). Since all these tasks involve an element of social cognition, the right ATL activation may reflect participants' mentalising about characters in the narrative and/or adopting the perspective of famous individuals to match them with their occupations. If so, the right ATL might support semantic control demands, but only when the task or stimuli engage ToM and social cognition. Future studies will need to test this hypothesis.

To conclude, the differential recruitment of the right temporal lobe between sentences/narratives versus single words or word pairs, which is unlikely to be due to differences in baseline conditions, the types of tasks employed or semantic control demands, aligns with our hypothesis that sentences and narratives would lead to more extensive recruitment of the right language network.

It is important to note that right temporal lobe recruitment for meaning processing during sentences/narratives was particularly evident with auditory stimuli. Instead, sentence/narrative stimuli presented in visual (written) modality activated mainly the left ATL. The left bias for written stimuli found in the present study replicates previous results of auditory (bilateral) versus written (left) word processing (Marinkovic et al. 2003; Rice et al. 2015; Visser and Lambon Ralph 2011). Accordingly, a recent ALE meta‐analysis revealed that auditory stimuli during semantic processing engaged the ATL bilaterally, while visual stimuli engaged primarily the left ATL (Balgova et al. 2024).

Balgova et al. (2024) proposed that the recruitment of bilateral ATL during auditory stimuli could be understood in terms of general processing effort. This is because auditory stimuli reflect mainly sentences that require both rapid processing of individual words and combinatorial meaning, which could work the semantic system more vigorously than other types of stimuli. Be that as it may, here we show a substantial difference in the recruitment of the right temporal lobe between written and auditory sentences which does not fully support this hypothesis.

One possibility is that the right ATL is more strongly engaged during auditory stimuli because spoken verbal stimuli involve human voice recognition, allowing listeners to automatically infer details and activate social knowledge about a speaker's identity, such as their sex and age. In other words, as compared to written stimuli, auditory stimuli may represent a more inherently social format, which may lead to the recruitment of the right ATL. That said, it is important to note that differences between visual and auditory perceptual modalities are unlikely to be the primary factor driving the differential activation of the right ATL observed in sentence or narrative tasks compared with single‐word or word‐pair tasks. Indeed, when considering auditory stimuli alone, activation in the most anterior portions of the right ATL was observed particularly during sentence or narrative tasks. These findings are consistent with previous evidence indicating that the most anterior portion of the right ATL plays a key role in processing semantic information with social content (Olson et al. 2013).

Finally, we found that the right insula responded to semantic processing during single words/word pairs but not to sentence/narrative stimuli. The right insula is part of the MDN (Duncan 2010; Fedorenko et al. 2013). In language tasks, this brain region has been associated with executive control processes such as inhibition of the non‐target meaning of ambiguous words (Mason and Just 2007) and updating of semantic representation following semantic violations (Branzi, Humphreys, et al. 2020; Branzi and Lambon Ralph 2023). Accordingly, in the present study we found that the right insula responded to both semantic and non‐semantic (external/task) demands, consistent with the proposal that it may reflect domain‐general control processes (Duncan 2010; Fedorenko et al. 2013).

The observation that the right insula is active only when semantic tasks require response suppression and conflict resolution (i.e., tasks with high external task demands) indicates that this brain region is unlikely to play a central role in core language functions such as lexical/semantic retrieval and combinatorial semantics. Instead, it may be engaged to reduce interference during response selection, particularly when competition between target and non‐target semantic representations is artificially heightened by task demands.

### 4.2 Semantic Control Modulates Activity in the Right IFG (Aim 2)

Our first hypothesis was that the right language network would support the same functions as their homologue regions in the left hemisphere, and that areas within the right language network, particularly the right pMTG, the right IFG and the right dorsal AG/IPS, would be recruited when semantic control demands increase (Lambon Ralph et al. 2017). Accordingly, and in line with our hypothesis that due to greater semantic control demands tasks with sentences and narratives would recruit the right language network more extensively than single‐word or word‐pair tasks, we observed preferential recruitment of the right IFG (Pars Opercularis and Pars Triangularis) during sentence/narrative tasks for hard versus easy semantic conditions. While our results do not support a statistically robust stimulus‐specific effect under conservative contrast testing, the preferential engagement of the right IFG in sentence/narrative tasks is consistent with recent evidence that right IFG activity increases with greater semantic control demands during sentence or narrative processing (Branzi, Humphreys, et al. 2020; Silbert et al. 2014). The neural activity measured in the right IFG (Pars Opercularis and Pars Triangularis) was accompanied by a similar but more pronounced difficulty‐related effect in the left IFG, in line with previous reports (Jung et al. 2021; Krieger‐Redwood et al. 2015; Lambon Ralph et al. 2017; Quillen et al. 2021; Wu and Hoffman 2024).

Interestingly, increased activity in the right IFG was not observed for semantic control (hard versus easy semantic conditions) during single‐word/word‐pair tasks. This discrepancy may arise because, compared to single‐word or word‐pair tasks, combining meanings across multiple words and sentences likely imposes greater semantic control demands, for which left IFG engagement alone may not be sufficient to optimise performance. In line with the hypothesis that right IFG may be recruited only when semantic control demands are especially high, the right IFG was not activated in the general verbal semantic cognition ALE meta‐analysis.

The additional involvement of right IFG, relative to the left IFG, during semantic control is in accord with the ‘variable neuro‐displacement’ hypothesis (Jung et al. 2021; Stefaniak et al. 2020). This proposes that cognitive systems are formed with dynamic, spare processing capacity, which balances energy consumption against performance requirements and can be resilient to changes in performance demands. In the context of the present data, healthy language processing would be supported by both left and right language networks, with a bias towards the left system (see results from the general verbal semantic cognition ALE meta‐analysis). The right language areas would be downregulated unless the system is under high pressure (increase of semantic control demands during sentences/narratives) to optimise performance. Accordingly, the right IFG has been shown to be engaged during semantic tasks in post‐stroke aphasic patients with left hemispheric damage to a larger extent as compared to healthy participants (for a review, see Turkeltaub et al. 2011).

These results highlight the importance of the right IFG for language processing in healthy individuals and align with patient studies demonstrating its key role in language recovery following left‐hemisphere damage. Numerous neuroimaging studies have identified right hemisphere activation in individuals with post‐stroke aphasia, with this activity linked to improvements in speech production (Griffis et al. 2017; Qiu et al. 2017). For instance, increased activity in the right inferior prefrontal cortex has been observed during partial recovery of propositional speech following post‐stroke damage to Broca's area (specifically the Pars Opercularis) (Blank et al. 2003), as well as in patients with low‐grade glioma (Thiel et al. 2001).

Contrary to our hypotheses, we found no evidence of the involvement of the right pMTG and the right dorsal AG/IPS in response to increased semantic control demands. Although few studies have found right dorsal AG/IPS activity to be modulated by semantic control demands (Branzi, Humphreys, et al. 2020; Branzi, Martin, et al. 2020; Jung et al. 2021), many others have failed to show consistent involvement of this region in any aspect of semantic cognition (Jackson 2021). This observation does not uniquely apply to the right dorsal AG/IPS, but also to the left dorsal AG/IPS (Jackson 2021).

Since dorsal AG/IPS is part of the MDN (Duncan 2010; Fedorenko et al. 2013), the occasional identification of this brain region during semantic tasks may reflect domain‐general control requirements imposed by the task at hand, rather than semantic control. Accordingly, in our study we show that increased external task demands, rather than semantic control demands, modulate neural activity in the left dorsal AG/IPS.

In the present study, we found that the social content of the stimuli rather than semantic control demands upregulated neural activity in the right pMTG. This result will be discussed in greater detail in the next section. However, it is worth noting that, while previous studies have shown that the right pMTG responds to increased semantic control demands (Branzi, Humphreys, et al. 2020; Branzi, Martin, et al. 2020; Jung et al. 2021), this study and earlier reports suggest that such evidence is inconsistent (Jackson 2021). The right pMTG activity, modulated by semantic control, may reflect the use of stimuli with social content (Branzi, Humphreys, et al. 2020; Branzi, Martin, et al. 2020; Jung et al. 2021), which might have engaged social cognition processes.

Importantly and in line with our hypothesis, only a minimal overlap between the MDN and the semantic control network was observed. This overlap concerned the left dorsal IFG. These results align with previous data showing some involvement of the MDN during semantic tasks (Branzi, Humphreys, et al. 2020; Branzi and Lambon Ralph 2023; Hodgson et al. 2021). Here, we demonstrate that this brain region is engaged in semantic tasks only when lexical semantic competition between representations is artificially heightened due to task design choices, rather than reflecting fundamental verbal semantic processes.

### 4.3 Semantic Control Beyond the Right Hemisphere: Stimulus Type Modulates the Recruitment of the Left Posterior Temporal Lobe

We observed that the semantic control network fractionates depending on the type of stimuli, not only along the left‐right dimension but also along the dorsal‐ventral dimension. In the left posterior middle temporal lobe, the pMTG and the pITG exhibited a graded shift in their preference for controlling meaningful stimuli, with the pMTG being more involved in sentence and narrative processing, whereas the pITG was more involved in tasks with single words and/or word pairs.

Previous studies have demonstrated that pITG responds particularly to hard phonological and semantic tasks (Hodgson et al. 2021) and is implicated in the extended MDN (Assem et al. 2020). It is unclear if this region reflects shared control processes for language subdomains or rather across a broad set of linguistic and non‐linguistic domains. Our results suggest that this brain region may reflect some sort of language‐specific control. In fact, in our study the left pITG was (1) recruited during semantic processing of word stimuli irrespective of the external task demands and (2) found sensitive to semantic control demands. Accordingly, Hodgson et al. (2021) found left pITG implicated in both phonological and semantic control, but not in nonverbal working memory. This, along with the fact that the left pITG has been found in MDN assessments which did not specifically exclude verbal stimuli (Assem et al. 2020; Fedorenko et al. 2013), leaves open the possibility that left pITG supports language‐specific control processes.

However, left pITG has also been implicated in nonverbal executive function tasks, such as task‐switching (Lemire‐Rodger et al. 2019; Schumacher et al. 2019). Therefore, its involvement in semantic tasks may reflect a domain‐general function shared with task‐switching paradigms, such as for instance inhibition of the competing (semantic or non‐semantic) representations. This function may be particularly required in tasks with single words and/or word pairs, which often include target words presented concurrently with competing semantic distractors that need to be inhibited to perform the semantic task.

### 4.4 The Right Temporal Lobe Supports Verbal Semantics, but Only for Stimuli With Social Content (Aim 3)

Sentences and narratives typically provide an acting/feeling character(s) and ‘schema or situation models’, that is, mental representations that summarise the interaction among entities and the environment in a scene or event (Ranganath and Ritchey 2012). In this context, it is likely that, besides semantic processing, language comprehension engages social cognition to support mentalisation and the processing of Social Cues.

Accordingly, we hypothesised that some parts of the right language network previously associated with social cognition and ToM, that is, the right ventral AG/TPJ, the right ATL (Olson et al. 2013; Saxe et al. 2004; Saxe and Wexler 2005; Turker et al. 2023) and the right pMTG (e.g., Hodgson et al. 2023), would be engaged during semantic processing, but only in tasks with stimuli containing social elements. We tested this hypothesis by examining the neural overlap between verbal semantic cognition − measured in social (sentences and/or narratives) and non‐social (single words/word pairs) stimuli, as separately − and social cognition − (measured in nonverbal ToM and in Social Cue processing tasks, as separately).

Contrary to our hypotheses, we did not find any overlap in the right ventral AG/TPJ between the social cognition networks (nonverbal ToM and Social Cue processing) and the verbal semantic network. In line with previous results (Balgova et al. 2024, 2022; Hodgson et al. 2023), the right ventral AG/TPJ seems to respond specifically to social cognition. However, the overlap between the nonverbal ToM network and the verbal semantic network was observed in the left ventral AG/TPJ, but only for sentences/narratives.

The laterality of ventral AG/TPJ involvement in social cognition is unclear. Neuroimaging evidence has shown that ToM engages this area bilaterally (Diveica et al. 2021). Yet, some have argued that the selectivity of this region for ToM is in the right hemisphere (Saxe and Wexler 2005). Others, instead, have argued that selectivity is in the left hemisphere (Aichhorn et al. 2006, 2009). In semantic cognition, activation of this region is generally left lateralised for verbal stimuli (Branzi, Humphreys, et al. 2020; Branzi et al. 2021; Branzi and Lambon Ralph 2023; Humphreys et al. 2024; Humphreys and Lambon Ralph 2015). Our results further demonstrate that this is the case, but only when semantic cognition involves sentences/narratives with social content. This suggests that left ventral AG/TPJ may play a role in social and mental inference‐making processes during semantic tasks.

Additionally, and in accord with our hypotheses, we found that the right ATL (including the temporal pole) was similarly engaged in nonverbal ToM and verbal semantics, but only when semantic processing involved stimuli with social content (sentences/narratives). Instead, while the semantic processing of single words/word pairs engaged the left ATL (anterior STG), no activity was observed in the right ATL during semantic processing of single words/word pairs. Finally, we observed activity in the right pMTG in response to (1) nonverbal tasks with explicit instructions to perform ToM; (2) verbal semantic tasks including sentences/narratives possibly resulting in participants performing mentalisation about the character(s); and finally, (3) tasks requiring the processing of Social Cues, but with no explicit instructions to perform mentalisation. All in all, these results indicate that the right but not the left ATL and pMTG are specifically recruited when social cognition is likely involved in the task.

Note that the similar recruitment of the right temporal lobe during both semantic and social cognition cannot be explained by the similarity in the type of stimuli employed. In fact, semantic cognition tasks and social cognition tasks included substantially different stimuli and, with the exception of prosody processing, presented in different formats (verbal versus nonverbal).

The similar engagement of right temporal lobe regions in verbal semantic tasks and nonverbal ToM tasks might be because both engage semantic processing (see Balgova et al. 2024; Binney and Ramsey 2020). According to this hypothesis, a recent ALE meta‐analysis study of functional neuroimaging studies revealed a great overlap between the ALE maps for ToM and semantic cognition, after controlling for critical aspects such as baseline type, stimulus format and type of perceptual modality (Balgova et al. 2024). Interestingly, however, the overlap was mostly observed in brain areas that in our study were uniquely involved in sentences and/or narratives, for example, the bilateral ATL, including the temporal pole and anterior superior temporal sulcus, as well as the right MTG and the left ventral AG/TPJ, all key regions for semantic processing (Binder et al. 2009).

Thus, our results indicate that at least another explanation for the overlap between ToM and semantic cognition may be viable. That is, the processing of stimuli such as sentences and narratives may engage social cognition besides semantic processing. In fact, we found that only sentence and narrative tasks—likely involving a social cognition component—showed neural overlap (conjunction) with ToM and Social Cue tasks in brain regions typically associated with social cognition. In contrast, single‐word and word‐pair tasks, that is, stimuli without social content, did not exhibit this overlap. Therefore, although semantic cognition is integral to social cognition (Binney and Ramsey 2020) and some neural overlap between these two domains can be explained as such, the neural overlap observed in the right ATL (anterior MTG) and the right pMTG may in part reflect similar engagement of social cognition, rather than semantic processes.

This aligns with studies on both healthy participants and patients, showing that the right ATL may be particularly tuned to social cognition (Gainotti 2015; Pobric et al. 2016; Rankin et al. 2006; Rice et al. 2015). For instance, the right versus the left ATL presents stronger connectivity with the orbital frontal cortex via the uncinate fasciculus (Papinutto et al. 2016), that is, brain structures known to be important for social cognition (Oishi et al. 2015; Rolls and Grabenhorst 2008; Von Der Heide et al. 2013). Furthermore, patient studies reveal that performance in empathy and emotion processing tasks is negatively correlated to the degree of right ATL atrophy (Leigh et al. 2013; Papinutto et al. 2016; Rankin et al. 2006; Seeley et al. 2005). Along the same lines, right ATL atrophy also negatively correlates with performance in ToM tasks, as shown in patients with frontotemporal dementia and ischaemic stroke (Irish et al. 2014; Younes et al. 2022). Finally, fMRI studies involving aphasic patients have demonstrated the importance of right ATL for language tasks with social elements. For instance, in individuals with a history of aphasia, activation of the right superior ATL during narrative listening correlates with sentence comprehension (Crinion and Price 2005). Furthermore, in chronic aphasia, the strength of interhemispheric connectivity between the superior ATLs correlates with both single‐word and sentence comprehension (Warren et al. 2009). This evidence suggests a link between the involvement of the right ATL and the degree of recovery from comprehension impairments (Crinion and Price 2005; Warren et al. 2009). The right ATL, therefore, is not only implicated during healthy language processing but also supports language recovery after left‐hemispheric damage, especially with respect to social‐semantic deficits, highlighting its potential in post‐stroke therapy.

The predominant involvement of right temporal lobe regions, and especially the right ATL, during verbal semantic tasks with social content further suggests that this brain region is particularly specialised for social semantic processing. This observation aligns with the ‘fully specialised’ account of ATL function, which posits a division of labour between the hemispheres: the left ATL would be primarily involved in non‐social semantic knowledge, whereas the right ATL would be preferentially engaged in social semantic processing (Gainotti 2007, 2014; Olson et al. 2007; Snowden et al. 2004, 2012; Tranel et al. 1997).

Our findings, however, are also consistent with a ‘graded functional specialisation’ account of ATL function, emerging as a result of asymmetric connectivity between ATL subregions and primary input/output areas, a proposal that to date, has received the strongest empirical support within the literature (Binney et al. 2012; Pascual et al. 2015; Plaut 2002; Lambon Ralph et al. 2001; Rice et al. 2015; Schapiro et al. 2013; Visser et al. 2012; Visser and Lambon Ralph 2011). Similar to the fully specialised view, this account acknowledges functional differences between the left and right ATLs. However, it posits that these differences are relative rather than absolute since semantic representations are distributed (Schapiro et al. 2013). One influential model supporting this account is the ‘hub‐and‐spoke’ model (Lambon Ralph et al. 2017), which proposes that the core computational role of the ATLs is to integrate modality‐specific inputs to construct coherent semantic representations. These representations emerge over time from the extraction of statistical regularities across input modalities. Because the connectivity profiles differ across ATL subregions, functional variation arises both within and between hemispheres. For example, the anterior STG and MTG exhibit stronger structural connectivity with auditory processing pathways (Binney et al. 2012), which likely contributes to their preferential engagement during semantic tasks with spoken words, in contrast to tasks using pictorial stimuli (Visser and Lambon Ralph 2011; Visser et al. 2012) or written word stimuli (Marinkovic et al. 2003; Rice et al. 2015). Crucially, hemispheric differences in connectivity also contribute to lateralised functional specialisation. Intra‐hemispheric connections are substantially more prevalent than inter‐hemispheric ones, resulting in stronger links between the left ATL and left‐lateralised regions associated with orthographic processing and language production (Binney et al. 2012). Conversely, semantic stimuli and representations involving people may rely more heavily on right‐lateralised pathways, in part due to the right hemisphere's dominance in face processing within the ventral occipitotemporal cortex (Behrmann and Plaut 2020; Plaut and Behrmann 2011). As a result of this graded specialisation, the engagement of the right ATL and the right pMTG may be particularly pronounced when semantic tasks concern people and likely involve processes such as mentalising.

To conclude, we propose that the ATL—including both STG and MTG and particularly the right ATL—contributes to both nonverbal ToM tasks and semantic tasks with sentences by providing conceptual information to constrain inferences about the intentions and actions of others (Binney and Ramsey 2020). This includes, for example, information about an actor's state of mind, independently from whether an explicit descriptor is present. Instead, the right pMTG may reflect control processes to regulate and select conceptual information relevant for making social inferences.

### 4.5 Increased External Task Demands During Verbal Semantic Processing Recruit the MDN (Aim 4)

Language requires applying cognitive operations (e.g., retrieval, prediction, and combinatorial processes) similar to those used in other cognitive domains. This has led to the hypothesis that the brain contains domain‐general circuits responsible for carrying out these operations, with language relying on these circuits (Abutalebi and Green 2007; Fitch and Martins 2014; Green and Abutalebi 2013; Hasson et al. 2018; Koechlin and Jubault 2006). Whilst there is evidence for domain‐general executive processes and MDN engagement in diverse linguistic phenomena, including ambiguity processing (Mcmillan et al. 2013; Novais‐Santos et al. 2007; Rodd et al. 2005), high surprisal (Shain et al. 2020) and grammatical violations (Kuperberg et al. 2003; Mollica et al. 2020; Nieuwland 2012), some researchers have questioned the importance of domain‐general executive processes and the MDN to support core language operations (for review, see Fedorenko and Shain 2021).

First, our results are in line with previous reports by showing that some MDN brain regions are active during semantic cognition (Hodgson et al. 2021; Branzi and Lambon Ralph 2023; Branzi, Humphreys, et al. 2020). These brain areas include the dorsomedial prefrontal cortex (including supplementary motor area and pre‐supplementary motor area) (Branzi et al. 2022; Branzi, Martin, et al. 2020; Geranmayeh et al. 2014), the left pITG (Assem et al. 2020; Duncan 2010), the right insula, the left IFG Pars Opercularis and Triangularis bordering with the premotor cortex.

Second, and in accordance with evidence that the MDN does not support core language computations (Blank and Fedorenko 2017; Branzi and Lambon Ralph 2023; Diachek et al. 2020), our results indicate that rather than being modulated by semantic control demands specifically, MDN regions are modulated by external task demands (e.g., inhibiting concurrent distractors and making choices between multiple conflictual options), which often accompany core language demands (e.g., lexical retrieval, combinatorial semantics, etc.).

In detail, we found that high task demands modulated activity in the right insula, the left pITG and the left IFG Pars Opercularis and Triangularis. This result is in accord with previous studies that have shown that, unless language processing is associated with a secondary task, the MDN is not recruited during verbal semantic processing. For instance, Wright et al. (2011) showed that some frontal MDN areas are engaged during a lexical decision task, but not in passive listening to the same materials. In a similar vein, Diachek et al. (2020) compared MDN engagement in language experiments requiring passive language comprehension (visual or auditory) with those involving an additional task, such as answering comprehension questions or determining semantic associations. The language network responded equally strongly during tasks with and without an additional task. Instead, the MDN was recruited only in the presence of an additional task.

Interestingly, the effect of external task demands on the recruitment of the MDN was not fully independent of the type of stimuli. In fact, except for the left pITG, the overlap between verbal semantic processing and the MDN was only observed in high demand tasks with single words/word pairs. This may reflect overall differences in task type between single word/word pair and sentence/narrative tasks. Even if we carefully split the data into high versus low task demands, following the same criteria for both types of stimuli (narratives/sentences and single words/word pairs), there may still be some differences in the external demands imposed by tasks typically associated with these stimuli. For example, sentences/narratives associated with a secondary choice task often include a yes/no question about the sentence or narrative. Instead, single‐word/word‐pair tasks requiring a choice often include a probe word, a target word, and one to three distractors. The presence of multiple‐choice options likely places more demands on domain‐general control processes and the MDN (Assem et al. 2020; Duncan 2010) to perform target‐nontarget discrimination, shifts in attention and inhibition of nontarget responses.

Overall, our results indicate that the verbal semantic network may include regions specifically involved in semantic representation (e.g., the ATL) and control (such as the ventral IFG and pMTG) of meaningful representations, as well as regions associated with domain‐general control processes, including the left dorsal IFG, left pITG and right insula, which may only be recruited when external task demands are heightened.

## 5 Limitations

The use of a meta‐analysis approach allowed us to shed light on the broader organisation of the language network, and especially the role of the right language network for semantic processing, while also uncovering previously hidden relationships between semantic processing, social cognition and domain‐general executive control. Meta‐analyses have the advantage of synthesising information from a wide range of studies and cognitive domains. However, due to the nature of the methodological approach, they do not allow full control over stimulus or variable manipulation. In our study, for example, it was not possible to separate the effects of ‘stimulus type’ and ‘socialness’ on the recruitment of the right language network. Future research will need to address this issue using controlled experimental designs in which these variables are orthogonally manipulated. Finally, ALE meta‐analyses lack precision in spatial resolution. This may represent a limitation, especially when examining adjacent regions, such as the IFG and insula. Therefore, it will be important to replicate the results of the present study using within‐subject designs as well as subject‐specific localiser approaches, which provide greater sensitivity and functional resolution.

## 6 Conclusions and Future Directions

To conclude, we aimed to determine the role of the right language network: is it an additional resource along the left language network, that is engaged when semantic task demands increase or is it a specialised network responsible for social cognition? Our results show that within the right language network some brain regions support similar semantic functions as in the left hemisphere, whilst instead some other brain regions seem to be more tuned to social cognition functions. In detail, our findings reveal that the recruitment of the right temporal lobe during semantic processing is influenced by the type of stimuli and likely their socialness, rather than increased semantic demands. One possible explanation for these observations is that verbal semantic tasks involving social stimuli may engage ToM processing and/or other aspects of social cognition. In turn, this would activate the semantic network along with additional regions in the right hemisphere, which are more tuned for processing stimuli with social content. Alternatively, these regions may belong to a single, widely distributed yet functionally integrated semantic network, with systematic variation in the engagement of specific nodes across hemispheres driven by task‐related or stimulus‐related factors, such as the input modality and the social nature of the semantic stimuli. Our data are compatible with both explanations. Future studies will have to employ within‐subject designs and assess the factors that drive the recruitment of the right hemisphere within and across social and semantic cognition domains.

Furthermore, our findings revealed that the right IFG supports semantic control in sentence and narrative processing. Interestingly, nonverbal ToM processing also engaged the right IFG. One possible explanation for this result, in line with the semantic control results, is that ToM tasks, overall, may place greater semantic control demands compared to typical semantic tasks. In other words, ToM tasks could be classified as ‘hard semantic conditions’. Nonverbal ToM experiments often include tasks in which participants are asked to switch between their own and a different character's visual, cognitive, and emotional perspective. Social stimuli that describe a character's mental states or intentions (Pexman et al. 2023), regardless of modality, will likely activate a wide range of semantic associations coupled with different perspectives, possibly creating incongruency with the observer's own perspective. This might increase the overall demand for semantic control. That said, it remains unclear whether the right IFG activity observed during ToM processing reflects the same control processes as those involved in the semantic domain (Binney and Ramsey 2020). Future research will need to address this question using within‐subject designs to determine whether the distinction between knowledge representation and cognitive control observed in the semantic domain also applies to the social domain.

## Funding

The authors have nothing to report.

## Conflicts of Interest

The authors declare no conflicts of interest.

## Supporting information


**Figure S1:** The flowchart summarises the procedures for studies identification, coordinates extraction and key statistical analyses relative to the results reported in the Supporting Information; the analysis steps are mapped to corresponding Supplementary Figures via different colour codes.

**Figure S2:** General verbal semantic cognition. (A) Brain regions activated during all verbal semantic > non‐semantic (or less semantic) tasks. Colour bar: ALE‐values; cluster forming threshold: *p* < 0.001; cluster extent correction: family‐wise error (FWE) *p* < 0.001. (B) Brain regions activated during verbal semantic processing, only including tasks in which participants had to perform a choice, split by stimuli type (green: sentences/narratives, red: single words/word pairs, yellow: overlapping brain regions). Colour bar: ALE‐values, *p* < 0.001, cluster extent correction: FWE *p* < 0.001.

**Figure S3:** Brain regions reliably activated during all hard versus easy semantic tasks or conditions. Colour bar: ALE‐values; cluster forming threshold: *p* < 0.001 (uncorrected); cluster extent correction: family‐wise error (FWE) *p* < 0.001.

**Figure S4:** Social processing. (A) Individual ALE meta‐analyses and conjunction analyses of nonverbal Theory of Mind processing (green), Social Cues processing (red) and their conjunction (blue). Colour bar: ALE‐values; cluster forming threshold: *p* < 0.001 (uncorrected); cluster extent correction: family‐wise error (FWE) *p* < 0.001. Conjunction: ALE‐values, *p* < 0.001, minimum cluster volume: 200 mm^3^. (B) Brain regions differentially engaged depending on task and stimulus type (orange: nonverbal ToM tasks versus sentences/narratives; green: sentences/narratives versus nonverbal ToM tasks), calculated via t‐test/subtraction analysis. Colour bar: *Z*‐scores, cluster forming threshold: *p* < 0.001 (uncorrected), minimum cluster volume: 200 mm3.

**Table S1:** List of studies included in the *Semantic Cognition* meta‐analysis. Studies have been categorised based on *type of* *stimuli* (single words/word pairs or sentences/narratives); *modality* (visual or auditory or both), *type of task* (choice or comprehension or production/internal generation (thinking about the right answer)); *task demands* (high task demands or low task demands), and whether the study was included in the *subset* analysed in the choice tasks contrasts. Number of participants is provided (*N*). The coordinates *(* *X*, *Y* *and* *Z* *)* are provided in MNI space; coordinates from papers using Talairach coordinates have been converted to MNI using the Lancaster transform (‘icbm2tal’) via GingerALE (Laird et al. 2010; Lancaster et al. 2007). Additionally, tasks used in experiments, as well as the contrasts used for the analysis are described in the column *Task summary & Contrast*.
**Table S2:** List of studies included in the *Semantic Control* ALE meta‐analysis. Studies have been categorised based on *type of* *stimuli* (single words/word pairs or sentences/narratives); *modality* (visual or auditory or both), *type of task* (choice or comprehension or production/internal generation (thinking about the right answer)); and *task demands* (high task demands or low task demands). Number of participants is provided (*N*). The coordinates *(* *X*, *Y* *and* *Z* *)* are provided in MNI space; coordinates from papers using Talairach coordinates have been converted to MNI using the Lancaster transform (‘icbm2tal’) via GingerALE (Laird et al. 2010; Lancaster et al. 2007). Additionally, tasks used in experiments, as well as the contrasts used for the analysis are described in the column *Task summary & Contrast*.
**Table S3:** List of studies included in the *Nonverbal Theory of Mind* ALE meta‐analysis. Studies have been categorised based on *type of task* (choice or comprehension); and the type of Theory of Mind required to perform (*contrast*—inferring intention or mental state or predicting behaviour or false belief reasoning). Number of participants is provided (N). The coordinates *(* *X*, *Y* *and* *Z* *)* are provided in MNI space; coordinates from papers using Talairach coordinates have been converted to MNI using the Lancaster transform (‘icbm2tal’) via GingerALE (Laird et al. 2010; Lancaster et al. 2007).
**Table S4:** List of studies included in the *Social Cues* ALE meta‐analysis. Studies have been categorised based on *type of social cue* (biological motion or face perception or prosody). The *contrast* taken from each study is specified. Number of participants is provided (*N*). The coordinates *(* *X*, *Y* *and Z* *)* are provided in MNI space; coordinates from papers using Talairach coordinates have been converted to MNI using the Lancaster transform (‘icbm2tal’) via GingerALE (Laird et al. 2010; Lancaster et al. 2007).

**Table S5:** All activation clusters and local maxima for *Verbal Semantic Cognition*. Coordinates *(X, Y* *and* *Z* *)* are reported in the MNI coordinate system; Clust no: cluster number in the individual contrast; ALE: activation likelihood estimate values output from GingerALE, along with *p* and *Z* values; Cytoarchitecture: cytoarchitectonic information for foci assigned by the JuBrain Anatomy Toolbox (SPM), based on the Maximum Probability Map; % cyto: probability of the coordinate falling into the specified Cytoarchitecture, as an output of the Anatomy Toolbox; Assignment: type of assignment of coordinate into the specified Cytoarchitecture, as an output of the Anatomy Toolbox—HA: hard assignment, NHA: no hard assignment, NA: no assignment; Hem: hemisphere; Macroanatomy: assignment of the foci and to the Harvard‐Oxford microanatomical atlas; % macro: probability of the coordinate falling into the assigned region by the Harvard‐Oxford microanatomical atlas. AG, angular gyrus; AMYG, amygdala; CGa, cingulate gyrus, anterior; CGp, cingulate gyrus, posterior; COP, central opercular cortex; CRcr‐I, cerebellum crus I; CRcr‐II, cerebellum crus II; FMC, frontal medial cortex; FO, frontal operculum cortex; FOC, frontal orbital cortex; FP, frontal pole; HC, hippocampus; HG, Heschl's gyrus; IC, insular cortex; IFG POp, inferior frontal gyrus, pars opercularis; IFG PTr, inferior frontal gyrus, pars triangularis; IFGt, inferior frontal gyrus, temporooccipital; ITGp, inferior temporal gyrus, posterior; ITGt, inferior temporal gyrus, temporooccipital; JLC, juxtapositional lobule cortex; LOCi, lateral occipital cortex, inferior; LOCs, lateral occipital cortex, superior; MFG, middle frontal gyrus; MTGa, middle temporal gyrus, anterior; MTGp, middle temporal gyrus, posterior; MTGt, middle temporal gyrus, temporooccipital; OFC, occipital fusiform gyrus; OP, occipital pole; PAC, paracingulate gyrus; PC, precuneous cortex; PGp, parahippocampal gyrus, posterior; POC, parietal operculum cortex; PP, planum polare; PRG, precentral gyrus; PT, planum temporale; RC, right caudate; SFG, superior frontal gyrus; SGp, supramarginal gyrus, posterior; SPL, superior parietal lobule; STGa, superior temporal gyrus, anterior; STGp, superior temporal gyrus, posterior; STGs, superior temporal gyrus, superior; TFCp, temporal fusiform cortex, posterior; TOFC, temporal occipital fusiform cortex; TP, temporal pole.

**Table S6:** All activation clusters and local maxima for *Semantic Control* (Separate ALE Meta‐Analyses, Conjunction and Subtraction Analyses). Coordinates *(X*, *Y* *and* *Z)* are reported in the MNI coordinate system; Clust no: cluster number in the individual contrast; ALE: activation likelihood estimation values output from GingerALE, along with *p* and *Z* values; Cytoarchitecture: cytoarchitectonic information for foci assigned by the JuBrain Anatomy Toolbox (SPM), based on the Maximum Probability Map; % cyto: probability of the coordinate falling into the specified Cytoarchitecture, as an output of the Anatomy Toolbox; Assignment: type of assignment of coordinate into the specified Cytoarchitecture, as an output of the Anatomy Toolbox—HA: hard assignment, NHA: no hard assignment, NA: no assignment, Hem: hemisphere; Macroanatomy: assignment of the foci and to the Harvard‐Oxford microanatomical atlas; % macro: probability of the coordinate falling into the assigned region by the Harvard‐Oxford microanatomical atlas. AG, angular gyrus; AMYG, amygdala; CGa, cingulate gyrus, anterior; CGp, cingulate gyrus, posterior; COP, central opercular cortex; CRcr‐I, cerebellum crus I; CRcr‐II, cerebellum crus II; FMC, frontal medial cortex; FO, frontal operculum cortex; FOC, frontal orbital cortex; FP, frontal pole; HC, hippocampus; HG, Heschl's gyrus; IC, insular cortex; IFG POp, inferior frontal gyrus, pars opercularis; IFG PTr, inferior frontal gyrus, pars triangularis; IFGt, inferior frontal gyrus, temporooccipital; ITGp, inferior temporal gyrus, posterior; ITGt, inferior temporal gyrus, temporooccipital; JLC, juxtapositional lobule cortex; LOCi, lateral occipital cortex, inferior; LOCs, lateral occipital cortex, superior; MFG, middle frontal gyrus; MTGa, middle temporal gyrus, anterior; MTGp, middle temporal gyrus, posterior; MTGt, middle temporal gyrus, temporooccipital; OFC, occipital fusiform gyrus; OP, occipital pole; PAC, paracingulate gyrus; PC, precuneous cortex; PGp, parahippocampal gyrus, posterior; POC, parietal operculum cortex; PP, planum polare; PRG, precentral gyrus; PT, planum temporale; RC, right caudate; SFG, superior frontal gyrus; SGp, supramarginal gyrus, posterior; SPL, superior parietal lobule; STGa, superior temporal gyrus, anterior; STGp, superior temporal gyrus, posterior; STGs, superior temporal gyrus, superior; TFCp, temporal fusiform cortex, posterior; TOFC, temporal occipital fusiform cortex; TP, temporal pole.

**Table S7:** All activation clusters and local maxima for *Social* *Cognition* and *Verbal Semantic Cognition* (Separate ALE Meta‐Analyses, Conjunction and Subtraction Analyses). Coordinates *(* *X, Y* *and* *Z* *)* are reported in the MNI coordinate system; Clust no: cluster number in the individual contrast; ALE: activation likelihood estimation values output from GingerALE, along with *p* and *Z* values; Cytoarchitecture: cytoarchitectonic information for foci assigned by the JuBrain Anatomy Toolbox (SPM), based on the maximum probability map; % cyto: probability of the coordinate falling into the specified Cytoarchitecture, as an output of the Anatomy Toolbox; Assignment: type of assignment of coordinate into the specified Cytoarchitecture, as an output of the Anatomy Toolbox—HA: hard assignment, NHA: no hard assignment, NA: no assignment, Hem: hemisphere; Macroanatomy: Assignment of the foci and to the Harvard‐Oxford microanatomical atlas; % macro: probability of the coordinate falling into the assigned region by the Harvard‐Oxford microanatomical atlas. aCG: cingulate gyrus, anterior; AG, angular gyrus; AMYG, amygdala; COP, central opercular cortex; CRcr‐I, cerebellum crus I; CRcr‐II, cerebellum crus II; FMC, frontal medial cortex; FO, frontal operculum cortex; FOC, frontal orbital cortex; FP, frontal pole; HC, hippocampus; HG, Heschl's gyrus; IC, insular cortex; IFG POp, inferior frontal gyrus, pars opercularis; IFG PTr, inferior frontal gyrus, pars triangularis; IFGt, inferior frontal gyrus, temporooccipital; ITGp, inferior temporal gyrus, posterior; ITGt, inferior temporal gyrus, temporooccipital; JLC, juxtapositional lobule cortex; LOCi, lateral occipital cortex, inferior; LOCs, lateral occipital cortex, superior; MFG, middle frontal gyrus; MTGa, middle temporal gyrus, anterior; MTGp, middle temporal gyrus, posterior; MTGt, middle temporal gyrus, temporooccipital; OFC, occipital fusiform gyrus; OP, occipital pole; PAC, paracingulate gyrus; PC, precuneous cortex; pCG, cingulate gyrus, posterior; PGp, parahippocampal gyrus, posterior; POC, parietal operculum cortex; PP, planum polare; PRG, precentral gyrus; PT, planum temporale; RC, right caudate; SFG, superior frontal gyrus; SGp, supramarginal gyrus, posterior; SPL, superior parietal lobule; STGa, superior temporal gyrus, anterior; STGp, superior temporal gyrus, posterior; STGs, superior temporal gyrus, superior; TFCp, temporal fusiform cortex, posterior; TOFC, temporal occipital fusiform cortex; TP, temporal pole.

**Table S8:** All activation clusters and local maxima for *Verbal Semantic Cognition*, split by external task demands (Separate ALE Meta‐Analyses). Coordinates *(* *X, Y* *and* *Z)* are reported in the MNI coordinate system; Clust no: cluster number in the individual contrast; ALE: activation likelihood estimation values output from Ginger ALE, along with *p* and *Z* values; Cytoarchitecture: cytoarchitectonic information for foci assigned by the JuBrain Anatomy Toolbox (SPM), based on the maximum probability map; % cyto: probability of the coordinate falling into the specified Cytoarchitecture, as an output of the Anatomy Toolbox; Assignment: type of assignment of coordinate into the specified Cytoarchitecture, as an output of the Anatomy Toolbox—HA: hard assignment, NHA: no hard assignment, NA: no assignment, Hem: hemisphere; Macroanatomy: assignment of the foci and to the Harvard‐Oxford microanatomical atlas; % macro: probability of the coordinate falling into the assigned region by the Harvard‐Oxford microanatomical atlas. aCG, cingulate gyrus, anterior; AG, angular gyrus; AMYG, amygdala; COP, central opercular cortex; CRcr‐I, cerebellum crus I; CRcr‐II, cerebellum crus II; FMC, frontal medial cortex; FO, frontal operculum cortex; FOC, frontal orbital cortex; FP, frontal pole; HC, hippocampus; HG, Heschl's gyrus; IC, insular cortex; IFG POp, inferior frontal gyrus, pars opercularis; IFG PTr, inferior frontal gyrus, pars triangularis; IFGt, inferior frontal gyrus, temporooccipital; ITGp, inferior temporal gyrus, posterior; ITGt, inferior temporal gyrus, temporooccipital; JLC, juxtapositional lobule cortex; LOCi, lateral occipital cortex, inferior; LOCs, lateral occipital cortex, superior; MFG, middle frontal gyrus; MTGa, middle temporal gyrus, anterior; MTGp, middle temporal gyrus, posterior; MTGt, middle temporal gyrus, temporooccipital; OFC, occipital fusiform gyrus; OP, occipital pole; PAC, paracingulate gyrus; PC, precuneous cortex; pCG, cingulate gyrus, posterior; PGp, parahippocampal gyrus, posterior; POC, parietal operculum cortex; PP, planum polare; PRG, precentral gyrus; PT, planum temporale; RC, right caudate; SFG, superior frontal gyrus; SGp, supramarginal gyrus, posterior; SPL, superior parietal lobule; STGa, superior temporal gyrus, anterior; STGp, superior temporal gyrus, posterior; STGs, superior temporal gyrus, superior; TFCp, temporal fusiform cortex, posterior; TOFC, temporal occipital fusiform cortex; TP, temporal pole.

## Data Availability

All data is included in the Supporting Information (Tables [Link], [Link]). Only freely available toolboxes were used to process the data (available at http://www.brainmap.org/software.html#GingerALE). Masks of the results are available online at https://osf.io/nd8ye/?view_only=067a2cc490c2426781a0e2ad3b0c6073.
