# Supplementary figures and images for: The Role of the Right Language Network and the Multiple‐Demand Network in Verbal Semantics: Insights From an Activation Likelihood Estimation Meta‐Analysis of 561 Functional Neuroimaging Studies
Source: Hum Brain Mapp. 2025 Dec 20;46(18):e70415. doi: 10.1002/hbm.70415 (PMC12718395; doi:10.1002/hbm.70415)

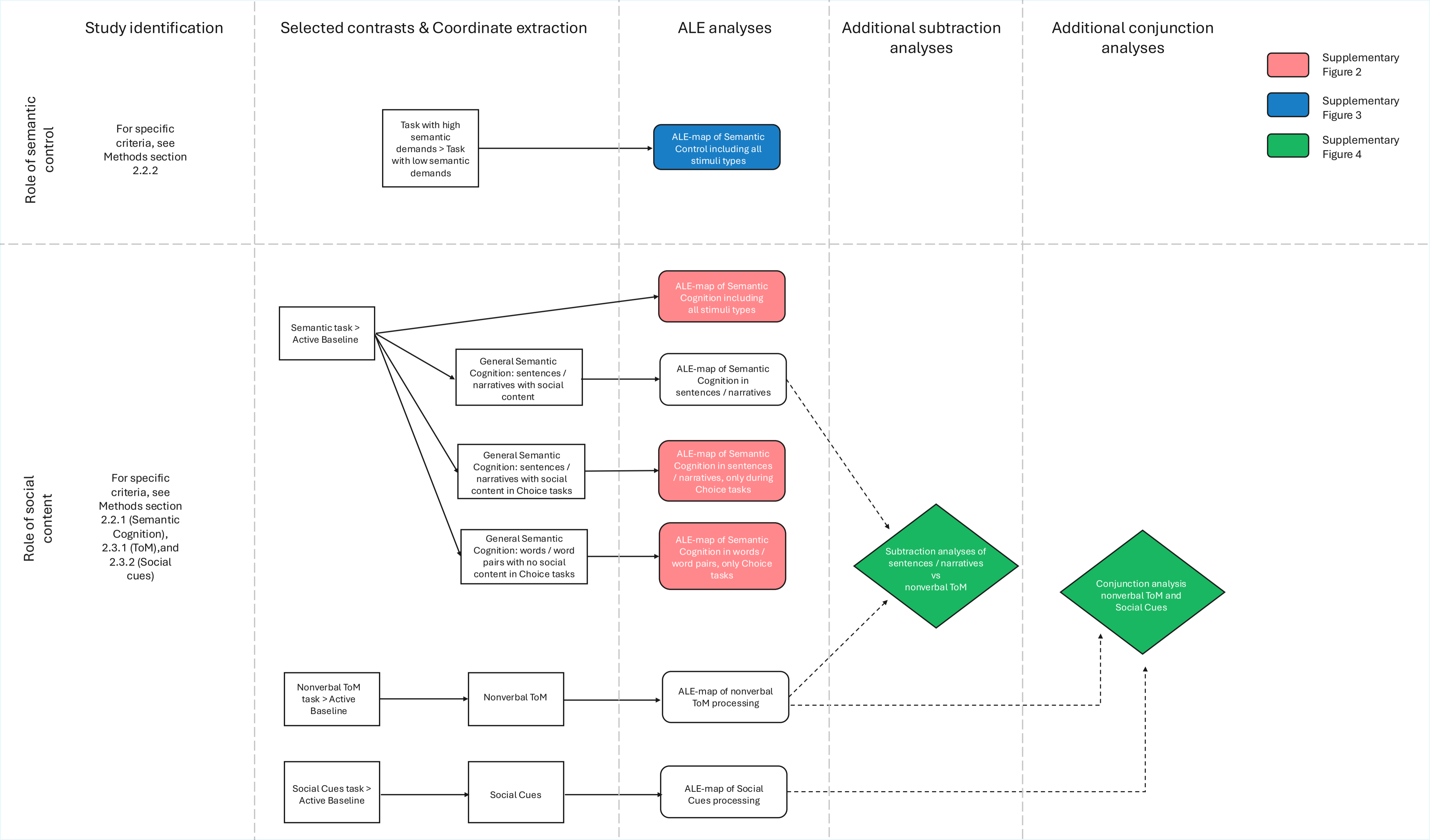

Supplement: Supplementary file 1 — Figure S1: The flowchart summarises the procedures for studies identification, coordinates extraction and key statistical analyses relative to the results reported in the Supporting Information; the analysis steps are mapped to corresponding Supplementary Figures via different colour codes. [file HBM-46-e70415-s004.pdf]

## (A) Verbal Semantic Cognition

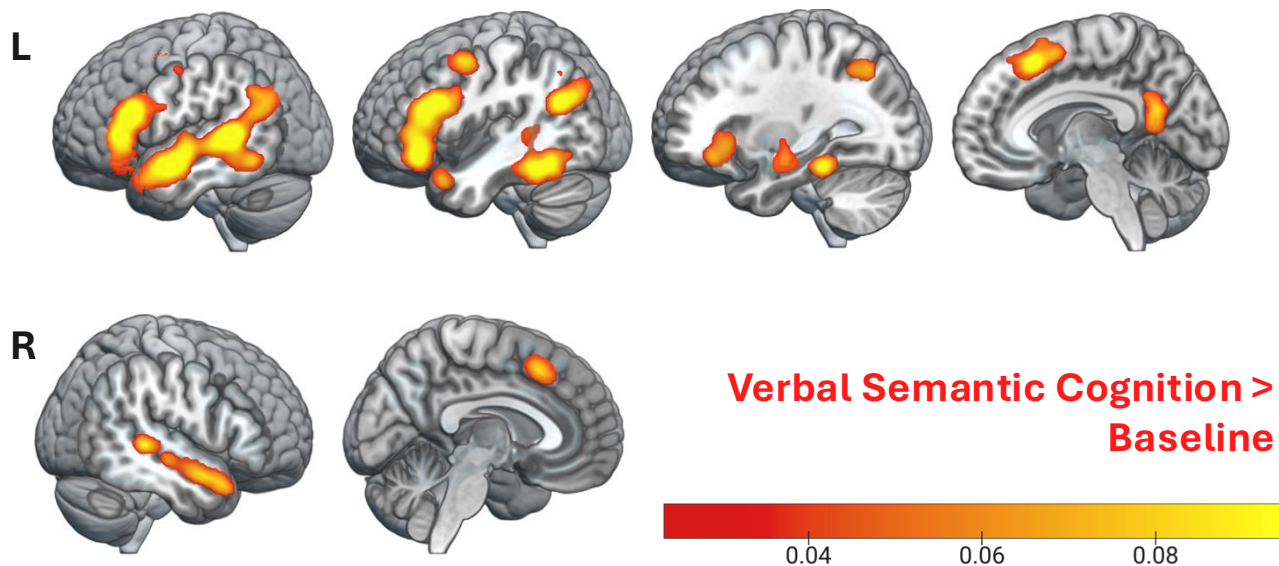

## (B) Verbal semantic cognition, choice tasks

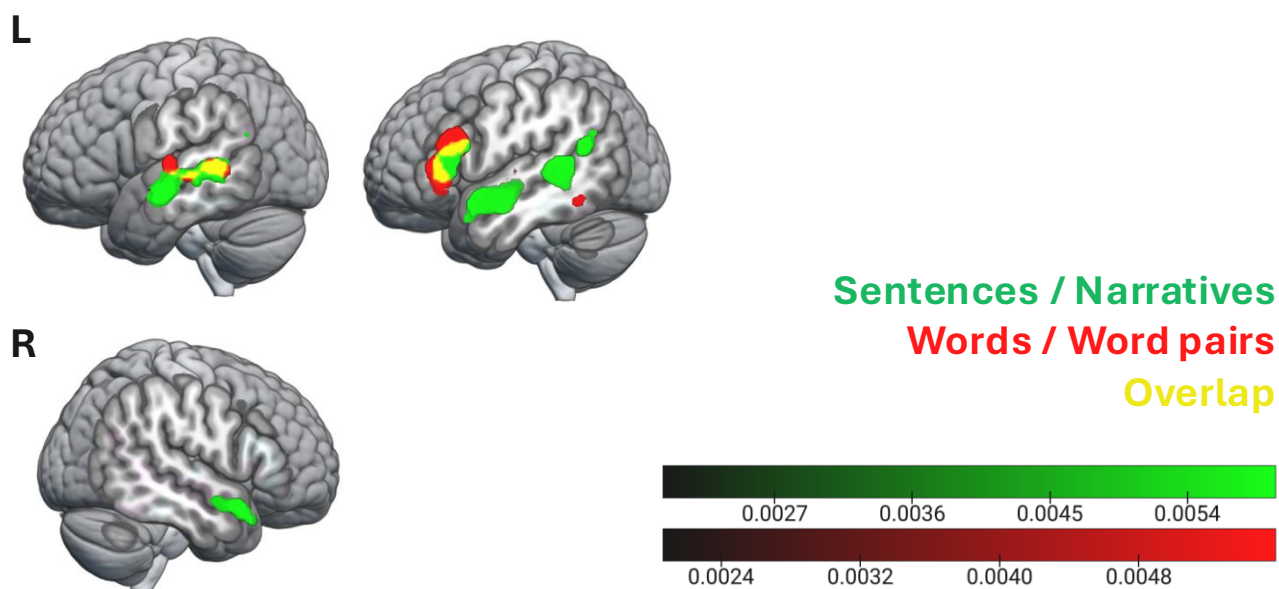

Supplement: Supplementary file 2 — Figure S2: General verbal semantic cognition. (A) Brain regions activated during all verbal semantic > non‐semantic (or less semantic) tasks. Colour bar: ALE‐values; cluster forming threshold: p < 0.001; cluster extent correction: family‐wise error (FWE) p < 0.001. (B) Brain regions activated during verbal semantic processing, only including tasks in which participants had to perform a choice, split by stimuli type (green: sentences/narratives, red: single words/word pairs, yellow: overlapping brain regions). Colour bar: ALE‐values, p < 0.001, cluster extent correction: FWE p < 0.001. [file HBM-46-e70415-s005.pdf]

# Verbal Semantic Control

L

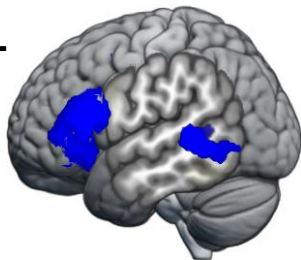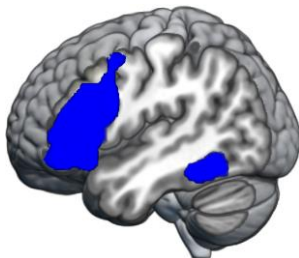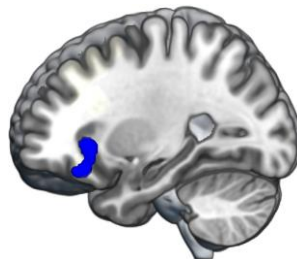

R

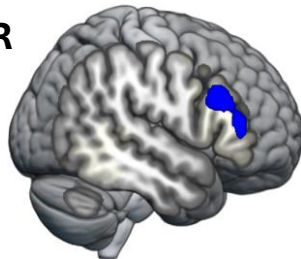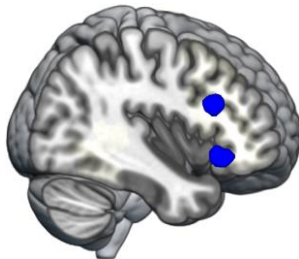

Verbal Semantic Hard >  
Verbal Semantic Easy

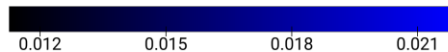

Supplement: Supplementary file 3 — Figure S3: Brain regions reliably activated during all hard versus easy semantic tasks or conditions. Colour bar: ALE‐values; cluster forming threshold: p < 0.001 (uncorrected); cluster extent correction: family‐wise error (FWE) p < 0.001. [file HBM-46-e70415-s009.pdf]

## (A) Social processing

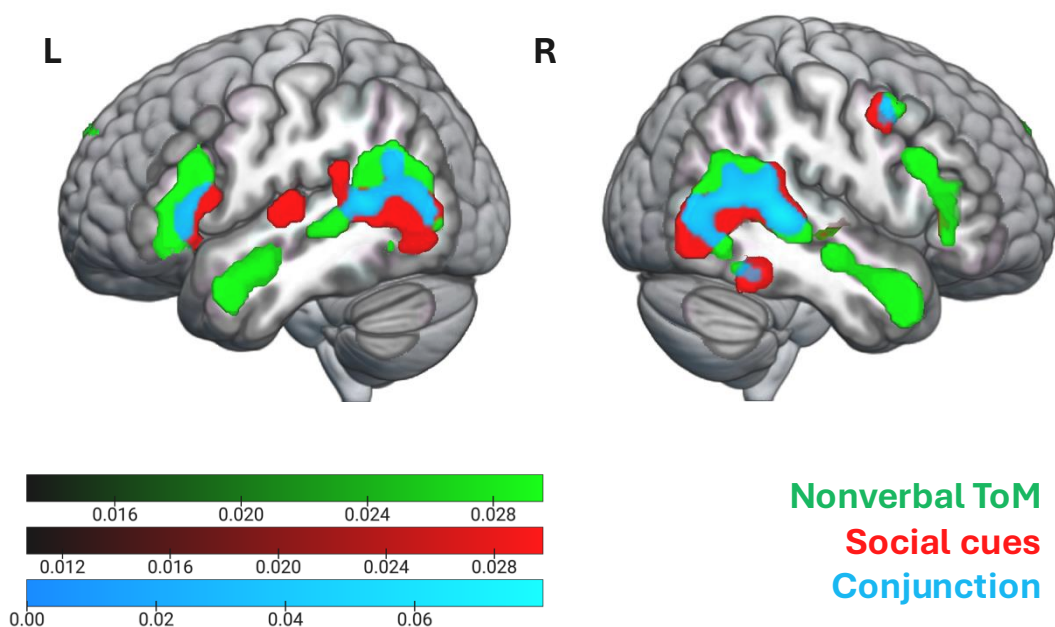

## (B) Non-verbal ToM and Sentences, subtraction analysis

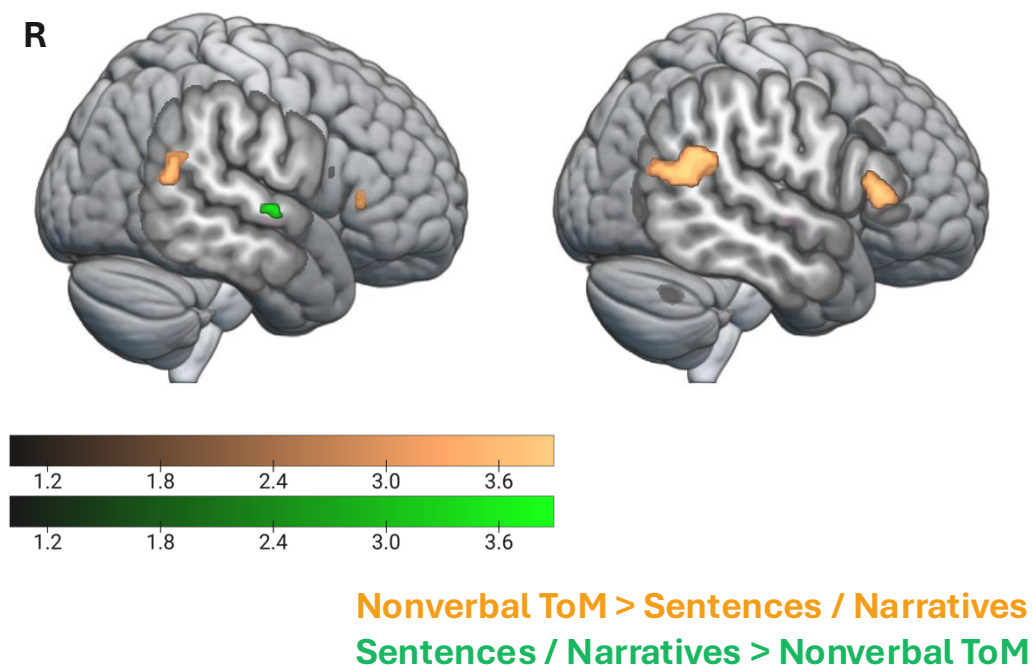

Supplement: Supplementary file 4 — Figure S4: Social processing. (A) Individual ALE meta‐analyses and conjunction analyses of nonverbal Theory of Mind processing (green), Social Cues processing (red) and their conjunction (blue). Colour bar: ALE‐values; cluster forming threshold: p < 0.001 (uncorrected); cluster extent correction: family‐wise error (FWE) p < 0.001. Conjunction: ALE‐values, p < 0.001, minimum cluster volume: 200 mm3. (B) Brain regions differentially engaged depending on task and stimulus type (orange: nonverbal ToM tasks versus sentences/narratives; green: sentences/narratives versus nonverbal ToM tasks), calculated via t‐test/subtraction analysis. Colour bar: Z‐scores, cluster forming threshold: p < 0.001 (uncorrected), minimum cluster volume: 200 mm3. [file HBM-46-e70415-s008.pdf]
