# Supplementary material for: The Role of the Right Language Network and the Multiple‐Demand Network in Verbal Semantics: Insights From an Activation Likelihood Estimation Meta‐Analysis of 561 Functional Neuroimaging Studies
Source: Hum Brain Mapp. 2025 Dec 20;46(18):e70415. doi: 10.1002/hbm.70415 (PMC12718395; doi:10.1002/hbm.70415)
Supplement: Supplementary file 6 — Table S5: All activation clusters and local maxima for Verbal Semantic Cognition. Coordinates (X, Y and Z ) are reported in the MNI coordinate system; Clust no: cluster number in the individual contrast; ALE: activation likelihood estimate values output from GingerALE, along with p and Z values; Cytoarchitecture: cytoarchitectonic information for foci assigned by the JuBrain Anatomy Toolbox (SPM), based on the Maximum Probability Map; % cyto: probability of the coordinate falling into the specified Cytoarchitecture, as an output of the Anatomy Toolbox; Assignment: type of assignment of coordinate into the specified Cytoarchitecture, as an output of the Anatomy Toolbox—HA: hard assignment, NHA: no hard assignment, NA: no assignment; Hem: hemisphere; Macroanatomy: assignment of the foci and to the Harvard‐Oxford microanatomical atlas; % macro: probability of the coordinate falling into the assigned region by the Harvard‐Oxford microanatomical atlas. AG, angular gyrus; AMYG, amygdala; CGa, cingulate gyrus, anterior; CGp, cingulate gyrus, posterior; COP, central opercular cortex; CRcr‐I, cerebellum crus I; CRcr‐II, cerebellum crus II; FMC, frontal medial cortex; FO, frontal operculum cortex; FOC, frontal orbital cortex; FP, frontal pole; HC, hippocampus; HG, Heschl's gyrus; IC, insular cortex; IFG POp, inferior frontal gyrus, pars opercularis; IFG PTr, inferior frontal gyrus, pars triangularis; IFGt, inferior frontal gyrus, temporooccipital; ITGp, inferior temporal gyrus, posterior; ITGt, inferior temporal gyrus, temporooccipital; JLC, juxtapositional lobule cortex; LOCi, lateral occipital cortex, inferior; LOCs, lateral occipital cortex, superior; MFG, middle frontal gyrus; MTGa, middle temporal gyrus, anterior; MTGp, middle temporal gyrus, posterior; MTGt, middle temporal gyrus, temporooccipital; OFC, occipital fusiform gyrus; OP, occipital pole; PAC, paracingulate gyrus; PC, precuneous cortex; PGp, parahippocampal gyrus, posterior; POC, parietal operculum cortex; PP, [file HBM-46-e70415-s001.docx]

| **Clust no** | **Size (mm^3^**) | **X** | **Y** | **Z** | **ALE** | **P** | **Z** | **Cytoarchitecture** | **% cyto** | **Assignment** | **Hem.** | **Macroanatomy** | **% macro** |
| --- | --- | --- | --- | --- | --- | --- | --- | --- | --- | --- | --- | --- | --- |
| **Semantic (all) > Non-semantic or less semantic baseline** | | | | | | | | | | | | | |
| *ALE-analysis, cluster forming threshold: p < .001; cluster extent correction: FWE p < .001* | | | | | | | | | | | | | |
| 1 | 41416 | -54 | -40 | 2 | 0.240 | - | - | - | - | NA | Left | MTGp | 26 |
| 1 | 41416 | -56 | -4 | -14 | 0.173 | - | - | Area TE 5 | 71 | HA | Left | STGa | 41 |
| 1 | 41416 | -40 | -44 | -20 | 0.148 | - | - | Area FG4 | 72 | HA | Left | TFCp | 32 |
| 1 | 41416 | -44 | -60 | 24 | 0.144 | - | - | Area PGp (IPL) | 4 | NHA | Left | AG | 50 |
| 1 | 41416 | -48 | 14 | -26 | 0.123 | - | - | - | - | NA | Left | TP | 59 |
| 1 | 41416 | -32 | -36 | -18 | 0.114 | - | - | Area FG3 | 11 | NHA | Left | TFCp | 65 |
| 2 | 23488 | -50 | 24 | 14 | 0.182 | - | - | Area 45 | 33 | HA | Left | IFG PTr | 36 |
| 2 | 23488 | -50 | 28 | 0 | 0.165 | - | - | Area OP9 | 67 | HA | Left | IFG PTr | 42 |
| 2 | 23488 | -46 | 30 | -10 | 0.134 | - | - | Area OP9 | 12 | NHA | Left | FOC | 43 |
| 3 | 7480 | -4 | 20 | 48 | 0.148 | - | - | Area 6mr / preSMA | 6 | NHA | Left | PAC | 49 |
| 3 | 7480 | -6 | 8 | 54 | 0.083 | - | - | Area 6mr / preSMA | 84 | HA | Left | JLC | 37 |
| 4 | 6624 | 56 | 0 | -16 | 0.108 | - | - | Area TE 5 | 64 | HA | Right | STGa | 47 |
| 4 | 6624 | 50 | 14 | -24 | 0.083 | - | - | Area TE 5 | 2 | NHA | Right | TP | 81 |
| 4 | 6624 | 52 | -18 | -10 | 0.063 | - | - | Area TE 5 | 71 | HA | Right | MTGp | 51 |
| 5 | 3304 | -44 | 0 | 48 | 0.093 | - | - | - | - | NA | Left | PRG | 44 |
| 6 | 3184 | 36 | 24 | -6 | 0.083 | - | - | Area Id7 | 17 | NHA | Right | FOC | 65 |
| 6 | 3184 | 34 | 38 | -10 | 0.065 | - | - | Area Fo7 | 59 | HA | Right | FP | 61 |
| 7 | 2936 | 56 | -32 | 0 | 0.098 | - | - | Area TE 4 | 16 | NHA | Right | STGp | 31 |
| 7 | 2936 | 56 | -32 | 0 | 0.098 | - | - | Area TE 5 | 20 | NHA | Right | STGp | 31 |
| 8 | 2928 | -22 | -10 | -14 | 0.104 | - | - | Amygdala (VTM) | 29 | HA | Left | AMYG | 92 |
| 9 | 2688 | -4 | -58 | 18 | 0.093 | - | - | - | - | NA | Left | PC | 68 |
| 10 | 2552 | -32 | -64 | 40 | 0.077 | - | - | Area hIP6 (IPS) | 39 | HA | Left | LOCs | 54 |
|  |  |  |  |  |  |  |  |  |  |  |  |  |  |
| **Sentences/Narratives > Non-semantic or less semantic baseline** | | | | | | | | | | | | | |
| *ALE-analysis, cluster forming threshold: p < .001; cluster extent correction: FWE p < .001* | | | | | | | | | | | | | |
| 1 | 25056 | -52 | -40 | 4 | 0.174 | - | - | - | - | NA | Left | - | - |
| 1 | 25056 | -56 | -6 | -14 | 0.162 | - | - | Area TE 5 | 69 | HA | Left | MTGa | 38 |
| 1 | 25056 | -48 | 14 | -26 | 0.094 | - | - | - | - | NA | Left | TP | 59 |
| 1 | 25056 | -44 | -60 | 24 | 0.091 | - | - | Area PGp (IPL) | 4 | NHA | Left | AG | 50 |
| 1 | 25056 | -54 | -58 | 28 | 0.058 | - | - | Area PGa (IPL) | 57 | HA | Left | AG | 58 |
| 2 | 12704 | -54 | 22 | 14 | 0.114 | - | - | Area 45 | 40 | HA | Left | IFG PTr | 43 |
| 2 | 12704 | -50 | 28 | 2 | 0.105 | - | - | Area OP9 | 67 | HA | Left | IFG PTr | 49 |
| 3 | 9368 | 56 | 0 | -18 | 0.084 | - | - | Area TE 5 | 79 | HA | Right | STGa | 35 |
| 3 | 9368 | 52 | -34 | 0 | 0.069 | - | - | Area TE 5 | 25 | NHA | Right | MTGp | 33 |
| 3 | 9368 | 50 | 14 | -24 | 0.068 | - | - | Area TE 5 | 2 | NHA | Right | TP | 81 |
| 3 | 9368 | 52 | -18 | -8 | 0.054 | - | - | Area TE 5 | 57 | HA | Right | MTGp | 32 |
| 3 | 9368 | 64 | -32 | -2 | 0.046 | - | - | Area TE 5 | 25 | NHA | Right | MTGp | 56 |
| 4 | 3608 | -6 | 8 | 54 | 0.068 | - | - | Area 6mr / preSMA | 84 | HA | Left | JLC | 37 |
| 4 | 3608 | 6 | 18 | 44 | 0.050 | - | - | - | - | NA | Right | PAC | 62 |
| 4 | 3608 | -8 | 14 | 62 | 0.048 | - | - | Area 6mr / preSMA | 68 | HA | Left | - | - |
| 5 | 3328 | -40 | -46 | -20 | 0.087 | - | - | Area FG4 | 72 | HA | Left | TOFC | 33 |
| 5 | 3328 | -30 | -36 | -18 | 0.047 | - | - | Area FG3 | 10 | NHA | Left | TFCp | 64 |
| 6 | 2680 | -44 | 0 | 50 | 0.065 | - | - | - | - | NA | Left | PRG | 31 |
| 6 | 2680 | -40 | 12 | 50 | 0.048 | - | - | - | - | NA | Left | MFG | 51 |
| 6 | 2680 | -42 | 22 | 42 | 0.037 | - | - | - | - | NA | Left | MFG | 67 |
|  |  |  |  |  |  |  |  |  |  |  |  |  |  |
| **Single-Words/Word-Pairs > Non-semantic or less semantic baseline** | | | | | | | | | | | | | |
| *ALE-analysis, cluster forming threshold: p < .001; cluster extent correction: FWE p < .001* | | | | | | | | | | | | | |
| 1 | 18616 | -56 | -36 | 0 | 0.096 | - | - | Area TE 4 | 7 | NHA | Left | MTGp | 38 |
| 1 | 18616 | -48 | -54 | -14 | 0.095 | - | - | Area FG4 | 58 | HA | Left | ITGt | 57 |
| 1 | 18616 | -40 | -42 | -22 | 0.077 | - | - | Area FG4 | 71 | HA | Left | TFCp | 52 |
| 1 | 18616 | -34 | -34 | -20 | 0.076 | - | - | Area FG3 | 12 | NHA | Left | TFCp | 64 |
| 1 | 18616 | -58 | -14 | -2 | 0.056 | - | - | Area TE 4 | 62 | HA | Left | - | - |
| 1 | 18616 | -56 | -42 | -12 | 0.050 | - | - | - | - | NA | Left | - | - |
| 2 | 17744 | -48 | 24 | 18 | 0.118 | - | - | Area 45 | 14 | NHA | Left | IFG PTr | 25 |
| 2 | 17744 | -46 | 30 | -10 | 0.083 | - | - | Area OP9 | 12 | NHA | Left | FOC | 43 |
| 2 | 17744 | -36 | 32 | -14 | 0.071 | - | - | Area Fo7 | 14 | NHA | Left | FOC | 48 |
| 2 | 17744 | -40 | 4 | 26 | 0.054 | - | - | Area 44 | 14 | NHA | Left | - | - |
| 2 | 17744 | -32 | 24 | -4 | 0.043 | - | - | Area Id7 | 32 | NHA | Left | IC | 41 |
| 3 | 6624 | -46 | -68 | 26 | 0.089 | - | - | Area PGp (IPL) | 45 | HA | Left | LOCs | 66 |
| 3 | 6624 | -32 | -64 | 40 | 0.065 | - | - | Area hIP6 (IPS) | 39 | HA | Left | LOCs | 54 |
| 3 | 6624 | -34 | -52 | 44 | 0.042 | - | - | Area hIP3 (IPS) | 43 | HA | Left | SPL | 31 |
| 4 | 5016 | -4 | 20 | 48 | 0.109 | - | - | Area 6mr / preSMA | 6 | NHA | Left | PAC | 49 |
| 5 | 2792 | 36 | 24 | -6 | 0.067 | - | - | Area Id7 | 17 | NHA | Right | FOC | 65 |
| 5 | 2792 | 34 | 38 | -8 | 0.051 | - | - | Area Fo7 | 54 | HA | Right | - | - |
|  |  |  |  |  |  |  |  |  |  |  |  |  |  |
| **Sentences /Narratives > Single-Words/Word-Pairs** | | | | | | | | | | | | | |
| *Subtraction analysis, p < .001; minimum cluster volume: 200 mm3* | | | | | | | | | | | | | |
| 1 | 4560 | -56 | -7 | -13 | - | <.001 | 3.891 | Area TE 5 | 67 | HA | Left | MTGa | 31 |
| 1 | 4560 | -51 | 6 | -27 | - | <.001 | 3.432 | Area TE 5 | 42 | NHA | Left | TP | 64 |
| 1 | 4560 | -50 | 4 | -18 | - | <.001 | 3.540 | Area TE 5 | 47 | HA | Left | TP | 40 |
| 2 | 1248 | 58 | 1 | -22 | - | <.001 | 3.891 | Area TE 5 | 76 | HA | Right | MTGa | 51 |
| 2 | 1248 | 51 | 8 | -25 | - | <.001 | 3.121 | Area TE 5 | 59 | HA | Right | TP | 70 |
| 2 | 1248 | 51 | 11 | -26 | - | <.001 | 3.540 | Area TE 5 | 21 | NHA | Right | TP | 84 |
| 2 | 1248 | 48 | 18 | -20 | - | <.001 | 3.291 | - | - | NA | Right | TP | 72 |
| 3 | 912 | -51 | -51 | 12 | - | <.001 | 3.891 | - | - | NA | Left | AG | 25 |
| 3 | 912 | -47 | -53 | 15 | - | <.001 | 3.719 | - | - | NA | Left | AG | 27 |
| 3 | 912 | -48 | -42 | 4 | - | <.001 | 3.432 | - | - | NA | Left | - | - |
| 4 | 552 | -57 | 23 | 12 | - | <.001 | 3.891 | Area 45 | 55 | HA | Left | IFG PTr | 49 |
| 5 | 224 | 48 | -32 | -6 | - | <.001 | 3.719 | Area TE 4 | 16 | NHA | Right | MTGp | 48 |
| 5 | 224 | 52 | -34 | -6 | - | <.001 | 3.540 | - | - | NA | Right | - | - |
|  |  |  |  |  |  |  |  |  |  |  |  |  |  |
| **Single-Words/Word-Pairs > Sentences/Narratives** | | | | | | | | | | | | | |
| *Subtraction analysis, p < .001; minimum cluster volume: 200 mm3* | | | | | | | | | | | | | |
|  |  |  |  |  |  |  |  |  |  |  |  |  |  |
| 1 | 392 | -46 | -75 | 27 | - | <.000 | 3.891 | Area PGp (IPL) | 81 | HA | Left | LOCs | 87 |
| 1 | 392 | -46 | -76 | 32 | - | <.001 | 3.719 | Area PGp (IPL) | 79 | HA | Left | LOCs | 92 |
| 1 | 392 | -42 | -80 | 30 | - | <.001 | 3.540 | Area PGp (IPL) | 56 | HA | Left | LOCs | 85 |
| 2 | 264 | -47 | -48 | -12 | - | <.001 | 3.719 | Area FG4 | 85 | HA | Left | ITGt | 34 |
| 2 | 264 | -50 | -41 | -15 | - | <.001 | 3.432 | Area FG4 | 26 | NHA | Left | - | - |
| 3 | 264 | -3 | 31 | 38 | - | <.001 | 3.891 | - | - | NA | Left | PAC | 75 |
|  |  |  |  |  |  |  |  |  |  |  |  |  |  |
| **Auditory semantic (all) > Auditory non-semantic or less semantic baseline** | | | | | | | | | | | | | |
| *ALE-analysis, cluster forming threshold: p < .001; cluster extent correction: FWE p < .001* | | | | | | | | | | | | | |
| 1 | 19880 | -58 | -12 | -4 | 0.104 | - | - | Area TE 4 | 77 | HA | Left | STGa | 28 |
| 1 | 19880 | -56 | -6 | -12 | 0.078 | - | - | Area TE 5 | 62 | HA | Left | STGa | 35 |
| 1 | 19880 | -52 | 8 | -22 | 0.069 | - | - | Area TE 5 | 36 | NHA | Left | TP | 86 |
| 1 | 19880 | -52 | -40 | 4 | 0.065 | - | - | - | - | NA | Left | - | - |
| 1 | 19880 | -46 | -60 | 22 | 0.062 | - | - | Area PGp (IPL) | 11 | NHA | Left | AG | 48 |
| 1 | 19880 | -60 | -32 | 4 | 0.048 | - | - | Area TE 4 | 2 | NHA | Left | STGp | 31 |
| 2 | 14456 | 60 | -6 | -8 | 0.085 | - | - | Area TE 3 | 39 | HA | Right | STGa | 36 |
| 2 | 14456 | 58 | -32 | 2 | 0.072 | - | - | Area TE 4 | 18 | NHA | Right | STGp | 32 |
| 2 | 14456 | 52 | 4 | -20 | 0.065 | - | - | Area TE 5 | 73 | HA | Right | STGa | 35 |
| 2 | 14456 | 52 | -18 | -8 | 0.046 | - | - | Area TE 5 | 57 | HA | Right | MTGp | 32 |
| 2 | 14456 | 64 | -18 | -2 | 0.043 | - | - | Area TE 4 | 41 | HA | Right | - | - |
| 2 | 14456 | 50 | -18 | 4 | 0.038 | - | - | Area TE 1.1 | 2 | NHA | Right | - | - |
| 3 | 7616 | -50 | 30 | 0 | 0.068 | - | - | Area OP9 | 52 | HA | Left | IFG PTr | 51 |
| 3 | 7616 | -46 | 16 | 22 | 0.053 | - | - | Area 44 | 23 | NHA | Left | IFG POp | 51 |
| 3 | 7616 | -52 | 24 | 14 | 0.050 | - | - | Area 45 | 42 | HA | Left | IFG PTr | 43 |
| 3 | 7616 | -40 | 32 | -12 | 0.049 | - | - | Area Fo7 | 23 | NHA | Left | FOC | 45 |
| 4 | 2416 | -36 | -34 | -18 | 0.050 | - | - | CA1 (Hippocampus) | 26 | NHA | Left | TFCp | 55 |
| 4 | 2416 | -40 | -42 | -18 | 0.044 | - | - | Area FG4 | 56 | HA | Left | TFCp | 37 |
|  |  |  |  |  |  |  |  |  |  |  |  |  |  |
| **Visual semantic (all) > Visual non-semantic or less semantic baseline** | | | | | | | | | | | | | |
| *ALE-analysis, cluster forming threshold: p < .001; cluster extent correction: FWE p < .001* | | | | | | | | | | | | | |
| 1 | 21848 | -50 | 24 | 14 | 0.136 | - | - | Area 45 | 33 | HA | Left | IFG PTr | 36 |
| 1 | 21848 | -52 | 28 | 4 | 0.108 | - | - | Area OP9 | 55 | HA | Left | IFG PTr | 58 |
| 1 | 21848 | -46 | 30 | -10 | 0.097 | - | - | Area OP9 | 12 | NHA | Left | FOC | 43 |
| 1 | 21848 | -48 | 16 | -26 | 0.084 | - | - | - | - | NA | Left | TP | 65 |
| 1 | 21848 | -36 | 32 | -14 | 0.079 | - | - | Area Fo7 | 14 | NHA | Left | FOC | 48 |
| 1 | 21848 | -44 | 6 | 26 | 0.056 | - | - | Area 44 | 51 | HA | Left | IFG POp | 34 |
| 1 | 21848 | -38 | 18 | 2 | 0.047 | - | - | Area Id6 | 54 | HA | Left | FO | 40 |
| 2 | 21072 | -54 | -40 | 2 | 0.192 | - | - | - | - | NA | Left | MTGp | 26 |
| 2 | 21072 | -44 | -54 | -16 | 0.112 | - | - | Area FG4 | 78 | HA | Left | TOFC | 42 |
| 2 | 21072 | -40 | -46 | -20 | 0.110 | - | - | Area FG4 | 72 | HA | Left | TOFC | 33 |
| 2 | 21072 | -22 | -10 | -14 | 0.085 | - | - | Amygdala (VTM) | 29 | HA | Left | AMYG | 92 |
| 2 | 21072 | -32 | -36 | -20 | 0.069 | - | - | Area FG3 | 20 | NHA | Left | TFCp | 66 |
| 3 | 7936 | -4 | 20 | 48 | 0.106 | - | - | Area 6mr / preSMA | 6 | NHA | Left | PAC | 49 |
| 3 | 7936 | -10 | 46 | 40 | 0.060 | - | - | - | - | NA | Left | - | - |
| 3 | 7936 | -10 | 52 | 38 | 0.059 | - | - | - | - | NA | Left | FP | 70 |
| 3 | 7936 | -6 | 6 | 54 | 0.059 | - | - | - | - | NA | Left | JLC | 60 |
| 3 | 7936 | -8 | 16 | 60 | 0.048 | - | - | Area 6mr / preSMA | 60 | HA | Left | - | - |
| 4 | 4264 | -56 | -2 | -16 | 0.115 | - | - | Area TE 5 | 78 | HA | Left | MTGa | 42 |
| 5 | 4264 | -42 | -58 | 24 | 0.085 | - | - | Area PGa (IPL) | 0 | NHA | Left | AG | 53 |
| 5 | 3920 | -46 | -66 | 26 | 0.082 | - | - | Area PGp (IPL) | 35 | NHA | Left | LOCs | 54 |
| 6 | 3304 | -44 | -2 | 48 | 0.074 | - | - | - | - | NA | Left | PRG | 44 |
| 7 | 2376 | 36 | 24 | -6 | 0.060 | - | - | Area Id7 | 17 | NHA | Right | FOC | 65 |
| 7 | 2376 | 36 | 36 | -10 | 0.052 | - | - | Area Fo7 | 45 | HA | Right | FP | 50 |
| 8 | 2120 | -2 | -58 | 18 | 0.080 | - | - | - | - | NA | Left | PC | 73 |
| 8 | 2120 | -12 | -50 | 2 | 0.044 | - | - | - | - | NA | Left | CGp | 41 |
| 9 | 1992 | -30 | -58 | 44 | 0.058 | - | - | Area hIP3 (IPS) | 66 | HA | Left | SPL | 30 |
|  |  |  |  |  |  |  |  |  |  |  |  |  |  |
| **Auditory Sentences/Narratives > Auditory non-semantic or less semantic baseline** | | | | | | | | | | | | | |
| *ALE-analysis, cluster forming threshold: p < .001; cluster extent correction: FWE p < .001* | | | | | | | | | | | | | |
| 1 | 16224 | -58 | -12 | -6 | 0.077 | - | - | Area TE 4 | 78 | HA | Left | STGa | 22 |
| 1 | 16224 | -56 | -6 | -12 | 0.072 | - | - | Area TE 5 | 62 | HA | Left | STGa | 35 |
| 1 | 16224 | -52 | 8 | -22 | 0.066 | - | - | Area TE 5 | 36 | NHA | Left | TP | 86 |
| 1 | 16224 | -52 | -40 | 4 | 0.058 | - | - | - | - | NA | Left | STGp | 18 |
| 1 | 16224 | -64 | -22 | 0 | 0.043 | - | - | Area TE 4 | 37 | HA | Left | STGp | 41 |
| 1 | 16224 | -44 | -60 | 24 | 0.039 | - | - | Area PGp (IPL) | 4 | NHA | Left | AG | 50 |
| 1 | 16224 | -52 | -52 | 16 | 0.039 | - | - | - | - | NA | Left | AG | 35 |
| 1 | 16224 | -62 | -52 | 0 | 0.028 | - | - | - | - | NA | Left | MTGt | 68 |
| 2 | 9648 | 58 | -6 | -10 | 0.063 | - | - | Area TE 4 | 46 | HA | Right | STGa | 32 |
| 2 | 9648 | 52 | 4 | -20 | 0.057 | - | - | Area TE 5 | 73 | HA | Right | STGa | 35 |
| 2 | 9648 | 50 | 14 | -24 | 0.047 | - | - | Area TE 5 | 2 | NHA | Right | TP | 81 |
| 2 | 9648 | 52 | -34 | 0 | 0.045 | - | - | Area TE 5 | 25 | NHA | Right | MTGp | 33 |
| 2 | 9648 | 52 | -18 | -8 | 0.044 | - | - | Area TE 5 | 57 | HA | Right | MTGp | 32 |
| 2 | 9648 | 64 | -18 | -10 | 0.027 | - | - | Area TE 5 | 57 | HA | Right | MTGp | 57 |
| 2 | 9648 | 66 | -24 | 4 | 0.025 | - | - | Area TE 3 | 39 | HA | Right | STGp | 38 |
| 2 | 9648 | 52 | -20 | 2 | 0.023 | - | - | Area TE 4 | 7 | NHA | Right | - | - |
| 3 | 5520 | -50 | 28 | 0 | 0.053 | - | - | Area OP9 | 67 | HA | Left | IFG PTr | 42 |
| 3 | 5520 | -44 | 14 | 22 | 0.044 | - | - | Area 44 | 23 | NHA | Left | - | - |
| 3 | 5520 | -54 | 24 | 14 | 0.043 | - | - | Area 45 | 55 | HA | Left | IFG PTr | 51 |
| 3 | 5520 | -42 | 32 | -14 | 0.032 | - | - | Area Fo7 | 52 | HA | Left | FOC | 49 |
|  |  |  |  |  |  |  |  |  |  |  |  |  |  |
| **Auditory Single-Words/Word-Pairs > Auditory non-semantic or less semantic baseline** | | | | | | | | | | | | | |
| *ALE-analysis, cluster forming threshold: p < .001; cluster extent correction: FWE p < .001* | | | | | | | | | | | | | |
| 1 | 3440 | -60 | -12 | 2 | 0.045 | - | - | Area TE 3 | 32 | HA | Left | PT | 25 |
| 1 | 3440 | -62 | -30 | 2 | 0.024 | - | - | Area TE 4 | 16 | NHA | Left | STGp | 30 |
| 2 | 1872 | 58 | -32 | 2 | 0.044 | - | - | Area TE 4 | 18 | NHA | Right | STGp | 32 |
| 3 | 1784 | -46 | 34 | 4 | 0.037 | - | - | Area OP9 | 36 | NHA | Left | IFG PTr | 31 |
| 3 | 1784 | -48 | 22 | 20 | 0.024 | - | - | Area 45 | 9 | NHA | Left | IFG POp | 25 |
| 3 | 1784 | -48 | 26 | 18 | 0.023 | - | - | Area 45 | 24 | NHA | Left | IFG PTr | 33 |
| 3 | 1784 | -42 | 32 | -8 | 0.021 | - | - | Area OP9 | 6 | NHA | Left | - | - |
| 4 | 1504 | 60 | -4 | -4 | 0.042 | - | - | Area TE 3 | 42 | HA | Right | STGa | 43 |
| 5 | 1288 | -36 | -32 | -18 | 0.036 | - | - | CA1 (Hippocampus) | 36 | NHA | Left | TFCp | 60 |
| 5 | 1288 | -28 | -38 | -20 | 0.026 | - | - | Area FG3 | 39 | NHA | Left | TFCp | 72 |
|  |  |  |  |  |  |  |  |  |  |  |  |  |  |
| **Visual Sentences/Narratives > Visual non-semantic or less semantic baseline** | | | | | | | | | | | | | |
| *ALE-analysis, cluster forming threshold: p < .001; cluster extent correction: FWE p < .001* | | | | | | | | | | | | | |
| 1 | 7240 | -54 | 22 | 14 | 0.071 | - | - | Area 45 | 40 | HA | Left | IFG PTr | 43 |
| 1 | 7240 | -50 | 28 | 8 | 0.068 | - | - | Area OP9 | 71 | HA | Left | IFG PTr | 54 |
| 1 | 7240 | -38 | 28 | -6 | 0.037 | - | - | Area OP9 | 14 | NHA | Left | FOC | 54 |
| 2 | 5224 | -54 | -40 | 4 | 0.123 | - | - | - | - | NA | Left | - | - |
| 3 | 4464 | -56 | -4 | -14 | 0.100 | - | - | Area TE 5 | 71 | HA | Left | STGa | 41 |
| 4 | 2344 | -42 | -54 | -20 | 0.070 | - | - | Area FG4 | 63 | HA | Left | TOFC | 58 |
| 5 | 2120 | -44 | -58 | 24 | 0.054 | - | - | Area PGa (IPL) | 1 | NHA | Left | AG | 59 |
| 5 | 2120 | -56 | -58 | 28 | 0.040 | - | - | Area PGa (IPL) | 51 | HA | Left | AG | 55 |
| 6 | 1848 | 6 | 16 | 44 | 0.046 | - | - | Area 6mr / preSMA | 0 | NHA | Right | PAC | 66 |
| 6 | 1848 | -6 | 6 | 54 | 0.043 | - | - | - | - | NA | Left | JLC | 60 |
| 6 | 1848 | -4 | 18 | 48 | 0.032 | - | - | Area 6mr / preSMA | 8 | NHA | Left | PAC | 61 |
| 7 | 1648 | -42 | -2 | 46 | 0.046 | - | - | - | - | NA | Left | PRG | 37 |
| 7 | 1648 | -42 | 14 | 48 | 0.035 | - | - | - | - | NA | Left | MFG | 59 |
|  |  |  |  |  |  |  |  |  |  |  |  |  |  |
| **Visual Single-Words/Word-Pairs > Visual non-semantic or less semantic baseline** | | | | | | | | | | | | | |
| *ALE-analysis, cluster forming threshold: p < .001; cluster extent correction: FWE p < .001* | | | | | | | | | | | | | |
| 1 | 15520 | -48 | 24 | 16 | 0.092 |  |  | Area 45 | 14 | NHA | Left | - | - |
| 1 | 15520 | -46 | 30 | -12 | 0.075 |  |  | Area Fo7 | 3 | NHA | Left | FOC | 62 |
| 1 | 15520 | -50 | 28 | -2 | 0.061 |  |  | Area OP9 | 67 | HA | Left | IFG PTr | 43 |
| 1 | 15520 | -34 | 34 | -14 | 0.057 |  |  | Area Fo7 | 34 | NHA | Left | FOC | 49 |
| 1 | 15520 | -40 | 18 | 4 | 0.041 |  |  | Area OP8 | 39 | HA | Left | FO | 72 |
| 1 | 15520 | -48 | 6 | 22 | 0.040 |  |  | Area 44 | 53 | HA | Left | PRG | 37 |
| 1 | 15520 | -44 | 4 | 24 | 0.040 |  |  | Area 44 | 30 | NHA | Left | PRG | 32 |
| 2 | 15080 | -56 | -36 | 0 | 0.089 |  |  | Area TE 4 | 7 | NHA | Left | MTGp | 38 |
| 2 | 15080 | -48 | -54 | -14 | 0.084 |  |  | Area FG4 | 58 | HA | Left | ITGt | 57 |
| 2 | 15080 | -40 | -42 | -22 | 0.065 |  |  | Area FG4 | 71 | HA | Left | TFCp | 52 |
| 2 | 15080 | -32 | -36 | -20 | 0.050 |  |  | Area FG3 | 20 | NHA | Left | TFCp | 66 |
| 2 | 15080 | -56 | -40 | -12 | 0.044 |  |  | - | - | NA | Left | - | - |
| 3 | 4632 | -4 | 22 | 50 | 0.080 |  |  | Area 6mr / preSMA | 1 | NHA | Left | SFG | 71 |
| 4 | 2792 | -46 | -68 | 26 | 0.066 |  |  | Area PGp (IPL) | 45 | HA | Left | LOCs | 66 |
| 5 | 2288 | -22 | -12 | -16 | 0.050 |  |  | - | - | NA | Left | HC | 38 |
| 6 | 2144 | 36 | 24 | -6 | 0.054 |  |  | Area Id7 | 17 | NHA | Right | FOC | 65 |
| 6 | 2144 | 34 | 38 | -10 | 0.036 |  |  | Area Fo7 | 59 | HA | Right | FP | 61 |
|  |  |  |  |  |  |  |  |  |  |  |  |  |  |
| **Semantic (all), choice tasks > Non-semantic or less semantic baseline, choice tasks** | | | | | | | | | | | | | |
| *ALE-analysis, cluster forming threshold: p < .001; cluster extent correction: FWE p < .001* | | | | | | | | | | | | | |
| 1 | 23680 | -54 | -40 | 2 | 0.106 | - | - | - | - | NA | Left | MTGp | 26 |
| 1 | 23680 | -56 | -4 | -14 | 0.090 | - | - | Area TE 3 | 2 | NHA | Left | STGa | 41 |
| 1 | 23680 | -60 | -26 | -4 | 0.047 | - | - | - | - | NA | Left | MTGp | 34 |
| 2 | 18024 | -50 | 30 | 2 | 0.073 | - | - | Area OP9 | 54 | HA | Left | IFG PTr | 57 |
| 2 | 18024 | -52 | 24 | 14 | 0.067 | - | - | Area 45 | 38 | NHA | Left | IFG Ptr | 43 |
| 3 | 8600 | -34 | -34 | -18 | 0.063 | - | - | CA1 (Hippocampus) | 30 | NHA | Left | TFCp | 63 |
| 3 | 8600 | -40 | -46 | -20 | 0.062 | - | - | Area FG4 | 66 | HA | Left | TOFC | 33 |
| 4 | 7568 | -44 | -60 | 24 | 0.069 | - | - | Area PFm (IPL) | 11 | NHA | Left | AG | 50 |
| 5 | 3512 | 54 | -32 | 2 | 0.048 | - | - | - | - | NA | Right | STGp | 34 |
| 5 | 3512 | 58 | -24 | -2 | 0.038 | - | - | Area TE 3 | 1 | NHA | Right | STGp | 34 |
| 5 | 3512 | 50 | -20 | 4 | 0.032 | - | - | Area TE 1 | 11 | NHA | Right | HG | 23 |
| 6 | 2224 | 60 | -4 | -4 | 0.050 | - | - | Area TE 3 | 52 | HA | Right | STGa | 43 |
| 6 | 2224 | 56 | 0 | -16 | 0.046 | - | - | Area TE 3 | 28 | NHA | Right | STGa | 47 |
|  |  |  |  |  |  |  |  |  |  |  |  |  |  |
| **Sentences/Narratives, choice tasks > Non-semantic or less semantic baseline, choice tasks** | | | | | | | | | | | | | |
| *ALE-analysis, cluster forming threshold: p < .001; cluster extent correction: FWE p < .001* | | | | | | | | | | | | | |
| 1 | 11816 | -54 | -40 | 2 | 0.090 | - | - | - | - | NA | Left | MTGp | 26 |
| 1 | 11816 | -56 | -4 | -14 | 0.088 | - | - | Area TE 3 | 17 | NHA | Left | STGa | 41 |
| 1 | 11816 | -48 | 14 | -26 | 0.026 | - | - | - | - | NA | Left | TP | 59 |
| 2 | 3192 | -50 | 28 | 4 | 0.051 | - | - | Area OP9 | 74 | HA | Left | IFG PTr | 44 |
| 3 | 2256 | -44 | -60 | 24 | 0.048 | - | - | Area PFm (IPL) | 11 | NHA | Left | AG | 50 |
| 3 | 2256 | -50 | -56 | 16 | 0.035 | - | - | Area PGp (IPL) | 3 | NHA | Left | AG | 46 |
| 4 | 2056 | -40 | -46 | -20 | 0.046 | - | - | Area FG4 | 66 | HA | Left | TOFC | 33 |
| 4 | 2056 | -40 | -56 | -20 | 0.041 | - | - | Area FG4 | 62 | HA | Left | TOFC | 72 |
| 4 | 2056 | -32 | -36 | -16 | 0.025 | - | - | CA1 (Hippocampus) | 38 | NHA | Left | TFCp | 47 |
| 5 | 2048 | 50 | 14 | -22 | 0.035 | - | - | - | - | NA | Right | TP | 77 |
| 5 | 2048 | 56 | 0 | -18 | 0.035 | - | - | Area TE 3 | 15 | NHA | Right | STGa | 35 |
|  |  |  |  |  |  |  |  |  |  |  |  |  |  |
| **Single-Words/Word-Pairs, choice tasks > Non-semantic or less semantic baseline, choice tasks** | | | | | | | | | | | | | |
| *ALE-analysis, cluster forming threshold: p < .001; cluster extent correction: FWE p < .001* | | | | | | | | | | | | | |
| 1 | 5832 | -48 | 24 | 20 | 0.045 | - | - | Area 45 | 20 | NHA | Left | IFG PTr | 30 |
| 1 | 5832 | -48 | 34 | 2 | 0.041 | - | - | Area OP9 | 19 | NHA | Left | IFG PTr | 42 |
| 1 | 5832 | -32 | 34 | -12 | 0.026 | - | - | Area FO3 | 14 | NHA | Left | FOC | 46 |
| 2 | 2688 | -36 | -32 | -20 | 0.052 | - | - | CA1 (Hippocampus) | 26 | NHA | Left | TFCp | 60 |
| 2 | 2688 | -48 | -52 | -16 | 0.032 | - | - | Area FG4 | 74 | HA | Left | ITGt | 52 |
| 3 | 2400 | -60 | -38 | 2 | 0.036 | - | - | - | - | NA | Left | MTGp | 28 |
| 3 | 2400 | -60 | -10 | 4 | 0.036 | - | - | Area TE 3 | 25 | HA | Left | PT | 26 |
| 3 | 2400 | -60 | -24 | -4 | 0.025 | - | - | - | - | NA | Left | STGp | 31 |
| 4 | 1480 | -4 | 20 | 48 | 0.056 | - | - | Area 6mr / preSMA | 5 | NHA | Left | PG | 49 |
|  |  |  |  |  |  |  |  |  |  |  |  |  |  |
| **Sentences/Narratives, choice tasks > Single-Words/Word-Pairs, choice tasks** | | | | | | | | | | | | | |
| *Subtraction analysis, p < .001; minimum cluster volume: 200 mm3* | | | | | | | | | | | | | |
| 1 | 3200 | -56 | -3.2 | -13 | - | < .001 | 3.891 | Area TE 3 | 3 | NHA | Left | STGa | 51 |
| 2 | 424 | 55 | 10 | -20 | - | < .001 | 3.719 | Area TE 3 | 17 | NHA | Right | TP | 81 |
| 2 | 424 | 52 | 14 | -18 | - | < .001 | 3.432 | Area TE 3 | 15 | NHA | Right | TP | 87 |
|  |  |  |  |  |  |  |  |  |  |  |  |  |  |
| **Single-Words/Word-Pairs, choice tasks > Sentences/Narratives, choice tasks** | | | | | | | | | | | | | |
| *Subtraction analysis, p < .001; minimum cluster volume: 200 mm3* | | | | | | | | | | | | | |
| - | - | - | - | - | - | - | - | - | - | - | - | - | - |

**Supplementary Table 5. All activation clusters and local maxima for verbal semantic cognition.** Coordinates x, y and z reported in the MNI coordinate system; “Clust no”: Cluster number in the individual contrast; “ALE”: Activation Likelihood Estimate values output from Ginger ALE, along with P and Z values; “Cytoarchitecture”: cytoarchitectonic information for foci assigned by the JuBrain Anatomy Toolbox (SPM), based on the Maximum Probability Map; “% cyto”: probability of the coordinate falling into the specified Cytoarchitecture, as an output of the Anatomy Toolbox; “Assignment”: Type of assignment of coordinate into the specified Cytoarchitecture, as an output of the Anatomy Toolbox – HA: Hard Assignment, NHA: No Hard Assignment, NA: No Assignment; “Hem”: hemisphere; “Macroanatomy”: Assignment of the foci and to the Harvard-Oxford microanatomical atlas; “% macro”: probability of the coordinate falling into the assigned region by the Harvard-Oxford microanatomical atlas; "AG": Angular Gyrus; "AMYG": Amygdala; "CGa": Cingulate Gyrus, anterior; "CGp": Cingulate Gyrus, posterior; "COP": Central Opercular Cortex; "CRcr-I": Cerebellum Crus I; "CRcr-II": Cerebellum Crus II; "FMC": Frontal Medial Cortex; "FO": Frontal Operculum Cortex; "FOC": Frontal Orbital Cortex; "FP": Frontal Pole; "HC": Hippocampus; "HG": Heschl's Gyrus; "IC": Insular Cortex; "IFG POp": Inferior Frontal Gyrus, pars opercularis; "IFG PTr": Inferior Frontal Gyrus, pars triangularis; "IFGt": Inferior Frontal Gyrus, temporooccipital; "ITGp": Inferior Temporal Gyrus, posterior; "ITGt": Inferior Temporal Gyrus, temporooccipital; "JLC": Juxtapositional Lobule Cortex; "LOCi": Lateral Occipital Cortex, inferior; "LOCs": Lateral Occipital Cortex, superior; "MFG": Middle Frontal Gyrus; "MTGa": Middle Temporal Gyrus, anterior; "MTGp": Middle Temporal Gyrus, posterior; "MTGt": Middle Temporal Gyrus, temporooccipital; "OFC": Occipital Fusiform Gyrus; "OP": Occipital Pole; "PAC": Paracingulate Gyrus ; "PC": Precuneous Cortex; "PGp": Parahippocampal Gyrus, posterior; "POC": Parietal Operculum Cortex; "PP": Planum Polare; "PRG": Precentral Gyrus; "PT": Planum Temporale; "RC": Right Caudate; "SFG": Superior Frontal Gyrus; "SGp": Supramarginal Gyrus, posterior; "SPL": Superior Parietal Lobule; "STGa": Superior Temporal Gyrus, anterior; "STGp": Superior Temporal Gyrus, posterior; "STGs": Superior Temporal Gyrus, superior; "TFCp": Temporal Fusiform Cortex, posterior; "TOFC": Temporal Occipital Fusiform Cortex; "TP": Temporal Pole
