# Supplementary material for: The Role of the Right Language Network and the Multiple‐Demand Network in Verbal Semantics: Insights From an Activation Likelihood Estimation Meta‐Analysis of 561 Functional Neuroimaging Studies
Source: Hum Brain Mapp. 2025 Dec 20;46(18):e70415. doi: 10.1002/hbm.70415 (PMC12718395; doi:10.1002/hbm.70415)
Supplement: Supplementary file 7 — Table S6: All activation clusters and local maxima for Semantic Control (Separate ALE Meta‐Analyses, Conjunction and Subtraction Analyses). Coordinates (X, Y and Z) are reported in the MNI coordinate system; Clust no: cluster number in the individual contrast; ALE: activation likelihood estimation values output from GingerALE, along with p and Z values; Cytoarchitecture: cytoarchitectonic information for foci assigned by the JuBrain Anatomy Toolbox (SPM), based on the Maximum Probability Map; % cyto: probability of the coordinate falling into the specified Cytoarchitecture, as an output of the Anatomy Toolbox; Assignment: type of assignment of coordinate into the specified Cytoarchitecture, as an output of the Anatomy Toolbox—HA: hard assignment, NHA: no hard assignment, NA: no assignment, Hem: hemisphere; Macroanatomy: assignment of the foci and to the Harvard‐Oxford microanatomical atlas; % macro: probability of the coordinate falling into the assigned region by the Harvard‐Oxford microanatomical atlas. AG, angular gyrus; AMYG, amygdala; CGa, cingulate gyrus, anterior; CGp, cingulate gyrus, posterior; COP, central opercular cortex; CRcr‐I, cerebellum crus I; CRcr‐II, cerebellum crus II; FMC, frontal medial cortex; FO, frontal operculum cortex; FOC, frontal orbital cortex; FP, frontal pole; HC, hippocampus; HG, Heschl's gyrus; IC, insular cortex; IFG POp, inferior frontal gyrus, pars opercularis; IFG PTr, inferior frontal gyrus, pars triangularis; IFGt, inferior frontal gyrus, temporooccipital; ITGp, inferior temporal gyrus, posterior; ITGt, inferior temporal gyrus, temporooccipital; JLC, juxtapositional lobule cortex; LOCi, lateral occipital cortex, inferior; LOCs, lateral occipital cortex, superior; MFG, middle frontal gyrus; MTGa, middle temporal gyrus, anterior; MTGp, middle temporal gyrus, posterior; MTGt, middle temporal gyrus, temporooccipital; OFC, occipital fusiform gyrus; OP, occipital pole; PAC, paracingulate gyrus; PC, precuneous cortex; PGp, parahippoc [file HBM-46-e70415-s006.docx]

| **Cluster** | **Size (mm^3^**) | **X** | **Y** | **Z** | **ALE** | **P** | **Z** | **Cytoarchitecture** | **% cyto** | **Assignment** | **Hem.** | **Macroanatomy** | **% macro** |
| --- | --- | --- | --- | --- | --- | --- | --- | --- | --- | --- | --- | --- | --- |
| **Semantic control (all)** | | | | | | | | | | | | | |
| *ALE-analysis, cluster forming threshold: p < .001; cluster extent correction: FWE p < .001* | | | | | | | | | | | | | |
| 1 | 25904 | -48 | 22 | 20 | 0.113 | - | - | Area 45 | 9 | NHA | Left | IFG POp | 25 |
| 1 | 25904 | -48 | 26 | -2 | 0.075 | - | - | Area OP9 | 62 | HA | Left | FO | 33 |
| 1 | 25904 | -44 | 42 | -10 | 0.040 | - | - | Area Fo6 | 44 | HA | Left | FP | 67 |
| 1 | 25904 | -30 | 24 | -16 | 0.030 | - | - | Area Fo3 | 10 | NHA | Left | - | - |
| 2 | 7832 | -2 | 20 | 52 | 0.074 | - | - | Area 6mr / preSMA | 8 | NHA | Left | SFG | 58 |
| 2 | 7832 | -2 | 30 | 44 | 0.045 | - | - | - | - | NA | Left | SFG | 39 |
| 2 | 7832 | -8 | 30 | 24 | 0.028 | - | - | Area 33 | 23 | NHA | Left | CGa | 46 |
| 3 | 6048 | -52 | -40 | 4 | 0.057 | - | - | - | - | NA | Left | - | - |
| 3 | 6048 | -46 | -56 | -12 | 0.053 | - | - | Area FG4 | 72 | HA | Left | ITGt | 51 |
| 3 | 6048 | -44 | -46 | -18 | 0.048 | - | - | Area FG4 | 92 | HA | Left | ITGt | 23 |
| 3 | 6048 | -58 | -50 | 8 | 0.030 | - | - | - | - | NA | Left | MTGt | 41 |
| 3 | 6048 | -50 | -66 | -4 | 0.030 | - | - | - | - | NA | Left | LOCi | 60 |
| 4 | 2600 | 52 | 24 | 24 | 0.053 | - | - | Area 45 | 30 | NHA | Right | IFG POp | 22 |
| 5 | 2280 | 32 | 24 | -6 | 0.049 | - | - | - | - | NA | Right | FOC | 50 |
|  |  |  |  |  |  |  |  |  |  |  |  |  |  |
| **Semantic control, Sentences/Narratives** | | | | | | | | | | | | | |
| *ALE-analysis, cluster forming threshold: p < .001; cluster extent correction: FWE p < .001* | | | | | | | | | | | | | |
| 1 | 12936 | -48 | 24 | 20 | 0.061 | - | - | Area 45 | 14 | NHA | Left | IFG PTr | 30 |
| 1 | 12936 | -50 | 28 | 4 | 0.051 | - | - | Area OP9 | 71 | HA | Left | IFG PTr | 44 |
| 1 | 12936 | -44 | 24 | -2 | 0.048 | - | - | Area OP9 | 35 | HA | Left | FO | 52 |
| 1 | 12936 | -30 | 22 | -2 | 0.033 | - | - | Area Id7 | 28 | NHA | Left | IC | 56 |
| 1 | 12936 | -46 | 10 | 16 | 0.025 | - | - | Area 44 | 18 | NHA | Left | - | - |
| 2 | 2192 | -52 | -40 | 4 | 0.044 | - | - | - | - | NA | Left | - | - |
| 2 | 2192 | -60 | -50 | 8 | 0.029 | - | - | - | - | NA | Left | MTGt | 40 |
| 3 | 1640 | -44 | -44 | -20 | 0.037 | - | - | Area FG4 | 88 | HA | Left | TFCp | 31 |
| 3 | 1640 | -46 | -56 | -12 | 0.026 | - | - | Area FG4 | 72 | HA | Left | ITGt | 51 |
| 4 | 1392 | 42 | 20 | 22 | 0.035 | - | - | - | - | NA | Right | - | - |
| 4 | 1392 | 50 | 24 | 24 | 0.026 | - | - | Area 45 | 20 | NHA | Right | MFG | 28 |
|  |  |  |  |  |  |  |  |  |  |  |  |  |  |
| **Semantic control, Single-Words/Word-Pairs** | | | | | | | | | | | | | |
| *ALE-analysis, cluster forming threshold: p < .001; cluster extent correction: FWE p < .001* | | | | | | | | | | | | | |
| 1 | 13288 | -48 | 18 | 22 | 0.064 | - | - | Area 44 | 19 | NHA | Left | IFG POp | 51 |
| 1 | 13288 | -50 | 26 | -4 | 0.037 | - | - | Area OP9 | 54 | HA | Left | IFG PTr | 30 |
| 1 | 13288 | -52 | 38 | 4 | 0.025 | - | - | Area 45 | 23 | NHA | Left | FP | 50 |
| 1 | 13288 | -50 | 8 | 40 | 0.020 | - | - | Area 44 | 7 | NHA | Left | MFG | 42 |
| 1 | 13288 | -46 | 6 | 46 | 0.020 | - | - | Area 44 | 0 | NHA | Left | MFG | 50 |
| 2 | 5632 | -2 | 30 | 46 | 0.037 | - | - | - | - | NA | Left | SFG | 56 |
| 2 | 5632 | -2 | 18 | 52 | 0.036 | - | - | Area 6mr / preSMA | 10 | NHA | Left | SFG | 46 |
| 2 | 5632 | -4 | 16 | 56 | 0.035 | - | - | Area 6mr / preSMA | 56 | HA | Left | SFG | 68 |
| 2 | 5632 | -6 | 22 | 46 | 0.033 | - | - | - | - | NA | Left | PAC | 47 |
| 2 | 5632 | -2 | 28 | 34 | 0.028 | - | - | - | - | NA | Left | PAC | 70 |
| 3 | 1464 | -46 | -54 | -12 | 0.029 | - | - | Area FG4 | 81 | HA | Left | ITGt | 50 |
| 3 | 1464 | -54 | -50 | -4 | 0.025 | - | - | - | - | NA | Left | - | - |
| 3 | 1464 | -52 | -66 | -4 | 0.022 | - | - | Area hOc4la | 0 | NHA | Left | LOCi | 59 |
|  |  |  |  |  |  |  |  |  |  |  |  |  |  |
| **Semantic control, Single-Words/Word-Pairs > Sentences/Narratives** | | | | | | | | | | | | | |
| *Subtraction analysis, p < .01, minimum cluster volume: 200 mm^3^* | | | | | | | | | | | | | |
| 1 | 936 | -8 | 26 | 34 | - | <.001 | 3.353 | - | - | NA | Left | PAC | 60 |
| 1 | 936 | -7 | 29 | 37 | - | 0.003 | 2.748 | - | - | NA | Left | PAC | 56 |
| 2 | 472 | -40 | 12 | 26 | - | 0.004 | 2.678 | Area 44 | 10 | NHA | Left | IFG POp | 29 |
| 2 | 472 | -38 | 4 | 22 | - | 0.005 | 2.597 | Area 44 | 2 | NHA | Left | PRG | 9 |
| 2 | 472 | -44 | 6 | 22 | - | 0.006 | 2.543 | Area 44 | 46 | HA | Left | PRG | 28 |
|  |  |  |  |  |  |  |  |  |  |  |  |  |  |
| **Semantic control, Sentences/Narratives > Single-Words/Word-Pairs** | | | | | | | | | | | | | |
| *Subtraction analysis, p < .01, minimum cluster volume: 200 mm^3^* | | | | | | | | | | | | | |
| No clusters found | | | | | | | | | | | | | |

**Supplementary Table 6. All activation clusters and local maxima for Semantic Control (Separate ALE Meta-Analyses, Conjunction and Subtraction Analyses).** Coordinates x, y and z reported in the MNI coordinate system; “Clust no”: Cluster number in the individual contrast; “ALE”: Activation Likelihood Estimation values output from GingerALE, along with P and Z values; “Cytoarchitecture”: cytoarchitectonic information for foci assigned by the JuBrain Anatomy Toolbox (SPM), based on the Maximum Probability Map; “% cyto”: probability of the coordinate falling into the specified Cytoarchitecture, as an output of the Anatomy Toolbox; “Assignment”: Type of assignment of coordinate into the specified Cytoarchitecture, as an output of the Anatomy Toolbox – HA: Hard Assignment, NHA: No Hard Assignment, NA: No Assignment “Hem”: Hemisphere; “Macroanatomy”: Assignment of the foci and to the Harvard-Oxford microanatomical atlas; “% macro”: probability of the coordinate falling into the assigned region by the Harvard-Oxford microanatomical atlas; "AG": Angular Gyrus; "AMYG": Amygdala; "CGa": Cingulate Gyrus, anterior; “CGp": Cingulate Gyrus, posterior; "COP": Central Opercular Cortex; "CRcr-I": Cerebellum Crus I; "CRcr-II": Cerebellum Crus II; "FMC": Frontal Medial Cortex; "FO": Frontal Operculum Cortex; "FOC": Frontal Orbital Cortex; "FP": Frontal Pole; "HC": Hippocampus; "HG": Heschl's Gyrus; "IC": Insular Cortex; "IFG POp": Inferior Frontal Gyrus, pars opercularis; "IFG PTr": Inferior Frontal Gyrus, pars triangularis; "IFGt": Inferior Frontal Gyrus, temporooccipital; "ITGp": Inferior Temporal Gyrus, posterior; "ITGt": Inferior Temporal Gyrus, temporooccipital; "JLC": Juxtapositional Lobule Cortex; "LOCi": Lateral Occipital Cortex, inferior; "LOCs": Lateral Occipital Cortex, superior; "MFG": Middle Frontal Gyrus; "MTGa": Middle Temporal Gyrus, anterior; "MTGp": Middle Temporal Gyrus, posterior; "MTGt": Middle Temporal Gyrus, temporooccipital; "OFC": Occipital Fusiform Gyrus; "OP": Occipital Pole; "PAC": Paracingulate Gyrus ; "PC": Precuneous Cortex; "PGp": Parahippocampal Gyrus, posterior; "POC": Parietal Operculum Cortex; "PP": Planum Polare; "PRG": Precentral Gyrus; "PT": Planum Temporale; "RC": Right Caudate; "SFG": Superior Frontal Gyrus; "SGp": Supramarginal Gyrus, posterior; "SPL": Superior Parietal Lobule; "STGa": Superior Temporal Gyrus, anterior; "STGp": Superior Temporal Gyrus, posterior; "STGs": Superior Temporal Gyrus, superior; "TFCp": Temporal Fusiform Cortex, posterior; "TOFC": Temporal Occipital Fusiform Cortex; "TP": Temporal Pole.
