# Supplementary material for: The Role of the Right Language Network and the Multiple‐Demand Network in Verbal Semantics: Insights From an Activation Likelihood Estimation Meta‐Analysis of 561 Functional Neuroimaging Studies
Source: Hum Brain Mapp. 2025 Dec 20;46(18):e70415. doi: 10.1002/hbm.70415 (PMC12718395; doi:10.1002/hbm.70415)
Supplement: Supplementary file 8 — Table S7: All activation clusters and local maxima for Social Cognition and Verbal Semantic Cognition (Separate ALE Meta‐Analyses, Conjunction and Subtraction Analyses). Coordinates ( X, Y and Z ) are reported in the MNI coordinate system; Clust no: cluster number in the individual contrast; ALE: activation likelihood estimation values output from GingerALE, along with p and Z values; Cytoarchitecture: cytoarchitectonic information for foci assigned by the JuBrain Anatomy Toolbox (SPM), based on the maximum probability map; % cyto: probability of the coordinate falling into the specified Cytoarchitecture, as an output of the Anatomy Toolbox; Assignment: type of assignment of coordinate into the specified Cytoarchitecture, as an output of the Anatomy Toolbox—HA: hard assignment, NHA: no hard assignment, NA: no assignment, Hem: hemisphere; Macroanatomy: Assignment of the foci and to the Harvard‐Oxford microanatomical atlas; % macro: probability of the coordinate falling into the assigned region by the Harvard‐Oxford microanatomical atlas. aCG: cingulate gyrus, anterior; AG, angular gyrus; AMYG, amygdala; COP, central opercular cortex; CRcr‐I, cerebellum crus I; CRcr‐II, cerebellum crus II; FMC, frontal medial cortex; FO, frontal operculum cortex; FOC, frontal orbital cortex; FP, frontal pole; HC, hippocampus; HG, Heschl's gyrus; IC, insular cortex; IFG POp, inferior frontal gyrus, pars opercularis; IFG PTr, inferior frontal gyrus, pars triangularis; IFGt, inferior frontal gyrus, temporooccipital; ITGp, inferior temporal gyrus, posterior; ITGt, inferior temporal gyrus, temporooccipital; JLC, juxtapositional lobule cortex; LOCi, lateral occipital cortex, inferior; LOCs, lateral occipital cortex, superior; MFG, middle frontal gyrus; MTGa, middle temporal gyrus, anterior; MTGp, middle temporal gyrus, posterior; MTGt, middle temporal gyrus, temporooccipital; OFC, occipital fusiform gyrus; OP, occipital pole; PAC, paracingulate gyrus; PC, precuneous cortex; pCG, cingulate g [file HBM-46-e70415-s002.docx]

| **Clust no** | **Size (mm^3^**) | **X** | **Y** | **Z** | **ALE** | **P** | **Z** | **Cytoarchitecture** | **% cyto** | **Assignment** | **Hem.** | **Macroanatomy** | **% macro** |
| --- | --- | --- | --- | --- | --- | --- | --- | --- | --- | --- | --- | --- | --- |
| **Nonverbal Theory of Mind > Nonverbal baseline** | | | | | | | | | | | | | |
| *ALE-analysis, cluster forming threshold: p < .001; cluster extent correction: FWE p < .001* | | | | | | | | | | | | | |
| 1 | 10864 | -54 | -52 | 16 | 0.132 | - | - | Area PGa (IPL) | 0 | NHA | Left | AG | 38 |
| 1 | 10864 | -54 | -38 | 0 | 0.086 | - | - | Area TE 4 | 1 | NHA | Left | MTGp | 40 |
| 1 | 10864 | -50 | -72 | 16 | 0.057 | - | - | Area hOc4la | 17 | NHA | Left | LOCs | 60 |
| 2 | 10576 | 54 | -50 | 16 | 0.115 | - | - | Area PGa (IPL) | 32 | NHA | Right | AG | 50 |
| 2 | 10576 | 52 | -60 | 14 | 0.093 | - | - | Area PGp (IPL) | 15 | NHA | Right | LOCs | 24 |
| 2 | 10576 | 50 | -38 | 4 | 0.083 | - | - | Area TE 4 | 6 | NHA | Right | MTGt part | 25 |
| 2 | 10576 | 50 | -70 | 4 | 0.049 | - | - | Area hOc5 [V5/MT] | 51 | HA | Right | LOCi | 69 |
| 3 | 7280 | -4 | -52 | 32 | 0.108 | - | - | - | - | NA | Left | CGp | 72 |
| 3 | 7280 | 0 | -54 | 46 | 0.083 | - | - | - | - | NA | Right | PC | 61 |
| 4 | 7232 | -46 | 26 | -6 | 0.128 | - | - | Area OP9 | 31 | NHA | Left | FOC | 50 |
| 4 | 7232 | -52 | 24 | 6 | 0.088 | - | - | Area OP9 | 38 | HA | Left | IFG PTr | 52 |
| 4 | 7232 | -50 | 18 | 18 | 0.084 | - | - | Area 44 | 26 | NHA | Left | IFG POp | 52 |
| 4 | 7232 | -42 | 8 | 26 | 0.052 | - | - | Area 44 | 35 | NHA | Left | IFG POp | 30 |
| 4 | 7232 | -34 | 26 | 2 | 0.047 | - | - | Area Id7 | 91 | HA | Left | FOC | 26 |
| 5 | 6208 | -8 | 56 | 34 | 0.132 | - | - | - | - | NA | Left | FP | 58 |
| 5 | 6208 | 6 | 58 | 22 | 0.072 | - | - | Area p32 | 56 | HA | Right | FP | 46 |
| 6 | 5464 | 60 | -8 | -18 | 0.090 | - | - | Area TE 5 | 54 | HA | Right | MTGp | 30 |
| 6 | 5464 | 46 | 14 | -32 | 0.078 | - | - | - | - | NA | Right | TP | 72 |
| 6 | 5464 | 50 | 10 | -26 | 0.066 | - | - | Area TE 5 | 30 | NHA | Right | TP | 78 |
| 7 | 4320 | -58 | -10 | -14 | 0.096 | - | - | Area TE 5 | 92 | HA | Left | MTGa | 31 |
| 7 | 4320 | -52 | 10 | -24 | 0.067 | - | - | Area TE 5 | 10 | NHA | Left | TP | 84 |
| 8 | 3560 | 56 | 30 | 4 | 0.110 | - | - | Area 45 | 77 | HA | Right | IFG PTr | 62 |
| 8 | 3560 | 48 | 20 | 24 | 0.056 | - | - | Area 45 | 14 | NHA | Right | IFG POp | 38 |
| 9 | 2856 | -4 | 14 | 58 | 0.099 | - | - | Area 6mr / preSMA | 79 | HA | Left | SFG | 61 |
| 10 | 1856 | -42 | -48 | -18 | 0.077 | - | - | Area FG4 | 81 | HA | Left | TOFC | 31 |
| 11 | 1848 | 0 | 52 | -14 | 0.069 | - | - | - | - | NA | Right | FMC | 63 |
|  |  |  |  |  |  |  |  |  |  |  |  |  |  |
| **Social Cues > Non-social baseline** | | | | | | | | | | | | | |
| *ALE-analysis, cluster forming threshold: p < .001; cluster extent correction: FWE p < .001* | | | | | | | | | | | | | |
| 1 | 20576 | 52 | -62 | 4 | 0.104 | - | - | Area hOc5 [V5/MT] | 43 | NHA | Right | LOCi | 49 |
| 1 | 20576 | 64 | -24 | 4 | 0.044 | - | - | Area TE 3 | 18 | NHA | Right | - | - |
| 1 | 20576 | 54 | -12 | 0 | 0.042 | - | - | Area TE 1.0 | 4 | NHA | Right | - | - |
| 1 | 20576 | 52 | -20 | -4 | 0.040 | - | - | Area TE 4 | 63 | HA | Right | STGp | 31 |
| 1 | 20576 | 54 | -4 | -6 | 0.036 | - | - | Area TE 4 | 37 | HA | Right | - | - |
| 2 | 13680 | -50 | -68 | 8 | 0.077 | - | - | Area hOc5 [V5/MT] | 5 | NHA | Left | LOCi | 65 |
| 2 | 13680 | -50 | -18 | 6 | 0.055 | - | - | Area TE 1.0 | 57 | HA | Left | - | - |
| 2 | 13680 | -44 | -68 | -10 | 0.055 | - | - | Area FG2 | 49 | HA | Left | LOCi | 42 |
| 2 | 13680 | -48 | -40 | 22 | 0.044 | - | - | Area PFcm (IPL) | 56 | HA | Left | POC | 35 |
| 2 | 13680 | -50 | -60 | 26 | 0.044 | - | - | Area PFm (IPL) | 33 | HA | Left | AG | 50 |
| 2 | 13680 | -58 | -34 | 12 | 0.043 | - | - | Area PFcm (IPL) | 42 | NHA | Left | PT | 41 |
| 2 | 13680 | -54 | -22 | 18 | 0.037 | - | - | Area OP1 [SII] | 70 | HA | Left | COP | 51 |
| 2 | 13680 | -60 | -32 | 26 | 0.034 | - | - | Area PFcm (IPL) | 37 | HA | Left | SGa | 41 |
| 3 | 2472 | 44 | -46 | -18 | 0.102 | - | - | Area FG4 | 79 | HA | Right | TOFC | 54 |
| 4 | 2344 | 0 | 12 | 50 | 0.063 | - | - | - | - | NA | Right | PAC | 41 |
| 4 | 2344 | -4 | 0 | 62 | 0.042 | - | - | Area 6mr / preSMA | 42 | HA | Left | JLC | 87 |
| 5 | 2048 | -54 | 14 | 12 | 0.054 | - | - | Area 44 | 51 | HA | Left | IFG POp | 80 |
| 5 | 2048 | -48 | 18 | -8 | 0.038 | - | - | - | - | NA | Left | - | - |
|  |  |  |  |  |  |  |  |  |  |  |  |  |  |
| **Nonverbal Theory of Mind & Sentences/Narratives** | | | | | | | | | | | | | |
| *Conjunction analysis, p < .001; minimum cluster volume: 200 mm3* | | | | | | | | | | | | | |
| 1 | 7392 | -54 | -38 | 0 | 0.086 | - | - | Area TE 4 | 1 | NHA | Left | MTGp | 40 |
| 1 | 7392 | -46 | -58 | 24 | 0.086 | - | - | Area PGp (IPL) | 2 | NHA | Left | AG | 61 |
| 1 | 7392 | -58 | -44 | 6 | 0.076 | - | - | - | - | NA | Left | MTGt | 23 |
| 1 | 7392 | -52 | -48 | 8 | 0.074 | - | - | - | - | NA | Left | MTGt | 34 |
| 2 | 6144 | -48 | 28 | -2 | 0.089 | - | - | Area OP9 | 62 | HA | Left | IFG PTr | 28 |
| 2 | 6144 | -52 | 24 | 8 | 0.087 | - | - | Area OP9 | 48 | HA | Left | IFG PTr | 44 |
| 2 | 6144 | -50 | 20 | 16 | 0.081 | - | - | Area 44 | 20 | NHA | Left | IFG POp | 37 |
| 2 | 6144 | -42 | 12 | 26 | 0.047 | - | - | Area 44 | 19 | NHA | Left | IFG POp | 32 |
| 3 | 3648 | -58 | -10 | -14 | 0.096 | - | - | Area TE 5 | 92 | HA | Left | MTGa | 31 |
| 3 | 3648 | -52 | 10 | -24 | 0.067 | - | - | Area TE 5 | 10 | NHA | Left | TP | 84 |
| 4 | 2760 | 58 | -6 | -14 | 0.066 | - | - | Area TE 5 | 57 | HA | Right | MTGa | 32 |
| 4 | 2760 | 56 | -2 | -20 | 0.065 | - | - | Area TE 5 | 86 | HA | Right | MTGa | 39 |
| 4 | 2760 | 50 | 12 | -26 | 0.063 | - | - | Area TE 5 | 10 | NHA | Right | TP | 84 |
| 4 | 2760 | 50 | -16 | -10 | 0.046 | - | - | Area TE 4 | 56 | HA | Right | MTGp | 29 |
| 5 | 1488 | -4 | 12 | 52 | 0.054 | - | - | Area 6mr / preSMA | 61 | HA | Left | PAC | 49 |
| 5 | 1488 | -6 | 10 | 56 | 0.054 | - | - | Area 6mr / preSMA | 87 | HA | Left | SFG | 38 |
| 5 | 1488 | -8 | 14 | 62 | 0.048 | - | - | Area 6mr / preSMA | 68 | HA | Left | - | - |
| 6 | 1264 | -42 | -48 | -18 | 0.077 | - | - | Area FG4 | 81 | HA | Left | TOFC | 31 |
| 7 | 976 | 50 | -34 | 0 | 0.067 | - | - | Area TE 4 | 22 | NHA | Right | MTGp | 29 |
|  |  |  |  |  |  |  |  |  |  |  |  |  |  |
| **Social Cues & Sentences/Narratives** | | | | | | | | | | | | | |
| *Conjunction analysis, p < .001; minimum cluster volume: 200 mm3* | | | | | | | | | | | | | |
| 1 | 3120 | -52 | -48 | 8 | 0.071 | - | - | - | - | NA | Left | MTGt | 34 |
| 1 | 3120 | -50 | -60 | 26 | 0.044 | - | - | Area PFm (IPL) | 33 | HA | Left | AG | 50 |
| 1 | 3120 | -60 | -46 | 0 | 0.038 | - | - | - | - | NA | Left | MTGt | 42 |
| 1 | 3120 | -46 | -58 | 14 | 0.037 | - | - | - | - | NA | Left | AG | 28 |
| 2 | 1488 | 52 | -36 | 0 | 0.063 | - | - | Area TE 5 | 25 | NHA | Right | MTGp | 35 |
| 2 | 1488 | 52 | -20 | -4 | 0.038 | - | - | Area TE 4 | 63 | HA | Right | STGp | 31 |
| 2 | 1488 | 62 | -26 | 0 | 0.037 | - | - | Area TE 4 | 34 | NHA | Right | STGp | 29 |
| 3 | 1248 | 4 | 14 | 48 | 0.045 | - | - | Area 6mr / preSMA | 28 | NHA | Right | PAC | 73 |
| 3 | 1248 | -4 | 4 | 60 | 0.036 | - | - | Area 6mr / preSMA | 72 | HA | Left | JLC | 78 |
| 4 | 936 | -54 | 16 | 12 | 0.051 | - | - | Area 44 | 69 | HA | Left | IFG POp | 77 |
| 4 | 936 | -54 | 22 | 2 | 0.040 | - | - | Area 44 | 60 | HA | Left | IFG PTr | 57 |
| 5 | 72 | 54 | -4 | -8 | 0.036 | - | - | Area TE 4 | 61 | HA | Right | - | - |
| 6 | 40 | -62 | -24 | 4 | 0.033 | - | - | Area TE 4 | 33 | NHA | Left | - | - |
|  |  |  |  |  |  |  |  |  |  |  |  |  |  |
| **Nonverbal Theory of Mind > Sentences/Narratives** | | | | | | | | | | | | | |
| *Subtraction analysis, p < .001, minimum cluster volume: 200 mm^3^* | | | | | | | | | | | | | |
| 1 | 3888 | 53 | -51 | 16 | - | 0.000 | 3.891 | Area PGa (IPL) | 23 | NHA | Right | AG | 45 |
| 1 | 3888 | 54 | -67 | 17 | - | 0.000 | 3.719 | Area PGp (IPL) | 39 | HA | Right | LOCs | 59 |
| 2 | 3264 | 3 | -53 | 39 | - | 0.000 | 3.891 | - | - | NA | Right | PC | 78 |
| 3 | 1048 | 55 | 28 | 5 | - | 0.000 | 3.891 | Area 45 | 79 | HA | Right | IFG PTr | 75 |
| 4 | 904 | 5 | 60 | 18 | - | 0.000 | 3.891 | Area p32 | 49 | HA | Right | FP | 72 |
| 5 | 528 | -51 | -72 | 15 | - | 0.000 | 3.891 | Area hO c4la | 19 | NHA | Left | LOCs | 53 |
| 5 | 528 | -56 | -66 | 16 | - | 0.000 | 3.540 | Area PGp (IPL) | 30 | NHA | Left | LOCs | 53 |
| 6 | 464 | -56 | -45 | 18 | - | 0.000 | 3.891 | Area PGa (IPL) | 2 | NHA | Left | SGp | 35 |
| 6 | 464 | -52 | -54 | 20 | - | 0.000 | 3.195 | Area PGa (IPL) | 9 | NHA | Left | AG | 54 |
|  |  |  |  |  |  |  |  |  |  |  |  |  |  |
| **Sentences/Narratives > Nonverbal Theory of Mind** | | | | | | | | | | | | | |
| *Conjunction analysis, p < .001; minimum cluster volume: 200 mm3* | | | | | | | | | | | | | |
| 1 | 760 | -56 | -8 | -2 | - | 0.000 | 3.891 | Area TE 4 | 57 | HA | Left | STGa | 31 |
| 2 | 496 | -56 | -1 | -14 | - | 0.000 | 3.719 | Area TE 5 | 64 | HA | Left | STGa | 56 |
| 2 | 496 | -60 | 0 | -16 | - | 0.000 | 3.540 | Area TE 5 | 72 | HA | Left | MTGa | 53 |
| 2 | 496 | -50 | 0 | -12 | - | 0.000 | 3.432 | Area TE 5 | 16 | NHA | Left | PP | 33 |
| 3 | 320 | -52 | 30 | 16 | - | 0.000 | 3.719 | Area 45 | 63 | HA | Left | IFG PTr | 60 |
| 3 | 320 | -56 | 26 | 16 | - | 0.000 | 3.540 | Area 45 | 75 | HA | Left | IFG PTr | 56 |
| 3 | 320 | -60 | 22 | 16 | - | 0.000 | 3.239 | Area 45 | 64 | HA | Left | IFG PTr | 10 |
| 4 | 240 | 61 | -6 | -1 | - | 0.000 | 3.891 | Area TE 3 | 24 | NHA | Right | STGa | 27 |
| 4 | 240 | 61 | -11 | -2 | - | 0.000 | 3.540 | Area TE 4 | 31 | HA | Right | STGa | 11 |
|  |  |  |  |  |  |  |  |  |  |  |  |  |  |
| **Nonverbal Theory of Mind & Single-Words/Word-Pairs** | | | | | | | | | | | | | |
| *Conjunction analysis, p < .001; minimum cluster volume: 200 mm3* | | | | | | | | | | | | | |
| 1 | 5512 | -46 | 30 | -10 | 0.083 | 0.000 | 0.000 | Area OP9 | 12 | NHA | Left | FOC | 43 |
| 1 | 5512 | -50 | 20 | 18 | 0.082 | 0.000 | 0.000 | Area 44 | 19 | NHA | Left | IFG POp | 39 |
| 1 | 5512 | -42 | 6 | 26 | 0.047 | 0.000 | 0.000 | Area 44 | 32 | NHA | Left | PRG | 29 |
| 2 | 2568 | -54 | -38 | 0 | 0.086 | 0.000 | 0.000 | Area TE 4 | 1 | NHA | Left | MTGp | 40 |
| 3 | 1504 | -50 | -62 | 22 | 0.065 | 0.000 | 0.000 | Area PGp (IPL) | 13 | NHA | Left | LOCs | 33 |
| 4 | 1344 | -40 | -46 | -20 | 0.065 | 0.000 | 0.000 | Area FG4 | 72 | HA | Left | TOFC | 33 |
| 4 | 1344 | -44 | -60 | -10 | 0.046 | 0.000 | 0.000 | Area FG4 | 63 | HA | Left | ITGt | 40 |
| 5 | 1048 | -4 | 16 | 50 | 0.074 | 0.000 | 0.000 | Area 6mr / preSMA | 17 | NHA | Left | PAC | 53 |
| 6 | 80 | -34 | 24 | -2 | 0.038 | 0.000 | 0.000 | Area Id7 | 74 | HA | Left | IC | 38 |
| 7 | 8 | -42 | 22 | 2 | 0.034 | 0.000 | 0.000 | Area OP8 | 45 | HA | Left | FO | 68 |
|  |  |  |  |  |  |  |  |  |  |  |  |  |  |
| **Social Cues & Single-Words/Word-Pairs** | | | | | | | | | | | | | |
| *Conjunction analysis, p < .001; minimum cluster volume: 200 mm3* | | | | | | | | | | | | | |
| 1 | 792 | -2 | 14 | 50 | 0.057 | - | - | - | - | NA | Left | PAC | 51 |
| 2 | 576 | -54 | -44 | 6 | 0.049 | - | - | - | - | NA | Left | MTGt | 19 |
| 2 | 576 | -60 | -46 | 0 | 0.038 | - | - | - | - | NA | Left | MTGt | 42 |
| 3 | 504 | -50 | -60 | 26 | 0.044 | - | - | Area PFm (IPL) | 33 | HA | Left | AG | 50 |
| 4 | 432 | -52 | 16 | 12 | 0.039 | - | - | Area 44 | 64 | HA | Left | IFG POp | 64 |
| 4 | 432 | -54 | 24 | 0 | 0.037 | - | - | Area 45 | 40 | HA | Left | IFG PTr | 58 |
| 5 | 152 | -44 | -64 | -10 | 0.041 | - | - | Area FG2 | 40 | HA | Left | ITGt | 31 |
| 6 | 64 | -60 | -24 | 4 | 0.034 | - | - | Area TE 4 | 32 | NHA | Left | - | - |
| 7 | 56 | -52 | -14 | 2 | 0.036 | - | - | Area TE 1.2 | 14 | NHA | Left | - | - |
| 8 | 24 | -50 | -68 | 18 | 0.033 | - | - | Area PGp (IPL) | 27 | NHA | Left | LOCs | 64 |
| 9 | 8 | -48 | 16 | 8 | 0.032 | - | - | Area OP8 | 35 | HA | Left | IFG POp | 52 |
| 10 | 8 | -52 | -64 | 16 | 0.032 | - | - | Area PGp (IPL) | 13 | NHA | Left | LOCs | 38 |
|  |  |  |  |  |  |  |  |  |  |  |  |  |  |
| **Nonverbal Theory of Mind & Social Cues** | | | | | | | | | | | | | |
| *Conjunction analysis, p < .001; minimum cluster volume: 200 mm3* | | | | | | | | | | | | | |
| 1 | 7400 | 50 | -38 | 4 | 0.083 | - | - | Area TE 4 | 6 | NHA | Right | MTGt | 25 |
| 1 | 7400 | 54 | -46 | 12 | 0.081 | - | - | Area PFm (IPL) | 0 | NHA | Right | MTGt | 35 |
| 1 | 7400 | 52 | -60 | 10 | 0.070 | - | - | Area PGp (IPL) | 1 | NHA | Right | MTGt | 38 |
| 1 | 7400 | 50 | -70 | 4 | 0.049 | - | - | Area hOc5 [V5/MT] | 51 | HA | Right | LOCi | 69 |
| 2 | 4176 | -52 | -48 | 8 | 0.071 | - | - | - | - | NA | Left | MTGt | 34 |
| 2 | 4176 | -50 | -70 | 14 | 0.051 | - | - | Area hOc4la | 11 | NHA | Left | LOCi | 47 |
| 2 | 4176 | -50 | -60 | 26 | 0.044 | - | - | Area PFm (IPL) | 33 | HA | Left | AG | 50 |
| 2 | 4176 | -50 | -64 | 14 | 0.042 | - | - | Area PGp (IPL) | 5 | NHA | Left | LOCi | 25 |
| 2 | 4176 | -46 | -60 | 14 | 0.039 | - | - | - | - | NA | Left | MTGt | 21 |
| 3 | 608 | -54 | 20 | 6 | 0.043 | - | - | Area 44 | 89 | HA | Left | IFG POp | 41 |
| 3 | 608 | -54 | 18 | 12 | 0.043 | - | - | Area 44 | 60 | HA | Left | IFG POp | 66 |
| 3 | 608 | -48 | 20 | -8 | 0.037 | - | - | Area 45 | 11 | NHA | Left | FOC | 37 |
| 4 | 536 | -2 | 14 | 50 | 0.057 | - | - | - | - | NA | Left | PAC | 51 |
|  |  |  |  |  |  |  |  |  |  |  |  |  |  |

**Supplementary Table 7. All activation clusters and local maxima for Social processing and Verbal Semantic Cognition (Separate ALE Meta-Analyses, Conjunction and Subtraction Analyses).** Coordinates x, y and z reported in the MNI coordinate system; “Clust no”: Cluster number in the individual contrast; “ALE”: Activation Likelihood Estimation values output from GingerALE, along with P and Z values; “Cytoarchitecture”: cytoarchitectonic information for foci assigned by the JuBrain Anatomy Toolbox (SPM), based on the Maximum Probability Map; “% cyto”: probability of the coordinate falling into the specified Cytoarchitecture, as an output of the Anatomy Toolbox; “Assignment”: Type of assignment of coordinate into the specified Cytoarchitecture, as an output of the Anatomy Toolbox – HA: Hard Assignment, NHA: No Hard Assignment, NA: No Assignment “Hem”: hemisphere; “Macroanatomy”: Assignment of the foci and to the Harvard-Oxford microanatomical atlas; “% macro”: probability of the coordinate falling into the assigned region by the Harvard-Oxford microanatomical atlas; "aCG": Cingulate Gyrus, anterior; "AG": Angular Gyrus; "AMYG": Amygdala; "COP": Central Opercular Cortex; "CRcr-I": Cerebellum Crus I; "CRcr-II": Cerebellum Crus II; "FMC": Frontal Medial Cortex; "FO": Frontal Operculum Cortex; "FOC": Frontal Orbital Cortex; "FP": Frontal Pole; "HC": Hippocampus; "HG": Heschl's Gyrus; "IC": Insular Cortex; "IFG POp": Inferior Frontal Gyrus, pars opercularis; "IFG PTr": Inferior Frontal Gyrus, pars triangularis; "IFGt": Inferior Frontal Gyrus, temporooccipital; "ITGp": Inferior Temporal Gyrus, posterior; "ITGt": Inferior Temporal Gyrus, temporooccipital; "JLC": Juxtapositional Lobule Cortex; "LOCi": Lateral Occipital Cortex, inferior; "LOCs": Lateral Occipital Cortex, superior; "MFG": Middle Frontal Gyrus; "MTGa": Middle Temporal Gyrus, anterior; "MTGp": Middle Temporal Gyrus, posterior; "MTGt": Middle Temporal Gyrus, temporooccipital; "OFC": Occipital Fusiform Gyrus; "OP": Occipital Pole; "PAC": Paracingulate Gyrus ; "PC": Precuneous Cortex; "pCG": Cingulate Gyrus, posterior; "PGp": Parahippocampal Gyrus, posterior; "POC": Parietal Operculum Cortex; "PP": Planum Polare; "PRG": Precentral Gyrus; "PT": Planum Temporale; "RC": Right Caudate; "SFG": Superior Frontal Gyrus; "SGp": Supramarginal Gyrus, posterior; "SPL": Superior Parietal Lobule; "STGa": Superior Temporal Gyrus, anterior; "STGp": Superior Temporal Gyrus, posterior; "STGs": Superior Temporal Gyrus, superior; "TFCp": Temporal Fusiform Cortex, posterior; "TOFC": Temporal Occipital Fusiform Cortex; "TP": Temporal Pole
