# Supplementary material for: The Role of the Right Language Network and the Multiple‐Demand Network in Verbal Semantics: Insights From an Activation Likelihood Estimation Meta‐Analysis of 561 Functional Neuroimaging Studies
Source: Hum Brain Mapp. 2025 Dec 20;46(18):e70415. doi: 10.1002/hbm.70415 (PMC12718395; doi:10.1002/hbm.70415)
Supplement: Supplementary file 9 — Table S8: All activation clusters and local maxima for Verbal Semantic Cognition, split by external task demands (Separate ALE Meta‐Analyses). Coordinates ( X, Y and Z) are reported in the MNI coordinate system; Clust no: cluster number in the individual contrast; ALE: activation likelihood estimation values output from Ginger ALE, along with p and Z values; Cytoarchitecture: cytoarchitectonic information for foci assigned by the JuBrain Anatomy Toolbox (SPM), based on the maximum probability map; % cyto: probability of the coordinate falling into the specified Cytoarchitecture, as an output of the Anatomy Toolbox; Assignment: type of assignment of coordinate into the specified Cytoarchitecture, as an output of the Anatomy Toolbox—HA: hard assignment, NHA: no hard assignment, NA: no assignment, Hem: hemisphere; Macroanatomy: assignment of the foci and to the Harvard‐Oxford microanatomical atlas; % macro: probability of the coordinate falling into the assigned region by the Harvard‐Oxford microanatomical atlas. aCG, cingulate gyrus, anterior; AG, angular gyrus; AMYG, amygdala; COP, central opercular cortex; CRcr‐I, cerebellum crus I; CRcr‐II, cerebellum crus II; FMC, frontal medial cortex; FO, frontal operculum cortex; FOC, frontal orbital cortex; FP, frontal pole; HC, hippocampus; HG, Heschl's gyrus; IC, insular cortex; IFG POp, inferior frontal gyrus, pars opercularis; IFG PTr, inferior frontal gyrus, pars triangularis; IFGt, inferior frontal gyrus, temporooccipital; ITGp, inferior temporal gyrus, posterior; ITGt, inferior temporal gyrus, temporooccipital; JLC, juxtapositional lobule cortex; LOCi, lateral occipital cortex, inferior; LOCs, lateral occipital cortex, superior; MFG, middle frontal gyrus; MTGa, middle temporal gyrus, anterior; MTGp, middle temporal gyrus, posterior; MTGt, middle temporal gyrus, temporooccipital; OFC, occipital fusiform gyrus; OP, occipital pole; PAC, paracingulate gyrus; PC, precuneous cortex; pCG, cingulate gyrus, posterior; PGp, parah [file HBM-46-e70415-s003.docx]

| **Clust no** | **Size (mm^3^**) | **X** | **Y** | **Z** | **ALE** | **P** | **Z** | **Cytoarchitecture** | **% cyto** | **Assignment** | **Hem.** | **Macroanatomy** | **% macro** |
| --- | --- | --- | --- | --- | --- | --- | --- | --- | --- | --- | --- | --- | --- |
| **Semantic tasks, high demands > Non-semantic or less semantic baseline, high demands** | | | | | | | | | | | | | |
| *ALE-analysis, cluster forming threshold: p < .001; cluster extent correction: FWE p < .001* | | | | | | | | | | | | | |
| 1 | 22832 | -54 | -40 | 2 | 0.159 | - | - | - | - | NA | Left | MTGp | 26 |
| 1 | 22832 | -56 | -2 | -16 | 0.098 | - | - | Area TE 5 | 78 | HA | Left | MTGa | 42 |
| 1 | 22832 | -40 | -46 | -20 | 0.088 | - | - | Area FG4 | 72 | HA | Left | TOFC | 33 |
| 1 | 22832 | -48 | -54 | -14 | 0.083 | - | - | Area FG4 | 58 | HA | Left | ITGt | 57 |
| 1 | 22832 | -32 | -36 | -18 | 0.080 | - | - | Area FG3 | 11 | NHA | Left | TFCp | 65 |
| 1 | 22832 | -48 | 16 | -26 | 0.049 | - | - | - | - | NA | Left | TP | 65 |
| 2 | 17752 | -48 | 24 | 18 | 0.131 | - | - | Area 45 | 14 | NHA | Left | IFG PTr | 25 |
| 2 | 17752 | -50 | 28 | 0 | 0.108 | - | - | Area OP9 | 67 | HA | Left | IFG PTr | 42 |
| 2 | 17752 | -44 | 30 | -10 | 0.084 | - | - | Area OP9 | 15 | NHA | Left | FOC | 44 |
| 2 | 17752 | -42 | 4 | 26 | 0.054 | - | - | Area 44 | 18 | NHA | Left | IFG POp | 25 |
| 3 | 3232 | 36 | 24 | -6 | 0.080 | - | - | Area Id7 | 17 | NHA | Right | FOC | 65 |
| 3 | 3232 | 34 | 38 | -10 | 0.050 | - | - | Area Fo7 | 59 | HA | Right | FP | 61 |
| 4 | 2752 | -4 | -58 | 18 | 0.066 | - | - | - | - | NA | Left | PC | 68 |
| 4 | 2752 | -12 | -50 | 2 | 0.039 | - | - | - | - | NA | Left | CGp | 41 |
| 5 | 2472 | -24 | -12 | -16 | 0.074 | - | - | - | - | NA | Left | Left HC | 40 |
| 6 | 2112 | 10 | -78 | -28 | 0.062 | - | - | - | - | NA | Right | CRcr-I | 76 |
| 6 | 2112 | 22 | -80 | -34 | 0.045 | - | - | - | - | NA | Right | CRcr-II | 65 |
|  | | | | | | | | | | | | | |
| **Semantic tasks, low demands > Non-semantic or less semantic baseline, low demands** | | | | | | | | | | | | | |
| *ALE-analysis, cluster forming threshold: p < .001; cluster extent correction: FWE p < .001* | | | | | | | | | | | | | |
| 1 | 19352 | -52 | -40 | 4 | 0.105 | - | - | - | - | NA | Left | - | - |
| 1 | 19352 | -56 | -6 | -14 | 0.087 | - | - | Area TE 5 | 69 | HA | Left | MTGa | 38 |
| 1 | 19352 | -58 | -10 | -8 | 0.084 | - | - | Area TE 4 | 66 | HA | Left | STGa | 27 |
| 1 | 19352 | -48 | 14 | -26 | 0.080 | - | - | - | - | NA | Left | TP | 59 |
| 1 | 19352 | -44 | -58 | 22 | 0.067 | - | - | Area PGa (IPL) | 0 | NHA | Left | AG | 55 |
| 2 | 11952 | -48 | 16 | 22 | 0.075 | - | - | Area 44 | 28 | NHA | Left | IFG POp | 53 |
| 2 | 11952 | -52 | 30 | 2 | 0.065 | - | - | Area OP9 | 46 | HA | Left | IFG PTr | 66 |
| 2 | 11952 | -46 | 32 | -12 | 0.057 | - | - | Area Fo6 | 24 | NHA | Left | FOC | 52 |
| 2 | 11952 | -54 | 2 | 22 | 0.037 | - | - | Area 44 | 29 | NHA | Left | PRG | 59 |
| 3 | 5328 | 58 | -6 | -10 | 0.067 | - | - | Area TE 4 | 46 | HA | Right | STGa | 32 |
| 3 | 5328 | 52 | 2 | -20 | 0.066 | - | - | Area TE 5 | 83 | HA | Right | STGa | 44 |
| 3 | 5328 | 50 | -18 | -10 | 0.047 | - | - | Area TE 5 | 58 | HA | Right | MTGp | 46 |
| 3 | 5328 | 60 | -10 | 4 | 0.033 | - | - | Area TE 1.0 | 14 | NHA | Right | PT | 42 |
| 4 | 3712 | -42 | -52 | -18 | 0.064 | - | - | Area FG4 | 68 | HA | Left | TOFC | 43 |
| 4 | 3712 | -30 | -36 | -18 | 0.039 | - | - | Area FG3 | 10 | NHA | Left | TFCp | 64 |
| 5 | 1952 | 58 | -32 | 0 | 0.059 | - | - | Area TE 5 | 19 | NHA | Right | MTGp | 35 |
| 6 | 1952 | -46 | 0 | 50 | 0.060 | - | - | - | - | NA | Left | PRG | 46 |
| 6 | 1952 | -52 | -8 | 44 | 0.044 | - | - | - | - | NA | Left | PRG | 58 |
|  |  |  |  |  |  |  |  |  |  |  |  |  |  |
| **Semantic tasks, high demands, Sentences/Narratives > Non-semantic or less semantic baseline, high demands** | | | | | | | | | | | | | |
| *ALE-analysis, cluster forming threshold: p < .001; cluster extent correction: FWE p < .001* | | | | | | | | | | | | | |
| 1 | 13608 | -54 | -40 | 2 | 0.095 | - | - | - | - | NA | Left | MTGp | 26 |
| 1 | 13608 | -56 | -4 | -14 | 0.086 | - | - | Area TE 5 | 71 | HA | Left | STGa | 41 |
| 1 | 13608 | -44 | -60 | 26 | 0.054 | - | - | Area PFm (IPL) | 10 | NHA | Left | AG | 47 |
| 1 | 13608 | -48 | 14 | -26 | 0.026 | - | - | - | - | NA | Left | TP | 59 |
| 2 | 4192 | -50 | 28 | 4 | 0.055 | - | - | Area OP9 | 71 | HA | Left | IFG PTr | 44 |
| 2 | 4192 | -54 | 24 | 12 | 0.051 | - | - | Area 45 | 50 | HA | Left | IFG PTr | 53 |
| 2 | 4192 | -46 | 20 | 24 | 0.030 | - | - | Area 45 | 8 | NHA | Left | IFG POp | 32 |
| 3 | 2144 | -40 | -46 | -20 | 0.046 | - | - | Area FG4 | 72 | HA | Left | TOFC | 33 |
| 3 | 2144 | -40 | -56 | -20 | 0.045 | - | - | Area FG4 | 64 | HA | Left | TOFC | 72 |
| 3 | 2144 | -32 | -38 | -16 | 0.032 | - | - | Area FG3 | 21 | NHA | Left | TFCp | 60 |
|  |  |  |  |  |  |  |  |  |  |  |  |  |  |
| **Semantic tasks, high demands, Single-Words/Word-Pairs > Non-semantic or less semantic baseline, high demands** | | | | | | | | | | | | | |
| *ALE-analysis, cluster forming threshold: p < .001; cluster extent correction: FWE p < .001* | | | | | | | | | | | | | |
| 1 | 14704 | -48 | 24 | 18 | 0.113 | - | - | Area 45 | 14 | NHA | Left | IFG PTr | 25 |
| 1 | 14704 | -46 | 30 | -8 | 0.067 | - | - | Area OP9 | 20 | NHA | Left | FOC | 44 |
| 1 | 14704 | -32 | 24 | -6 | 0.037 | - | - | Area Id7 | 2 | NHA | Left | FOC | 55 |
| 2 | 12072 | -56 | -38 | 0 | 0.078 | - | - | Area TE 5 | 2 | NHA | Left | MTGp | 43 |
| 2 | 12072 | -48 | -54 | -14 | 0.075 | - | - | Area FG4 | 58 | HA | Left | ITGt | 57 |
| 2 | 12072 | -36 | -32 | -20 | 0.066 | - | - | CA1 (Hippocampus) | 15 | NHA | Left | TFCp | 60 |
| 3 | 6016 | -46 | -68 | 26 | 0.068 | - | - | Area PGp (IPL) | 45 | HA | Left | LOCs | 66 |
| 3 | 6016 | -32 | -64 | 40 | 0.064 | - | - | Area hIP6 (IPS) | 39 | HA | Left | LOCs | 54 |
| 3 | 6016 | -34 | -52 | 44 | 0.039 | - | - | Area hIP3 (IPS) | 43 | HA | Left | SPL | 31 |
| 3 | 6016 | -40 | -78 | 36 | 0.033 | - | - | Area PGp (IPL) | 55 | HA | Left | LOCs | 77 |
| 4 | 5896 | -4 | 20 | 48 | 0.106 | - | - | Area 6mr / preSMA | 6 | NHA | Left | PAC | 49 |
| 5 | 2816 | 36 | 24 | -6 | 0.066 | - | - | Area Id7 | 17 | NHA | Right | FOC | 65 |
| 5 | 2816 | 34 | 38 | -10 | 0.044 | - | - | Area Fo7 | 59 | HA | Right | FP | 61 |
| 6 | 1728 | -24 | -14 | -18 | 0.049 | - | - | CA1 (Hippocampus) | 58 | HA | Left | Left HC | 91 |
|  |  |  |  |  |  |  |  |  |  |  |  |  |  |
| **Semantic tasks, low demands, Sentences/Narratives > Non-semantic or less semantic baseline, low demands** | | | | | | | | | | | | | |
| *ALE-analysis, cluster forming threshold: p < .001; cluster extent correction: FWE p < .001* | | | | | | | | | | | | | |
| 1 | 8816 | -54 | 22 | 16 | 0.070 | - | - | Area 45 | 49 | HA | Left | IFG PTr | 42 |
| 1 | 8816 | -48 | 16 | 22 | 0.065 | - | - | Area 44 | 28 | NHA | Left | IFG POp | 53 |
| 1 | 8816 | -40 | 32 | -12 | 0.034 | - | - | Area Fo7 | 23 | NHA | Left | FOC | 45 |
| 2 | 8152 | -56 | -6 | -14 | 0.085 | - | - | Area TE 5 | 69 | HA | Left | MTGa | 38 |
| 2 | 8152 | -48 | 14 | -26 | 0.070 | - | - | - | - | NA | Left | TP | 59 |
| 3 | 7352 | -52 | -40 | 4 | 0.089 | - | - | - | - | NA | Left | - | - |
| 3 | 7352 | -46 | -58 | 20 | 0.049 | - | - | Area PGa (IPL) | 1 | NHA | Left | AG | 49 |
| 3 | 7352 | -50 | -52 | 16 | 0.038 | - | - | - | - | NA | Left | AG | 33 |
| 3 | 7352 | -54 | -58 | 28 | 0.034 | - | - | Area PGa (IPL) | 57 | HA | Left | AG | 58 |
| 3 | 7352 | -64 | -24 | 0 | 0.030 | - | - | Area TE 4 | 39 | HA | Left | STGp | 41 |
| 4 | 5520 | 52 | 2 | -20 | 0.061 | - | - | Area TE 5 | 83 | HA | Right | STGa | 44 |
| 4 | 5520 | 56 | -6 | -12 | 0.055 | - | - | Area TE 4 | 55 | HA | Right | STGa | 33 |
| 4 | 5520 | 50 | 12 | -26 | 0.049 | - | - | Area TE 5 | 10 | NHA | Right | TP | 84 |
| 4 | 5520 | 50 | -18 | -10 | 0.047 | - | - | Area TE 5 | 58 | HA | Right | MTGp | 46 |
| 5 | 1536 | -4 | 20 | 48 | 0.040 | - | - | Area 6mr / preSMA | 6 | NHA | Left | PAC | 49 |
| 5 | 1536 | -6 | 4 | 56 | 0.033 | - | - | Area 6mr / preSMA | 73 | HA | Left | JLC | 65 |
| 5 | 1536 | 6 | 16 | 44 | 0.033 | - | - | Area 6mr / preSMA | 0 | NHA | Right | PAC | 66 |
|  |  |  |  |  |  |  |  |  |  |  |  |  |  |
| **Semantic tasks, low demands, Single-Words/Word-Pairs > Non-semantic or less semantic baseline, low demands** | | | | | | | | | | | | | |
| *ALE-analysis, cluster forming threshold: p < .001; cluster extent correction: FWE p < .001* | | | | | | | | | | | | | |
| 1 | 3560 | -40 | -40 | -14 | 0.036 | - | - | Area FG3 | 37 | HA | Left | - | - |
| 1 | 3560 | -50 | -44 | -18 | 0.028 | - | - | Area FG4 | 51 | HA | Left | ITGp | 28 |
| 1 | 3560 | -54 | -42 | -16 | 0.027 | - | - | - | - | NA | Left | - | - |
| 1 | 3560 | -30 | -40 | -18 | 0.019 | - | - | Area FG3 | 52 | HA | Left | TFCp | 61 |
| 2 | 1656 | -58 | -32 | 2 | 0.035 | - | - | Area TE 4 | 18 | NHA | Left | STGp | 30 |

**Supplementary Table 8. All activation clusters and local maxima for Verbal Semantic Cognition, split by external task demands (Separate ALE Meta-Analyses).** Coordinates x, y and z reported in the MNI coordinate system; “Clust no”: Cluster number in the individual contrast; “ALE”: Activation Likelihood Estimation values output from Ginger ALE, along with P and Z values; “Cytoarchitecture”: cytoarchitectonic information for foci assigned by the JuBrain Anatomy Toolbox (SPM), based on the Maximum Probability Map; “% cyto”: probability of the coordinate falling into the specified Cytoarchitecture, as an output of the Anatomy Toolbox; “Assignment”: Type of assignment of coordinate into the specified Cytoarchitecture, as an output of the Anatomy Toolbox – HA: Hard Assignment, NHA: No Hard Assignment, NA: No Assignment “Hem”: hemisphere; “Macroanatomy”: Assignment of the foci and to the Harvard-Oxford microanatomical atlas; “% macro”: probability of the coordinate falling into the assigned region by the Harvard-Oxford microanatomical atlas; "aCG": Cingulate Gyrus, anterior; "AG": Angular Gyrus; "AMYG": Amygdala; "COP": Central Opercular Cortex; "CRcr-I": Cerebellum Crus I; "CRcr-II": Cerebellum Crus II; "FMC": Frontal Medial Cortex; "FO": Frontal Operculum Cortex; "FOC": Frontal Orbital Cortex; "FP": Frontal Pole; "HC": Hippocampus; "HG": Heschl's Gyrus; "IC": Insular Cortex; "IFG POp": Inferior Frontal Gyrus, pars opercularis; "IFG PTr": Inferior Frontal Gyrus, pars triangularis; "IFGt": Inferior Frontal Gyrus, temporooccipital; "ITGp": Inferior Temporal Gyrus, posterior; "ITGt": Inferior Temporal Gyrus, temporooccipital; "JLC": Juxtapositional Lobule Cortex; "LOCi": Lateral Occipital Cortex, inferior; "LOCs": Lateral Occipital Cortex, superior; "MFG": Middle Frontal Gyrus; "MTGa": Middle Temporal Gyrus, anterior; "MTGp": Middle Temporal Gyrus, posterior; "MTGt": Middle Temporal Gyrus, temporooccipital; "OFC": Occipital Fusiform Gyrus; "OP": Occipital Pole; "PAC": Paracingulate Gyrus ; "PC": Precuneous Cortex; "pCG": Cingulate Gyrus, posterior; "PGp": Parahippocampal Gyrus, posterior; "POC": Parietal Operculum Cortex; "PP": Planum Polare; "PRG": Precentral Gyrus; "PT": Planum Temporale; "RC": Right Caudate; "SFG": Superior Frontal Gyrus; "SGp": Supramarginal Gyrus, posterior; "SPL": Superior Parietal Lobule; "STGa": Superior Temporal Gyrus, anterior; "STGp": Superior Temporal Gyrus, posterior; "STGs": Superior Temporal Gyrus, superior; "TFCp": Temporal Fusiform Cortex, posterior; "TOFC": Temporal Occipital Fusiform Cortex; "TP": Temporal Pole.
